# Structure–Activity Relationship of *N*-Phenylthieno[2,3-*b*]pyridine-2-carboxamide Derivatives Designed as Forkhead Box M1 Inhibitors: The Effect of Electron-Withdrawing and Donating Substituents on the Phenyl Ring

**DOI:** 10.3390/ph15030283

**Published:** 2022-02-24

**Authors:** César Sebastian Huerta-García, David J. Pérez, Carlos A. Velázquez-Martínez, Seyed Amirhossein Tabatabaei Dakhili, Antonio Romo-Mancillas, Rafael Castillo, Alicia Hernández-Campos

**Affiliations:** 1 Departamento de Farmacia, Facultad de Química, Universidad Nacional Autónoma de México, Mexico City 04510, Mexico; cshuertagarcia@gmail.com (C.S.H.-G.); rafaelc@unam.mx (R.C.); 2 Faculty of Pharmacy and Pharmaceutical Sciences, University of Alberta, Edmonton, AB T6E 2E1, Canada; perezgom@ualberta.ca (D.J.P.); velazque@ualberta.ca (C.A.V.-M.); stabatab@ualberta.ca (S.A.T.D.); 3 Unidad Radiofarmacia-Ciclotrón, División de Investigación, Facultad de Medicina, Universidad Nacional Autónoma de México, Mexico City 04510, Mexico; 4 Laboratorio de Diseño Asistido por Computadora y Síntesis de Fármacos, Facultad de Química, Universidad Autónoma de Querétaro, Centro Universitario, Querétaro 76010, Mexico; ruben.romo@uaq.mx

**Keywords:** thieno[2,3-*b*]pyridines, FOXM1, transcription factor, cancer

## Abstract

We report synthesis, characterization, biological evaluation, and molecular-docking studies of 18 thieno[2,3-*b*]pyridines with a phenylacetamide moiety at position 2, which is disubstituted with F, Cl, Br, or I at position 4, and with electron-withdrawing and electron-donating groups (-CN, -NO_2_, -CF_3_, and -CH_3_) at position 2, to study how the electronic properties of the substituents affected the FOXM1-inhibitory activity. Among compounds **1**–**18**, only those bearing a -CN (regardless of the halogen) decreased FOXM1 expression in a triple-negative breast cancer cell line (MDA-MB-231), as shown by Western blotting. However, only compounds **6** and **16** decreased the relative expression of FOXM1 to a level lower than 50%, and hence, we determined their anti-proliferative activity (IC_50_) in MDA-MB-231 cells using the MTT assay, which was comparable to that observed with **FDI-6**, in contrast to compound **1**, which was inactive according to both Western blot and MTT assays. We employed molecular docking to calculate the binding interactions of compounds **1**–**18** in the FOXM1 DNA-binding site. The results suggest a key role for residues Val296 and Leu289 in this binding. Furthermore, we used molecular electrostatic potential maps showing the effects of different substituents on the overall electron density.

## 1. Introduction

**Figure 1:**
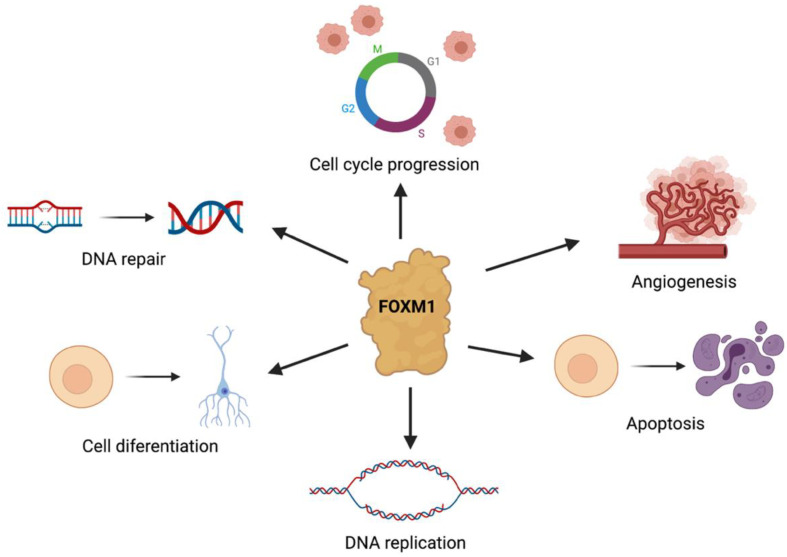
The role of FOXM1 in cancer initiation and cancer progression. FOXM1 is involved in cell proliferation, cell-cycle progress, cell differentiation, DNA replication, DNA-damage repair, tissue homeostasis, angiogenesis, and apoptosis.

The forkhead box M1 protein (FOXM1) belongs to the forkhead superfamily of transcription factors, and it regulates the expression of some genes related to cell-cycle progression. FOXM1 regulates a broad spectrum of biological processes, including cell proliferation and differentiation, DNA replication, DNA repair, tissue homeostasis, angiogenesis, and apoptosis (Figure 1) [1,2]. High levels of the FOXM1 protein are found in proliferating cells, but these levels are significantly lower in differentiated or quiescent cells [3]. However, high levels of the protein are found in most types of cancer [3], suggesting that it plays an essential role in tumorigenesis [2]. FOXM1 is also known as the master regulator of oncogenesis [3,4,5], making it an attractive target for the design of anticancer compounds. Despite these potentially attractive features, targeting transcription factors (including FOXM1) is still challenging, since they lack common “binding sites” frequently used for small molecules [6].

**Figure 2:**
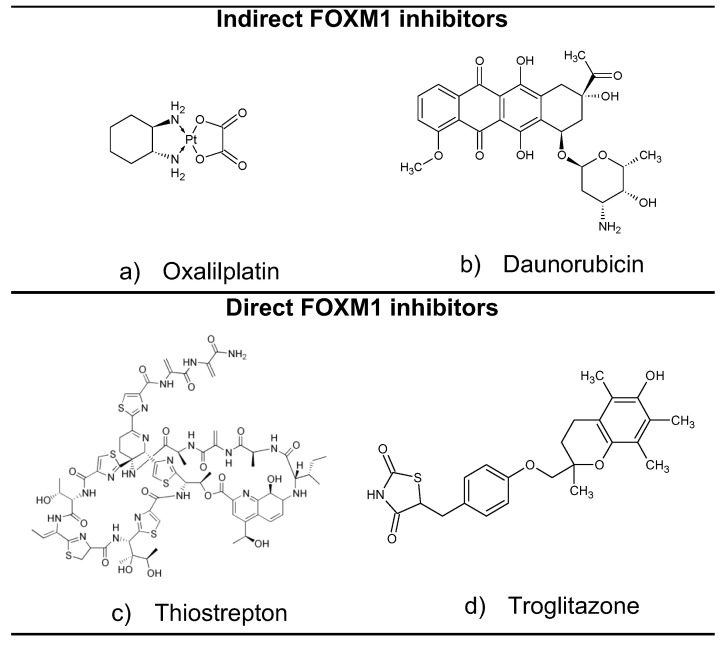
Previously reported indirect and direct FOXM1 inhibitors. Indirect FOXM1 inhibitors: (**a**) oxalilplatin and (**b**) daunorubicin. Direct FOXM1 inhibitors: (**c**) thiostrepton and (**d**) troglitazone.

Thus far, several experimental molecules have been shown to exert FOXM1-inhibitory activity in vitro. From a mechanistic point of view, molecules that target the transcriptional activity of FOXM1 can act directly or indirectly. In this sense, indirect inhibitors target upstream proteins that normally promote FOXM1 expression, while direct inhibitors must dissociate the FOXM1–DNA complex [6]. Some representative examples of indirect and direct FOXM1 inhibitors are shown in Figure 2. Among the most important ones, we can observe troglitazone, which belongs to the group of drugs known as thiazolidinediones, whose original function was as agonists of the transcription factor PPARγ in the treatment of diabetes [3]. On the other hand, we note thiostrepton, which is a natural product isolated from *Streptomyces azureus* [7].

**Figure 3:**
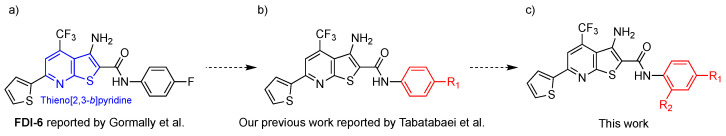
Previous work that led to the design of the compounds presented in this work. (**a**) FDI-6, a thieno[2,3-*b*]pyridine, previously reported by Gormally et al. [8] as a direct FOXM1 inhibitor; (**b**) Previous FDI-6 derivatives reported by our research group [9]; (**c**) Compounds studied in this work.

Gormally et al. identified some molecules that bind to and disrupt the FOXM1 transcriptional program, known as “fork domain inhibitors” (FDIs), from which they identified and validated the drug **FDI-6** (Figure 3a), a thieno[2,3-*b*]pyridine derivative [8]. Our group proposed a mechanism for the binding of **FDI-6** with FOXM1-DNA binding domain (FOXM1-DBD) by using molecular modeling; we hypothesized that the presence of a halogen at position 4 of the phenyl ring was essential for the interaction with Arg297 (Figure 3b) [6], so we tested this hypothesis experimentally and proved it [9].

In medicinal chemistry and drug design, pyridine derivatives are interesting compounds due to their potential to exert a great variety of biological activities [10]. Of the pyridine derivatives, the fused analogs are of greater interest than the corresponding monocyclic compounds. The chemical properties of the fused rings increase the possibilities of varying the pharmacophoric groups in different positions, which may impact their ability to interact with a wide variety of biological targets. In addition, fused systems are also interesting from a theoretical and biological activity point of view due to the possibility of having π-excessive and π-deficient aromatic rings together [11]. The thieno[2,3-*b*]pyridines (Figure 3a), known since 1913, are molecules with a plethora of biological activities, which include anti-inflammatory, antidepressant, antimicrobial [10,11], and antitumor [8,12,13] activities, and have been used for the treatment of diseases of the central nervous system [14].

In this work, we studied how using different electron-withdrawing and electron-donating substituents at position 2 of the phenyl ring, combined with a halogen at position 4, affected the FOXM1-inhibitory activity. For this purpose, we synthesized 18 thieno[2,3-*b*]pyridines (**FDI-6** derivatives), bearing -CN, -NO_2_, -CF_3_, and -CH_3_ at position 2 and a halogen at position 4 (R_1_ = -F, -Cl, -Br, and -I, Figure 3c). We evaluated these molecules as FOXM1 inhibitors in triple-negative breast cancer cells (MDA-MB-231). We screened the most active compounds to determine their anti-proliferative activity (in MDA-MB-231), as well as molecular-docking studies. Additionally, to understand how the combined effect of the halogen and the substituent affected the electronic properties of molecules **1**–**18**, we calculated the molecular electrostatic potential (MEP) maps and polar surface area (PSA).

## 2. Results and Discussion

### 2.1. Synthesis of N-Phenylthieno[2,3-b]pyridine-2-carboxamide Derivatives

**Scheme 1:**
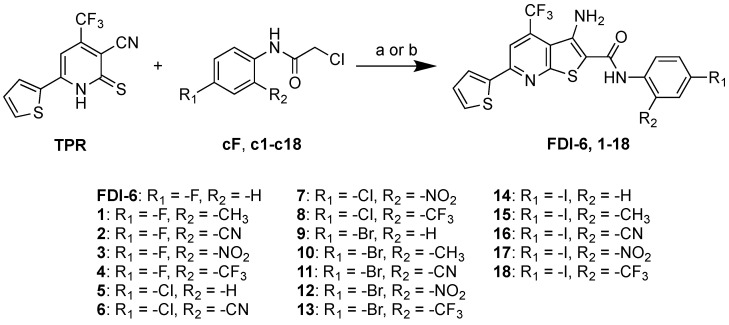
Chemical synthesis of FDI-6 and derivatives **1**–**18**. (a) Reagents and conditions: K_2_CO_3_, EtOH, 90 °C (conventional heating), and 3 h. (b) Reagents and conditions: K_2_CO_3_, EtOH, 90 °C (μW heating), and 3 h.

For the synthesis of compounds **1**–**18**, we adapted our previously reported method [9] (Scheme 1). A Thorpe–Ziegler-type isomerization, between **TPR** and the appropriately substituted 2-chloro-*N*-phenylacetamides, which we also prepared (see the Supplementary Materials) (**cF** and **c1**–**c18**), using ethanol as the reaction solvent and conventional or microwave heating, produced compounds (**FDI-6** and **1**–**18**) with an overall 80–90% yield. Both of the methods used were effective for obtaining the compounds at similar yields (see Table S1 for yield comparison); however, the products obtained through microwave heating required less purification. Most of the compounds were purified by recrystallization with a toluene: DMF mixture and characterized by ^1^H and ^13^C NMR, IR spectroscopy, and mass spectrometry (see the Supplementary Materials for full details).

### 2.2. Determination of FOXM1’s Relative Expression

To assess the FOXM1-inhibitory activity of the parent compound and all the final compounds (**FDI-6** and **1**–**18**), and considering that FOXM1 modulates its transcriptional expression [15], we measured the cell expression levels of the FOXM1 protein by Western blotting, after incubating MDA-MB-231 cells with test compounds (40 μM, for 24 h). This cell line is an appropriate model due to its overexpression of the FOXM1 protein compared to other breast cancer cell lines [16], and prompted by this idea, we have recently reported the potential to use FOXM1 inhibitors to detect triple-negative breast cancer [17]. We conducted Western blot experiments assuming that a drug-dependent decrease in the FOXM1 level could be due to a drug-induced dissociation of the nuclear FOXM1–DNA complex, which in turn would suggest transcriptional inhibition, as we determined in previous work [18].

**Figure 4:**
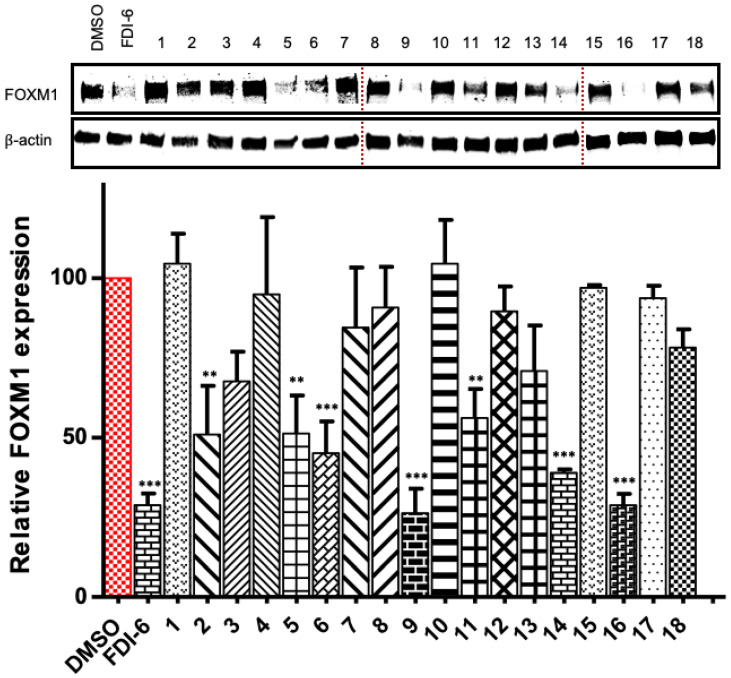
Western blot analysis of FOXM1 protein levels, which were decreased by the test compounds (40 µM; 24 h; MDA-MB-231 cell line). The average values in all experiments were calculated after three independent experiments (*n* = 3) and are expressed as means ± SEMs; *p* values were calculated by one way ANOVA (** = *p* ≤ 0.01; *** = *p* ≤ 0.001).

We observed a significant decrease in FOXM1 protein levels in MDA-MB-231 breast cancer cells treated with compounds **2**, **5**, **6**, **9**, **11**, **14**, and **16** (Figure 4). Based on a simple structure–activity relationship study, we can make a few preliminary statements to describe the effect of substituting a specific functional group on FOXM1 expression levels in this cell line. We observed that, in those molecules bearing -CF_3_, -NO_2_, and -CH_3_, regardless of the halogen at position 4 of the phenylacetamide ring, the FOXM1-inhibitory activity was lost (they were inactive), whereas those bearing a -CN (**2**, **6**, **11**, and **16**) at position 2 significantly decreased FOXM1’s protein levels. However, only compounds **6** and **16** decreased FOXM1’s relative expression (lower than ~50%) in ranges similar to those of **FDI-6** and the parent molecules **5**, **9**, and **14**.

**Figure 5:**
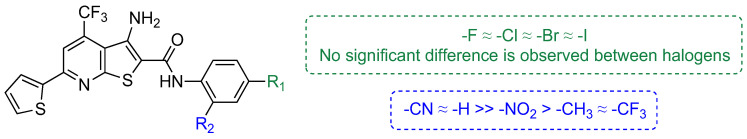
Summary of structure–activity relationship.

From these results, we can draw some preliminary conclusions: (i) there is no significant difference in terms of the FOXM1-inhibitory activity among different halogens in compounds **2**, **6**, **11**, and **16**, confirming our previous findings [9], and (ii) only the combination of a halogen and the -CN at positions 4 and 2 of the phenylacetamide group, respectively, resulted in FOXM1-inhibitory activity. It is worth mentioning that the apparent lack of FOXM1-inhibitory activity of those compounds bearing a -CF_3_, -CH_3_, and -NO_2_ at position 2 could be related to our experimental qualitative data, showing that they were less soluble in aqueous media than their precursors and **2**, **6**, **11**, and **16**, which may have impaired their dissolution in the cell medium and, therefore, their FOXM1-inhibitory activity. In our previous study [9], we observed that **FDI-6** derivatives bearing a -CF_3_ and -CH_3_ instead of a halogen at position 4 of the phenylacetamide ring were active according to an EMSA assay (cell-free assay), though inactive in vitro. This could partially explain the lack of activity of the tested molecules, although it is evident that varying the substitution on the ring affected the biological activity, showing that only the combined electronic effect of the -CN and the halogen favored FOXM1 inhibition. A summary of the structure–activity relationship of the derivatives prepared is shown in Figure 5.

### 2.3. Cytotoxic Activity Determination

Based on literature reports [19] and our previous studies [9,18], which show that FOXM1-inhibitory activity affects cell proliferation and development, we screened compounds **6** and **16** (which significantly decreased FOXM1’s relative expression, *** = *p* ≤ 0.001) and compared them to the inactive compound **1** in the MDA-MB-231 cell line using the MTT assay to determine the inhibitory concentration 50 (IC_50_). It is worth mentioning that we chose derivative **1** as the “inactive model compound”, since other inactive ones (e.g., **10**, **15**, and **17**) could not be screened due to their low solubility in DMSO, which made it difficult to prepare the stock solution at the required concentration for the MTT assay.

After incubating cells with the test molecules with increasing concentrations, we observed a correlation with the FOXM1 inhibition determined in the Western blots (Table 1). Compounds **6** and **16** showed anti-proliferative activity that was comparable to that of **FDI-6**. On the other hand, compound **1** (inactive according to Western blotting; vide supra) showed the highest IC_50_ compared to that of **FDI-6** and compounds **6** and **16**. These results suggest a correlation between FOXM1 inhibition and anti-proliferative activity. This correlation may also indicate that the mechanism by which compounds **6** and **16** inhibit cell proliferation could be related to FOXM1 inhibition; however, this will require further experiments for confirmation.

### 2.4. Molecular Docking in FOXM1’s DNA-Binding Domain

To further analyze the chemical structures of the compounds and correlate them with their FOXM1-inhibitory activity, we carried out molecular-docking calculations for docking between the FOXM1-DBD, which we previously identified as the potential binding site for FOXM1 inhibitors [6], and the 19 synthesized compounds discussed in this work. We used the docking module of the Molecular Operating Environment (MOE) software to carry out the calculations [20]. The final docking score (S, given in kcal/mol) was calculated using the Generalized-Born Volume Integral/Weighted Surface Area binding free energy scoring function (GBVI/WSA dG). The GBVI/WSA dG is a forcefield-based scoring function that estimates the free energy of binding of the ligand from a given binding mode [21], which, along with the MOE docking engine, can reproduce top-scoring poses that approximate experimentally determined binding poses [22]. The final docking scores (S) of compounds **FDI-6** and **1**–**18** (henceforth referred to as ligands) docking to the FOXM1-DBD, determined by calculating a possible binding mode (referred to as the pose), are shown in Table 2. In general, a lower S score (that is, a higher negative free energy of the binding) indicates a more favorable pose. For a more precise analysis of the results, the effects of substituents on the phenylacetamide ring (henceforth referred to as the ring) are only compared to those of their parent compound and derivatives bearing the same halogen.

The docking score (S value) calculated for compounds **1**–**18** was more negative than that calculated for the parent compound **FDI-6**. In accord with the docking results, having a substituent at position 2 of the phenylacetamide ring improved the interaction with the FOXM1-DBD for almost all the derivatives, resulting in more negative S values than those of their parent compounds regardless of the halogen, except for compound **15**. It is interesting to note that the increase in binding energy is especially noticeable for the electron-withdrawing substituents such as -NO_2_ or -CN (compounds **2**, **3**, **6**, **7**, **11**, **12**, **16**, and **17**), when compared to those bearing the -CF_3_ substituent (compounds **4**, **8**, **13**, and **18**) or the electron-releasing -CH_3_ moiety (compounds **1**, **10**, and **15**), which are less polar than the former and showed a binding energy similar to that of the parent compounds **FDI-6**, **5**, **9**, and **14**. In this regard, compounds with a -CN substituent, which are the ones that showed the highest FOXM1-inhibitory activity (vide supra), are effectively those with the most favorable docking scores (lowest S scores) compared to the halogen group to which they belong.

**Figure 6:**
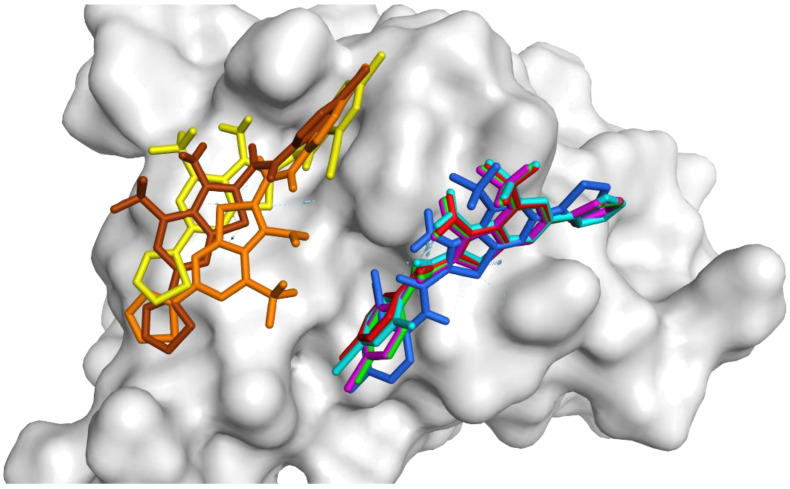
Docking poses from the -CN (**2** (blue), **6** (yellow), **11** (orange), and **16** (brown)) and -CH_3_ (**1** (green), **10** (purple), and **15** (light blue)) substituted compounds compared to **FDI-6** (red) in the FOXM1-DBD.

**Figure 7:**
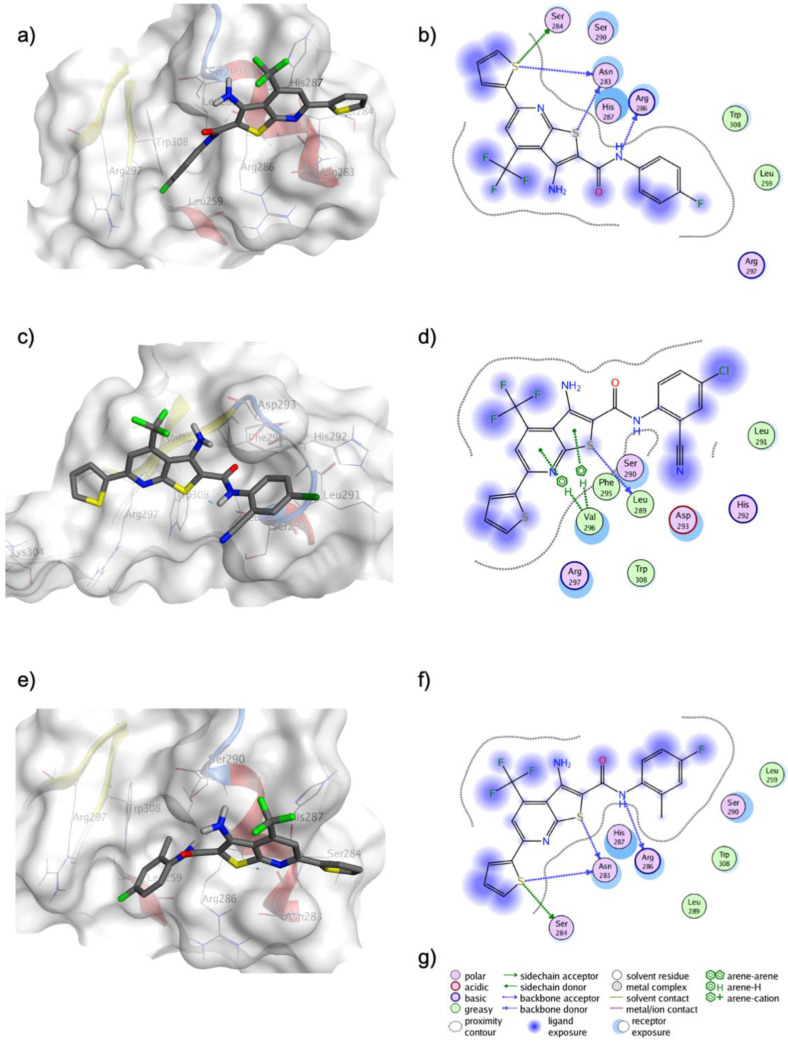
Docking poses and ligand interaction diagrams for compounds **FDI-6**; **1**, an example of an inactive compound; and **6**, an example of an active compound. (**a**) Docking pose of compound **FDI-6**. (**b**) Ligand interaction diagram for compound **FDI-6**. (**c**) Docking pose of compound **6**. (**d**) Ligand interaction diagram for compound **6**. (**e**) Docking pose of compound **1**. (**f**) Ligand interaction diagram for compound **1**. (**g**) Ligand interaction diagram legend.

As we can see in Figure 6, while **FDI-6** and compound **1** adopted the expected pose in which fluorine interacts with Arg297 and the thiophene ring with His287 (Figure 7a,b), compounds **6**, **11**, and **16** bound in a different pose, though still in the DBD region. As depicted in Figure 7c,d, in compound **6**, the -CN as such does not show any relevant interaction with residues of the DBD; however, the electron-withdrawing effect on the phenyl ring (exerted by resonance), added to the inductive effect generated by the chlorine atom, changes the electron density of the phenyl ring, forcing it to bind differently from **FDI-6**. The binding of **6** led us to identify two interactions involving the thieno[2,3-*b*]pyridine ring: a π–H bond to the Val296 sidechain and a conventional sulfur–hydrogen bond to the backbone from Leu289.

The compounds that did not show FOXM1-inhibitory activity, such as those bearing -CH_3_ or -CF_3_ substituents (vide supra), were consistently those with the least favorable docking scores within the halogen group to which they belong (Table 2). As we can see in Figure 6, all the compounds with -CH_3_ substituents adopted the same pose as **FDI-6**, and even if the methyl group did help to introduce that side of the ring into the hydrophobic pocket of the FOXM1-DBD (Figure 7e,f), no other new interaction seems to have been favored. However, this information is useful regarding the electronic properties that the substituent in this position must have to exhibit a favorable interaction with FOXM1. Finally, when comparing the results between -CN and -CH_3_ substituted compounds, it seems that the steric effect due to the size of the substituent is not the key to FOXM1-inhibitory activity. However, the docking results suggest that the electronic properties of the substituent (i.e., electron-withdrawing or electron-donating) affect the electron density of the phenylacetamide ring and its interactions with key residues of the FOXM1-DBD, which seem to be fundamental in FOXM1-inhibitory activity. The substituent also affects the polarity and water solubility of the synthesized compounds, which help them to interact with the solvent, allowing other lipophilic fragments of the structure to interact more favorably with the receptor.

**Figure 8:**
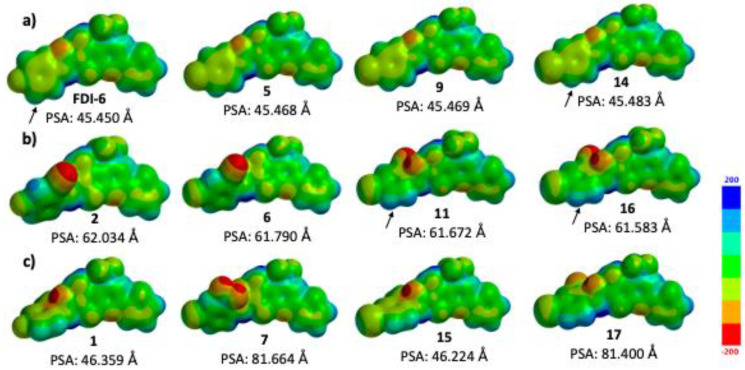
Polar surface area and MEP maps superimposed onto the total electron density at a value of 0.002 e^−^/au^3^, for (**a**) **FDI-6**, **5**, **9**, and **14**; (**b**) **2**, **6**, **11**, and **16**; and (**c**) **1**, **7**, **15**, and **17**. Blue, red, and green colors show positive, negative, and neutral MEP regions, respectively. The black arrows indicate those blue regions (MEP positive values) in the aromatic protons, adjacent to the amide proton.

Due to this, we also calculated the molecular electrostatic potential (MEP) maps and the polar surface areas to depict how the electronic delocalization caused by the -CN moiety of compounds **6** and **16** affected the binding interactions with FOXM1 in comparison to **FDI-6** and inactive compounds (Table 3 and Figure 8a–c) [23,24]. The MEP maps show a highly delocalized electron density in **FDI-6**, with large zero value regions of MEP prone to forming hydrophobic interactions (green color), showing few blue-color areas (positive value regions of MEP), which are poor in electron density (i.e., prone to nucleophilic attack). The optimized structures of the parent molecules show a planar geometry (**FDI-6**, **5**, **9**, and **14**; Figure 8a); however, the presence of a substituent in **1**, **2**, **6**, and **16** causes the phenylacetamide ring to rotate and orientate “out of the plane”, possibly due to a steric clash between carbonyl and -CH_3_ or -CN (this torsion can be also seen in all the compounds bearing a substituent; Figure 8b,c). The electron-withdrawing effect of -CN on the phenylacetamide ring of **2, 6, 11,** and **16** causes the formation of an electron-rich region (red color, negative MEP value from −160 to −129 kJ/mol), localized over itself and the carbonyl moiety, while inducing an area of a low electronic density over the aromatic protons adjacent to the amide (see the captions in Figure 8a–c and Table 3). The mentioned effects of -CN on the electronic density of **2, 6, 11**, and **16** can be easily noticed when comparing their MEP values to those of **FDI-6** (refer to Table 3 and Table S5 for a detailed list of MEP values).

**Figure 9:**
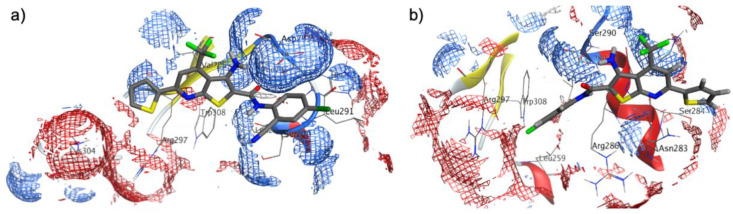
FOXM1 MEP and its interaction with compounds (**a**) **6** and (**b**) **FDI-6**. Electrostatic potentials are indicated by the red and blue meshes, with blue representing electronic-donor areas and red representing electronic-acceptor areas.

As we can observe in Figure 9a, the low electron density on the phenylacetamide ring due to -CN in compound **6** (with an MEP value ranging from −7.0 to 50.0 kJ/mol in contrast to **FDI-6**’s: −50.0 to 17.4 kJ/mol) favors its interactions with a high-electron-density area of the FOXM1-DBD, created mainly by Asp293 and Ser290. Such interactions with the mentioned residues do not form in the binding of **FDI-6** or compounds with a similar binding pose to **FDI-6**’s (Figure 9b). The effects of -CN on the overall MEP of the phenylacetamide ring from **6**, **11**, and **16**, with MEP values ranging from −23.2 to 54.7 kJ/mol, help to explain their different modes of binding to FOXM1 (compared to that of compound **2**), in which the -CN combined with -F does not seem to affect its binding to FOXM1. In compound **1**, -CH_3_ makes the carbonyl moiety richer in electron density than in **FDI-6** (MEP value of −167.6 kJ/mol vs. **FDI-6**’s −112.0 kJ/mol), which also increases the lipophilicity compared to **6** and **16** (according to the values of the PSA; see Table 3 for the values).

On a final note, the MEP maps show that the electron-withdrawing effect of the -NO_2_ group on the carbonyl moiety and phenylacetamide ring of compounds **7** and **17** is not as strong as that of -CN on **6**, **11**, and **16** (Figure 8c and Table 3). This is consistent with the results of molecular docking (Figure 6) and what was observed in the MEP of interactions with the protein (Figure 9), where this difference in electronic density is possibly one of the factors that prevents the compounds with -NO_2_ groups from being able to bind in the same cavity in which -CN compounds favorably bind.

## 3. Materials and Methods

### 3.1. Organic Synthesis: General

Microwave reactions were performed in a Biotage Initiator + Microwave Synthesizer (Upsala, Sweden). The reactions were monitored by thin-layer chromatography (TLC) on 0.2 pre-coated silica gel 60 F_254_ plates (Merck, Darmstadt, Germany). Flash-chromatography purifications were carried out in a CombiFlash EZ Prep equipment (Teledyne, Lincoln, NE, USA). The melting points (mp) were determined on a Büchi B-540 apparatus (Meierseggstrasse, Switzerland) and are uncorrected.

^1^H NMR and ^13^C NMR spectra were recorded either on a 9.4 T Varian VNMRS equipped with a Broad Band Switchable probe of two radio-frequency channels (^1^H/^19^F) (^31^P/^15^N) (Palo Alto, CA, USA) or in a 14.1 T Bruker Avance III equipped with a SmartProbe (Billerika, MA, USA). The deuterated solvents used were dimethyl sulfoxide (DMSO-*d_6_*) from Cambridge Isotope Laboratories (Tewksbury, MA, USA) and chloroform (CDCl_3_) from Sigma-Aldrich (Darmstadt, Germany). The chemical shifts (δ) are given in ppm relative to tetramethylsilane (Me_4_Si, δ = 0) in DMSO-*d*_6_; *J* values are given in Hz. The following abbreviations are used: s, singlet; bs, broad signal; d, doublet; dd, doublet of doublet; t, triplet; m, multiplet.

Mass spectroscopy was performed either through electronic-impact mass spectroscopy (EIMS) using a gas chromatograph, the Perkin Elmer Clarus 680 (Mexico City, Mexico, with a 30 m column and a DB5 stationary phase, coupled to a mass spectrometer, the Perkin Elmer Clarus SQ 8 C, using 200 °C and 70 eV in the ionization chamber and a spectral window between 33 and 500 u, or through high-resolution mass spectrometry (HRMS) on a Perkin Elmer AxION 2 TOF (Mexico City, Mexico) coupled to an AxION DSA module using APCI as the ionization technique, with a crown temperature of 280 °C and 3 μA, using N_2_ as the drying gas at 4 L/min and a spectral window between 50 and 3000 u.

IR spectra were determined using an FT-IR Spectrum 400 from Perkin Elmer (Mexico City, Mexico) equipped with the Perkin Elmer ATR universal accessory, with a resolution of 4 cm^−1^.

### 3.2. General Method for the Synthesis of the Final Derivatives (**FDI-6** and **1**–**18**): Conventional Heating

The appropriate 2-chloro-*N*-phenylacetamide (**cF**, **c1**–**c18**) (1 equiv.), 6-(thiophen-2-yl)-2-thioxo-4-(trifluoromethyl)-1,2-dihydropyridine-3-carbonitrile (**TPR**) (1.2 equiv.), and K_2_CO_3_ (2.5 equiv.) were dissolved using EtOH in a 100 mL round-bottomed flask equipped with a magnetic stirrer. A condenser was fitted to the flask, and the reaction was heated to 95 °C and stirred for 3 h. The reaction was monitored by TLC using Hex/EtOAc 90:10 and stopped once the starting materials were consumed. The reaction mixture was allowed to reach room temperature to yield the product as a precipitate that was then poured into an ice-water mixture, filtered off, and dried under vacuum for 12 h. If needed, the products were recrystallized.

#### 3.2.1. 3-Amino-*N*-(4-fluorophenyl)-6-(thiophen-2-yl)-4-(trifluoromethyl)thieno[2,3-*b*]pyridine-2-carboxamide (**FDI-6**)

TPR (996 mg, 3.47 mmol), cF (500 mg, 2.67 mmol), K_2_CO_3_ (922 mg, 6.68 mmol). The product was obtained as a yellowish solid with a 90% yield (1.05 g, 2.40 mmol). mp: 241. 8–242.9 °C. ^1^H NMR (400 MHz, DMSO-*d*_6_, δ in ppm): 9.79 (s, 1H, Int D_2_O), 8.29 (s, 1H), 8.21 (dd, *J* = 3.8, 1.2 Hz, 1H), 7.84 (dd, *J* = 5.0, 1.1 Hz, 1H), 7.68 (dd, *J* = 9.1, 5.1 Hz, 2H), 7.26 (dd, *J* = 5.0, 3.7 Hz, 1H), 7.19 (t, *J* = 8.9 Hz, 2H), 6.76 (s, 2H, Int D_2_O). ^13^C NMR (100 MHz, DMSO-*d*_6_, δ in ppm): 164.0, 161.0, 159.0 (d, *J* = 240.7 Hz), 152.7, 145.5, 142.5, 135.3, 132.3 (q, *J* = 33.4 Hz), 131.6, 129.5, 124.1, 123.1 (q, *J* = 274.3 Hz), 118.3, 115.5 (d, *J* = 22.2 Hz), 113.1, 101.3. IR (ATR-FTIR, cm^−1^): 3494 and 3337 (**v**_N-H_), 3414 (**v**_CON-H_), 3098 (**v**_Ar-H_), 1217 (**v**_C-F_), 1600 (**v**_NHC=O_). HRMS (APCI, [M + H]^+^, *m*/*z*) = 438.0289 (Calculated: 438.4386 for C_19_H_11_F_4_N_3_OS_2_).

#### 3.2.2. 3-Amino-*N*-(4-fluoro-2-nitrophenyl)-6-(thiophen-2-yl)-4-(trifluoromethyl)thieno[2,3-*b*]pyridine-2-carboxamide (**3**)

TPR (738 mg, 2.58 mmol), c3 (500 mg, 2.15 mmol), K_2_CO_3_ (743 mg, 5.37 mmol). The product was recrystallized using a toluene:DMF 4:1 mixture, as an orange solid, with a 92% yield (955 mg, 1.98 mmol). mp: 239.1–239.9 °C. ^1^H NMR (400 MHz, DMSO-*d*_6_, δ in ppm): 10.30 (s, 1H, Int D_2_O), 8.25 (s, 1H), 8.24 (dd, *J* = 3.8, 1.1 Hz, 1H), 7.99 (dd, *J* = 8.6, 3.0 Hz, 1H), 7.86 (dd, *J* = 5.0, 1.0 Hz, 1H), 7.84 (dd, *J* = 8.7, 5.2 Hz, 1H), 7.69 (ddd, *J* = 9.0, 7.8, 3.0 Hz, 1H), 7.27 (dd, *J* = 5.0, 3.8 Hz, 1H), 6.80 (s, 2H, Int D_2_O). ^13^C NMR (100 MHz, DMSO-*d*_6_, δ in ppm): 163.6, 160.8, 158.0 (d, *J* = 246.4 Hz), 152.8, 148.2, 146.0, 143.2 (d, *J* = 8.7 Hz), 142.0, 132.2 (d, *J* = 33.7 Hz), 131.5, 129.3 (d, *J* = 27.1 Hz), 128.43, 128.40 (d, *J* = 8.2 Hz), 126.7, 125.3 (d, *J* = 274.1 Hz), 124.0, 121.3 (d, *J* = 22.1 Hz), 117.6, 112.9, 112.4, 112.3 (d, *J* = 27.9 Hz), 112.2. IR (ATR-FTIR, cm^−1^): 3540 and 3351 (**v**_N-H_), 3524 (**v**_CON-H_), 3113 (**v**_Ar-H_), 1241 (**v**_C-F_), 1648 (**v**_NHC=O_), 1505 (**v**_Ar-NO2_). HRMS (APCI, [M + H]^+^, *m*/*z*) = 483.0170 (Calculated: 483.0209 for C_19_H_10_F_4_N_4_O_3_S_2_).

#### 3.2.3. 3-Amino-*N*-(4-chlorophenyl)-6-(thiophen-2-yl)-4-(trifluoromethyl)thieno[2,3-*b*]pyridine-2-carboxamide (**5**)

TPR (3.28 g, 11.46 mmol), c5 (1.80 g, 8.82 mmol), K_2_CO_3_ (3.05 g, 22.05 mmol). The product was obtained as a yellowish solid with a 95% yield (3.80 g, 8.38 mmol). mp: 237.1–237.8 °C. ^1^H NMR (400 MHz, DMSO-*d*_6_, δ in ppm): 9.86 (s, 1H, Int D_2_O), 8.30 (s, 1H), 8.22 (dd, *J* = 3.8, 1.2 Hz, 1H), 7.85 (dd, *J* = 5.0, 1.1 Hz, 1H), 7.72 (d, *J* = 8.9 Hz, 2H), 7.41 (d, *J* = 8.9 Hz, 2H), 7.26 (dd, *J* = 5.0, 3.8 Hz, 1H), 6.79 (s, 2H, Int D_2_O). ^13^C NMR (100 MHz, DMSO-*d*_6_, δ in ppm): 164.1, 161.0, 153.9, 145.8, 142.5, 137.9, 132.4 (d, *J* = 33.5 Hz), 131.7, 129.6, 129.5, 128.9, 128.1, 123.5, 123.1 (d, *J* = 274.1 Hz), 118.2, 113.2, 101.0. IR (ATR-FTIR, cm^−1^): 3508 and 3309 (**v**_N-H_), 3394 (**v**_CON-H_), 3073 (**v**_Ar-H_), 1236 (**v**_C-F_), 1641 (**v**_NHC=O_), 702 (**v**_C-Cl_). HRMS (APCI, [M + H]^+^, *m*/*z*) = 453.9976 (Calculated: 454.0062 for C_19_H_11_ClF_3_N_3_OS_2_).

#### 3.2.4. 3-Amino-*N*-(4-chloro-2-cyanophenyl)-6-(thiophen-2-yl)-4-(trifluoromethyl)thieno[2,3-*b*]pyridine-2-carboxamide (**6**)

TPR (693 mg, 2.42 mmol), c6 (504 mg, 2.20 mmol), K_2_CO_3_ (760 mg, 5.50 mmol). The product was recrystallized using a toluene:DMF 4:1 mixture, as a yellowish solid, with an 88% yield (920 mg, 1.95 mmol). mp: 264.8–266.5 °C. ^1^H NMR (400 MHz, DMSO-*d*_6_, δ in ppm): 10.24 (s, 1H, Int D_2_O), 8.32 (s, 1H), 8.24 (dd, *J* = 3.8, 1.2 Hz, 1H), 8.08 (d, *J* = 2.5 Hz, 1H), 7.87 (dd, *J* = 5.0, 1.1 Hz, 1H), 7.83 (dd, *J* = 8.7, 2.5 Hz, 1H), 7.61 (d, *J* = 8.8 Hz, 1H), 7.28 (dd, *J* = 5.0, 3.8 Hz, 1H) 6.84 (s, 2H, Int D_2_O). ^13^C NMR (100 MHz, DMSO-*d*_6_, δ in ppm): 163.9, 160.8, 152.8, 146.0, 142.0, 139.2, 133.9, 132.4, 132.0, 131.5, 130.2, 129.4, 129.2, 128.6, 122.6 (d, *J* = 274.3 Hz), 117.6, 115.8, 112.9, 111.2, 99.3. IR (ATR-FTIR, cm^−1^): 3523 and 3332 (**v**_N-H_), 3357 (**v**_CON-H_), 3090 (**v**_Ar-H_), 2222 (**v**_C__≡N_), 1651 (**v**_NHC=O_), 1235 (**v**_C-F_). HRMS (APCI, [M + H]^+^, *m*/*z*) = 478.9957 (Calculated: 479.0015 for C_20_H_10_ClF_3_N_4_OS_2_).

#### 3.2.5. 3-Amino-*N*-(4-chloro-2-nitrophenyl)-6-(thiophen-2-yl)-4-(trifluoromethyl)thieno[2,3-*b*]pyridine-2-carboxamide (**7**)

TPR (690 mg, 2.41 mmol), c7 (500 mg, 2.01 mmol), K_2_CO_3_ (694 mg, 5.02 mmol). The product was recrystallized using a toluene:DMF 4:1 mixture as an orange solid with an 85% yield (853 mg, 1.71 mmol). mp: 247.5–249.4 °C. ^1^H NMR (400 MHz, DMSO-*d*_6_, δ en ppm): 10.41 (s, 1H, Int D_2_O), 8.30 (s, 1H), 8.23 (d, *J* = 3.8 Hz, 1H), 8.09 (s, 1H), 7.95 (d, *J* = 8.8 Hz, 1H), 7.86 (d, *J* = 5.0 Hz, 1H), 7.80 (d, *J* = 7.8 Hz, 1H), 7.27 (dd, *J* = 5.0, 3.7 Hz, 1H), 6.81 (s, 2H, Int D_2_O). ^13^C NMR (100 MHz, DMSO-*d*_6_, δ en ppm): 163.9, 160.7, 142.7, 142.1, 133.6, 131.4, 129.2, 129.1, 128.9, 128.2, 127.3, 124.4, 124.0, 112.7, 109.6. IR (ATR-FTIR, cm^−1^): 3537 and 3326 (**v**_N-H_), 3093 (**v**_Ar-H_), 1643 (**v**_NHC=O_), 1494 and 1340 (**v**_C-NO2_), 1376 and 748 (**v**_C-F_). HRMS (APCI, [M + H]^+^, *m*/*z*) = 498.9859 (Calculated: 498.9913 for C_19_H_10_ClF_3_N_4_O_3_S_2_).

#### 3.2.6. 3-Amino-*N*-(4-bromophenyl)-6-(thiophen-2-yl)-4-(trifluoromethyl)thieno[2,3-*b*]pyridine-2-carboxamide (**9**)

TPR (641 mg, 2.24 mmol), c9 (506 mg, 2.04 mmol), K_2_CO_3_ (704 mg, 5.09 mmol). The product was obtained as a yellow solid with a 94% yield (1.08 g, 2.17 mmol). mp: 241.0–242.1 °C. ^1^H NMR (400 MHz, DMSO-*d*_6_, δ in ppm): 9.85 (s, 1H, Int D_2_O), 8.29 (s, 1H), 8.22 (dd, *J* = 3.8, 1.2 Hz, 1H), 7.85 (dd, *J* = 5.0, 1.1 Hz, 1H), 7.67 (d, *J* = 8.9 Hz, 2H), 7.53 (d, *J* = 8.9 Hz, 2H), 7.26 (dd, *J* = 5.0, 3.8 Hz, 1H), 6.79 (s, 2H, Int D_2_O). ^13^C NMR (100 MHz, DMSO-*d*_6_, δ in ppm): 164.1, 162.7, 161.0, 152.8, 145.6, 142.5, 138.6, 132.4 (d, *J* = 33.7 Hz), 131.7, 129.6, 123.9, 123.1 (d, *J* = 274.1 Hz), 118.2, 116.0, 113.2. IR (ATR-FTIR, cm^−1^): 3503 and 3311 (**v**_N-H_), 3402 (**v**_CON-H_), 3076 (**v**_Ar-H_), 1236 (**v**_C-F_), 1644 (**v**_NHC=O_). HRMS (APCI, [M + H]^+^, *m*/*z*) = 497.9492 (Calculated: 497.9551 for C_19_H_11_BrF_3_N_3_OS_2_)

#### 3.2.7. 3-Amino-*N*-(4-bromo-2-cyanophenyl)-6-(thiophen-2-yl)-4-(trifluoromethyl)thieno[2,3-*b*]pyridine-2-carboxamide (**11**)

TPR (628 mg, 2.19 mmol), c11 (500 mg, 1.83 mmol), K_2_CO_3_ (632 mg, 4.57 mmol). The product was recrystallized using a toluene:DMF 4:1 mixture as a yellowish solid, with a 90% yield (860 mg, 1.65 mmol). mp: 254.8–256.6 °C. ^1^H NMR (400 MHz, DMSO-*d*_6_, δ in ppm): 10.24 (s, 1H, Int D_2_O), 8.30 (s, 1H), 8.23 (dd, *J* = 3.7, 0.9 Hz, 1H), 8.13 (s, 1H), 7.90 (d, *J* = 8.5 Hz, 1H), 7.85 (dd, *J* = 5.0, 1.1 Hz, 1H), 7.64 (s, 1H), 7.27 (dd, *J* = 5.1, 3.8 Hz, 1H), 6.83 (s, 2H, Int D_2_O). ^13^C NMR (100 MHz, DMSO-*d*_6_, δ in ppm): 164.1, 160.8, 155.2, 152.5, 142.1, 141.6, 136.6, 135.0, 132.0 (d, *J* = 31.4 Hz), 131.3, 131.0, 129.4, 129.2, 129.1, 128.4, 122.7 (d, *J* = 273.7 Hz), 117.8, 116.0, 112.7, 111.1. IR (ATR-FTIR, cm^−1^): 3530 and 3357 (**v**_N-H_), 3377 (**v**_CON-H_), 3102 (**v**_Ar-H_), 2233 (**v**_C__≡N_), 1649 (**v**_NHC=O_), 1294 (**v**_C-F_). HRMS (APCI, [M + H]^+^, *m*/*z*) = 522.9440 (Calculated: 522.9504 for C_20_H_10_BrF_3_N_4_OS_2_).

#### 3.2.8. 3-Amino-*N*-(4-bromo-2-nitrophenyl)-6-(thiophen-2-yl)-4-(trifluoromethyl)thieno[2,3-*b*]pyridine-2-carboxamide (**12**)

TPR (585 mg, 2.04 mmol), c12 (500 mg, 1.70 mmol), K_2_CO_3_ (589 mg, 4.26 mmol). The product was recrystallized using a toluene:DMF 4:1 mixture as an orange solid with an 88% yield (812 mg, 1.50 mmol). mp: 253.0–253.6 °C. ^1^H NMR (400 MHz, DMSO-*d*_6_, δ in ppm): 10.40 (s, 1H, Int D_2_O), 8.32 (s, 1H), 8.25 (dd, *J* = 3.8, 1.1 Hz, 1H), 8.22 (s, 1H), 7.95 (d, *J* = 8.8 Hz, 1H), 7.87 (dd, *J* = 5.0, 1.1 Hz, 1H), 7.83 (s, 1H), 7.28 (dd, *J* = 5.0, 3.8 Hz, 1H), 6.83 (s, 2H, Int D_2_O). ^13^C NMR (100 MHz, DMSO-*d*_6_, δ in ppm): 163.7, 160.8, 152.8, 142.9, 142.1, 136.7, 131.5, 129.4, 129.2, 127.5, 127.3, 122.6 (d, *J* = 274.1 Hz), 117.6, 112.8. IR (ATR-FTIR, cm^−1^): 3525 and 3332 (**v**_N-H_), 3347 (**v**_CON-H_), 3092 (**v**_Ar-H_), 1649 (**v**_NHC=O_), 1476 and 1336 (**v**_C-F_). HRMS (APCI, [M + H]^+^, *m*/*z*) = 542.9348 (Calculated: 542.9408 for C_19_H_10_BrF_3_N_4_O_3_S_2_).

#### 3.2.9. 3-Amino-*N*-(4-bromo-2-(trifluoromethyl)phenyl)-6-(thiophen-2-yl)-4-(trifluoromethyl)thieno[2,3-*b*]pyridine-2-carboxamide (**13**)

TPR (511 mg, 1.79 mmol), c13 (514 mg, 1.62 mmol), K_2_CO_3_ (561 mg, 4.06 mmol). The product was recrystallized using a toluene:DMF 4:1 mixture. The product was obtained as a yellowish solid with a 92% yield (843 mg, 1.49 mmol). mp: 260.8–261.5 °C. ^1^H NMR (400 MHz, DMSO-*d*_6_, δ in ppm): 9.74 (s, 1H, IntD_2_O), 8.23 (dd, *J* = 3.8, 1.1 Hz, 1H), 7.99 (d, *J* = 2.3 Hz, 1H), 7.96 (dd, *J* = 8.5, 2.4 Hz, 1H), 7.86 (dd, *J* = 5.0, 1.1 Hz, 1H), 7.55 (d, *J* = 8.4 Hz, 1H), 7.27 (dd, *J* = 5.0, 3.8 Hz, 1H), 6.72 (s, 2H, IntD_2_O). ^13^C NMR (100 MHz, DMSO-*d*_6_, δ in ppm): 164.5, 160.6, 152.5, 145.2, 142.1, 136.1, 135.0, 133.3, 132.3, 131.9, 131.3, 129.3, 129.3, 129.2, 129.1, 122.6 (d, *J* = 274.5 Hz), 119.8, 117.8, 112.7, 109.5. IR (ATR-FTIR, cm^−1^): 3521 and 3325 (**v**_N-H_), 3441 (**v**_CON-H_), 3071 (**v**_Ar-H_), 1647 (**v**_NHC=O_), 1240 (**v**_C-F_). HRMS (APCI, [M + H]^+^, *m*/*z*) = 565.9353 (Calculated: 565.9431 for C_20_H_10_BrF_6_N_3_OS_2_).

#### 3.2.10. 3-Amino-*N*-(4-iodophenyl)-6-(thiophen-2-yl)-4-(trifluoromethyl)-thieno[2,3-*b*]pyridine-2-carboxamide (**14**)

TPR (301 mg, 1.05 mmol), c14 (239 mg, 0.80 mmol), K_2_CO_3_ (279 mg, 2.02 mmol). The product was recrystallized using a toluene:DMF 4:1 mixture as a yellowish solid with a 95% yield (415 mg, 0.76 mmol). mp: 236.5–237.6 °C. ^1^H NMR (400 MHz, DMSO-*d*_6_, δ in ppm): 9.79 (s, 1H, Int D_2_O), 8.27 (s, 1H) 8.20 (d, *J* = 3.8 Hz, 1H), 7.83 (d, *J* = 5.0 Hz, 1H), 7.67 (d, *J* = 8.6 Hz, 2H), 7.53 (d, *J* = 8.8 Hz, 2H), 7.25 (dd, *J* = 5.0, 3.7 Hz, 1H), 6.79 (s, 2H, Int D_2_O). ^13^C NMR (100 MHz, DMSO-*d*_6_, δ in ppm): 154.5, 151.5, 146.1, 144.2, 133.4, 132.1, 130.1, 129.4, 129.3, 128.1, 126.0, 125.4, 121.9, 119.0, 116.6, 112.8, 104.7. IR (ATR-FTIR, cm^−1^): 3497 and 3313 (**v**_N-H_), 3411 (**v**_CON-H_), 3101–3050 (**v**_Ar-H_), 1236 (**v**_C-F_), 1647 (**v**_NHC=O_), 586–524 (**v**_C-I_). HRMS (APCI, [M + H]^+^, *m*/*z*) = 545.9387 (Calculated: 545.9419 for C_19_H_11_F_3_IN_3_OS_2_).

#### 3.2.11. 3-Amino-*N*-(4-iodo-2-methylphenyl)-6-(thiophen-2-yl)-4-(trifluoromethyl)thieno[2,3-*b*]pyridine-2-carboxamide (**15**)

TPR (643 mg, 2.25 mmol), c15 (536 mg, 1.73 mmol), K_2_CO_3_ (597 mg, 4.33 mmol). The product was obtained as a yellowish solid with a 92% yield (891 mg, 1.59 mmol). mp: 276.5–277.4 °C. ^1^H NMR (400 MHz, DMSO-*d*_6_, δ in ppm): 9.53 (s, 1H, Int D_2_O), 8.23 (s, 1H), 8.22 (dd, *J* = 3.8, 1.2 Hz, 1H), 7.85 (dd, *J* = 5.0, 1.1 Hz, 1H), 7.68 (d, *J* = 1.3 Hz, 1H), 7.57 (dd, *J* = 8.3, 1.5 Hz, 1H), 7.27 (dd, *J* = 5.0, 3.8 Hz, 1H), 7.15 (d, *J* = 8.3 Hz, 1H), 6.70 (s, 2H, Int D_2_O), 2.20 (s, 3H). ^13^C NMR (100 MHz, DMSO-*d*_6_, δ in ppm): 163.6, 160.6, 152.3, 144.9, 142.1, 138.7, 137.1, 136.1, 134.9, 132.0 (d, *J* = 33.6 Hz), 131.3, 129.2, 122.7 (d, *J* = 274.5 Hz), 118.0, 112.7, 112.7, 101.1, 91.5, 17.5. IR (ATR-FTIR, cm^−1^): 3533 and 3349 (**v**_N-H_), 3420 (**v**_CON-H_), 3077 (**v**_Ar-H_), 2978 (**v**_C-H_) 1125 (**v**_C-F_), 1598 (**v**_NHC=O_), 696 and 495 (**v**_C-I_). HRMS (APCI, [M + H]^+^, *m*/*z*) = 559.9473 (Calculated: 559.9575 for C_20_H_13_F_3_IN_3_OS_2_).

#### 3.2.12. 3-Amino-*N*-(2-cyano-4-iodophenyl)-6-(thiophen-2-yl)-4-(trifluoromethyl)thieno[2,3-*b*]pyridine-2-carboxamide (**16**)

TPR (819 mg, 2.90 mmol), c16 (833 mg, 2.60 mmol), K_2_CO_3_ (898 mg, 6.5 mmol). The product was recrystallized using a toluene:DMF 4:1 mixture as a yellowish solid with a 91% yield (1.35 g, 2.37 mmol). mp: 260.8–261.5 °C. ^1^H NMR (400 MHz, DMSO-*d*_6_, δ in ppm): 10.22 (s, 1H, Int D_2_O), 8.29 (s, 1H), 8.22 (dd, *J* = 3.8, 1.1 Hz, 1H), 8.18 (s, 1H), 8.00 (d, *J* = 8.5 Hz, 1H), 7.85 (dd, *J* = 5.0, 1.1 Hz, 1H), 7.54 (s, 1H), 7.26 (dd, *J* = 5.1, 3.7 Hz, 1H), 6.82 (s, 2H, Int D_2_O). ^13^C NMR (100 MHz, DMSO-*d*_6_, δ in ppm): 164.3, 160.8, 152.3, 142.2, 140.5, 131.9 (d, *J* = 33.3 Hz), 131.3, 129.1, 122.7 (q, *J* = 273.8 Hz), 118.6, 118.0, 116.1, 112.7, 110.9. IR (ATR-FTIR, cm^−1^): 3528 and 3356 (**v**_N-H_), 3099 (**v**_Ar-H_), 2231 (**v**_C__≡N_),1122 (**v**_C-F_), 1649 (**v**_NHC=O_), 733 and 440 (**v**_C-I_). HRMS (APCI, [M + H]^+^, *m*/*z*) = 570.9308 (Calculated: 570.9371 for C_20_H_10_F_3_IN_4_OS_2_).

#### 3.2.13. 3-Amino-*N*-(4-iodo-2-nitrophenyl)-6-(thiophen-2-yl)-4-(trifluoromethyl)thieno[2,3-*b*]pyridine-2-carboxamide (**17**)

TPR (670 mg, 2.30 mmol), c17 (612 mg, 1.80 mmol), K_2_CO_3_ (621 mg, 4.50 mmol). The product was recrystallized using a toluene:DMF 5:1 mixture as an orange solid with an 86% yield (913 mg, 1.55 mmol). mp: 260.0–261.0 °C. ^1^H NMR (400 MHz, DMSO-*d*_6_, δ in ppm): 10.36 (s, 1H, Int D_2_O), 8.32 (s, 1H), 8.24 (s, 1H), 8.09 (d, *J* = 8.2 Hz, 1H), 7.87 (d, *J* = 5.6 Hz, 1H), 7.69 (d, *J* = 8.3 Hz, 1H), 7.27 (s, 1H), 7.17 (d, *J* = 6.9 Hz, 1H), 6.83 (s, 2H, Int D_2_O). ^13^C NMR (100 MHz, DMSO-*d*_6_, δ in ppm): 163.4, 160.8, 153.0, 146.2, 142.6, 142.0, 132.9, 131.6, 129.6, 129.2, 129.0, 128.3, 127.4, 125.4, 121.3, 117.6, 113.0. IR (ATR-FTIR, cm^−1^): 3527 and 3322 (**v**_N-H_), 3107 (**v**_Ar-H_), 1153 (**v**_C-F_), 1475 (**v**_C-NO2_), 1645 (**v**_NHC=O_), 747 and 440 (**v**_C-I_). HRMS (APCI, [M + H]^+^, *m*/*z*) = 590.9197 (Calculated: 590.9264 for C_19_H_10_F_3_IN_4_O_3_S_2_).

#### 3.2.14. 3-Amino-*N*-(4-iodo-2-(trifluoromethyl)phenyl)-6-(thiophen-2-yl)-4-(trifluoromethyl)thieno[2,3-*b*]pyridine-2-carboxamide (**18**)

TPR (659 mg, 2.30 mmol), c18 (654 mg, 1.80 mmol), K_2_CO_3_ (622 mg, 4.5 mmol). The product was recrystallized using a toluene:DMF 5:1 mixture as a yellowish solid with a 92% yield (1.02 g, 1.66 mmol). mp: 260.8–261.5 °C. ^1^H NMR (400 MHz, DMSO-*d*_6_) δ: 9.73 (s, 1H, IntD_2_O), 8.27 (s, 1H), 8.20 (d, *J* = 3.78 Hz, 1H), 8.02 (s, 1H), 7.83 (d, *J* = 5.00 Hz, 1H), 7.52 (s, 1H), 7.26 (t, *J* = 4.5 Hz, 1H), 6.70 (s, 2H, IntD_2_O). ^13^C NMR (100 MHz, DMSO-*d*_6_, δ en ppm): 164.7, 160.7, 152.0, 144.0, 142.3, 141.6, 134.6 (d, *J* = 5.1 Hz), 132.3, 131.9 (d, *J* = 32.9 Hz), 131.1, 129.0, 127.6, 122.7 (q, *J* = 274.2 Hz), 118.2, 112.5 (d, *J* = 6.1 Hz), 78.3. IR (ATR-FTIR, cm^−1^): 3504 and 3337 (**v**_N-H_), 3439 (**v**_CON-H_), 3068 (**v**_Ar-H_), 1300 (**v**_C-F_), 1655 (**v**_NHC=O_), 463 (**v**_C-I_). HRMS (APCI, [M + H]^+^, *m*/*z*) = 613.9202 (Calculated: 613.9292 for C_20_H_10_F_6_IN_3_OS_2_).

### 3.3. General Method for the Synthesis of the Final Derivatives (**FDI-6** and, **1**–**18**): Microwave Heating

In a 5 mL microwave reaction flask with a magnetic stirrer, the appropriate 2-chloro-*N*-phenylacetamide (**cF**, **c1**–**c18**) (1 equiv.), 6-(thiophen-2-yl)-2-thioxo-4-(trifluoromethyl)-1,2-dihydropyridine-3-carbonitrile (**TPR**) (1.2 equiv.), and K_2_CO_3_ (2.5 equiv.) were added, using EtOH as the solvent. The flask was placed in the microwave, and a 3 h reaction program was run, with the temperature and absorption set at 90 °C and normal level, respectively. Once the reaction was completed, a precipitate was observed in the flask, which was then filtered off under vacuum, washed with water (5 mL) and cold absolute EtOH (3 × 2 mL), and allowed to dry under vacuum for a period of 12 h.

If necessary, the sought product was dissolved in EtOAc, adsorbed in silica gel, and purified using flash chromatography with a hexane:ethyl acetate gradient. The solvent from the collected fractions containing the compound of interest was distilled under reduced pressure, and the resultant solid product was vacuum-dried for 12 h.

#### 3.3.1. 3-Amino-*N*-(4-fluoro-2-methylphenyl)-6-(thiophen-2-yl)-4-(trifluoromethyl)thieno[2,3-*b*]pyridine-2-carboxamide (**1**)

TPR (100 mg, 0.35 mmol), c1 (54 mg, 0.29 mmol), K_2_CO_3_ (92 mg, 0.67 mmol). The product was obtained as an orange solid with a 91% yield (119 mg, 0.26 mmol). mp: 234–236 °C. ^1^H NMR (600 MHz, DMSO-*d*_6_, δ in ppm): 8.23 (s, 1H), 8.16 (dd, *J* = 3.6, 0.6 Hz, 1H), 7.81 (dd, *J* = 5.0, 1.1 Hz, 1H), 7.58 (s, 1H), 7.25 (dd, *J* = 5.0, 3.7 Hz, 1H), 7.05 (dd, *J* = 9.6, 2.7 Hz, 1H), 6.94 (td, *J* = 8.6, 3.2 Hz, 1H). ^13^C NMR (150 MHz, DMSO-*d*_6_, δ in ppm): 164.0, 160.4, 150.2, 142.8, 130.2, 129.0, 127.9, 123.0 (d, *J* = 274.4 Hz), 115.8, 111.7, 18.8. MS ([M − H]^−^, *m*/*z*): 450.0 (Calculated: 450.0 for for C_20_H_13_F_4_N_3_OS_2_).

#### 3.3.2. 3-Amino-*N*-(2-cyano-4-fluorophenyl)-6-(thiophen-2-yl)-4-(trifluoromethyl)thieno[2,3-*b*]pyridine-2-carboxamide (**2**)

TPR (100 mg, 0.35 mmol), c2 (57 mg, 0.29 mmol), K_2_CO_3_ (93 mg, 0.67 mmol). The product was obtained as a yellow solid with a 92% yield (123 mg, 0.27 mmol). mp: 234–236 °C. ^1^H NMR (600 MHz, DMSO-*d*_6_, δ in ppm): 10.17 (s, 1H, Int D_2_O), 8.30 (s, 1H), 8.22 (dd, *J* = 3.8, 1.1 Hz, 1H), 7.92 (dd, *J* = 8.4, 3.0 Hz, 1H), 7.85 (dd, *J* = 5.0, 1.1 Hz, 1H), 7.65 (td, *J* = 8.6, 3.0 Hz, 1H), 7.61 (dd, *J* = 9.0, 5.1 Hz, 1H), 7.26 (dd, *J* = 5.0, 3.7 Hz, 1H), 6.83 (s, 2H, Int D_2_O). ^13^C NMR (150 MHz, DMSO-*d*_6_, δ in ppm): 164.5, 161.3, 159.6 (d, *J* = 245.3 Hz), 153.2, 146.3, 142.5, 137.2 (d, *J* = 3.1 Hz), 132.6 (q, *J* = 33.5 Hz), 130.1 (d, *J* = 8.8 Hz), 132.1, 129.7 (d, *J* = 26.6 Hz), 129.1, 125.8, 123.1 (d, *J* = 274.3 Hz), 121.7 (d, *J* = 22.4 Hz), 120.1 (d, *J* = 26.2 Hz), 118.1, 116.3 (d, *J* = 2.6 Hz), 113.2 (d, *J* = 6.3 Hz), 111.9 (d, *J* = 10.3 Hz), 99.9. MS ([M − H]^−^, *m*/*z*): 461.0 (Calculated: 461.0 for for C_20_H_10_F_4_N_4_OS_2_).

#### 3.3.3. 3-Amino-*N*-(4-fluor-2-(trifluoromethyl)phenyl)-6-(thiophen-2-yl)-4-(trifluoromethyl)thieno[2,3-*b*]pyridine-2-carboxamide (**4**)

TPR (100 mg, 0.35 mmol), c4 (69 mg, 0.29 mmol), K_2_CO_3_ (92 mg, 0.67 mmol). The product was obtained as a yellowish solid with a 93% yield (136 mg, 0.27 mmol). mp: 197–198 °C. ^1^H NMR (600 MHz, DMSO-*d*_6_, δ in ppm): 9.74 (s, 1H, Int D_2_O), 8.24 (s, 1H), 8.17 (s, 1H), 7.95 (s, 1H), 7.81 (d, *J* = 4.1 Hz, 1H), 7.52 (s, 1H), 7.45 (s, 1H), 7.25 (t, *J* = 4.5 Hz, 1H), 6.68 (s, 2H, Int D_2_O). ^13^C NMR (150 MHz, DMSO-*d*_6_, δ in ppm): 165.1, 160.7, 151.5, 143.0, 142.5, 131.7 (d, *J* = 32.0 Hz), 130.9, 129.2, 128.7, 126.3, 123.3 (d, *J* = 274.3 Hz), 122.9 (q, *J* = 274.1, 273.7 Hz), 119.6 (d, *J* = 22.8 Hz), 118.7, 113.5 (d, *J* = 23.1 Hz), 112.3. MS ([M − H]^−^, *m*/*z*): 504.0 (Calculated: 504.0 for C_20_H_10_F_7_N_3_OS_2_).

#### 3.3.4. 3-Amino-*N*-(4-chloro-2-(trifluoromethyl)phenyl)-6-(thiophen-2-yl)-4-(trifluoromethyl)thieno[2,3-*b*]pyridine-2-carboxamide (**8**)

TPR (100 mg, 0.35 mmol), c8 (79 mg, 0.29 mmol), K_2_CO_3_ (101 mg, 0.73 mmol). The product was obtained as an orange solid with a 90% yield (136 mg, 0.26 mmol). mp: 222–223 °C. ^1^H NMR (600 MHz, DMSO-*d*_6_, δ in ppm): 8.51 (d, *J* = 8.9 Hz, 1H), 8.13 (s, 1H), 8.08 (d, *J* = 3.8, 1H), 7.75 (dd, *J* = 5.0, 1.1 Hz, 1H), 7.38 (s, 1H), 7.30 (dd, *J* = 8.5, 2.6 Hz, 1H), 7.22 (dd, *J* = 5.0, 3.7 Hz, 1H), 6.61 (s, 2H, Int D_2_O). ^13^C NMR (150 MHz, DMSO-*d*_6_, δ in ppm): 166.2, 160.6, 150.6, 143.1, 137.2, 131.1, 130.2 (d, *J* = 32.2 Hz), 129.6, 128.8, 127.3, 126.0, 125.5, 124.8, 123.7, 123.2 (d, *J* = 273.7 Hz), 121.0, 120.4, 119.6, 111.0. MS ([M − H]^−^, *m*/*z*): 519.9 (Calculated: 520.0 for C_20_H_10_ClF_6_N_3_OS_2_).

#### 3.3.5. 3-Amino-*N*-(4-bromo-2-methylphenyl)-6-(thiophen-2-yl)-4-(trifluoromethyl)thieno[2,3-*b*]pyridine-2-carboxamide (**10**)

TPR (100 mg, 0.34 mmol), **c10** (76 mg, 0.29 mmol), K_2_CO_3_ (101 mg, 0.73 mmol). The product was obtained as an orange solid with a 94% yield (139 mg, 0.27 mmol). mp: >255 °C. ^1^H NMR (600 MHz, DMSO-*d*_6_, δ in ppm): 8.26 (s, 1H), 8.19 (d, *J* = 3.6, 1H), 7.82 (d, *J* = 5.0, Hz, 1H), 7.44 (s,1H), 7.47 (s, 1H), 7.33 (d, *J* = 8.4 Hz, 1H), 7.26 (dd, *J* = 5.0, 3.7 Hz, 1H), 2.23 (s, 3H). ^13^C NMR (150 MHz, DMSO-*d*_6_, δ in ppm): 163.9, 160.5, 151.2, 142.7, 142.4, 136.1, 132.3, 131.4 (d, *J* = 33.9 Hz), 130.7, 129.0, 128.5, 127.8, 122.8 (q, *J* = 273.9 Hz), 118.7, 116.0, 112.2, 18.1. MS ([M − H]^−^, *m*/*z*): 509.8 (Calculated: 510.0 for C_20_H_13_BrF_3_N_3_OS_2_).

### 3.4. Western Blotting

We used a FOXM1 mouse monoclonal antibody (Santa Cruz Biotechnology, Dallas, TX, USA) and IRDye^®^ 800CW Goat anti-Mouse IgM (Li-Cor Biosciences, Lincoln, NE, USA). The cells were cultured in RPMI medium (Gibco™) supplemented with 10% fetal bovine serum (FBS) in a 5% CO_2_ atmosphere at 37 °C.

MDA-MB-231 cells were seeded at a density of 2 × 10^5^ cells per well in 6-well plates. After treatment with **FDI-6** and **1**–**18** at 40 µM for 24 h, the cells were lysed with RIPA lysis and extraction buffer (Thermo Fisher, Waltham, MA, USA) according to the manufacturer’s protocol to yield the whole-cell extracts, which were then centrifuged at 14,000 rpm to remove any cell debris.

The protein levels in the supernatant were measured using the Bradford assay (Bio-Rad Protein Assay Die, Hercules, CA, USA); then, the protein (40 μg/lane) was loaded into a 4–15% SDS-PAGE gel (sodium dodecyl sulfate polyacrylamide gel electrophoresis gel, Bio-Rad Mini-Protean TGX Gels, Hercules, CA, USA). After completing the run, the protein was transferred from the gel to a nitrocellulose membrane (Bio-Rad Nitrocellulose Membranes, 0.45 μm, Hercules, CA, USA). The membrane was incubated with the primary antibody (1:1000 dilution) at 4 °C overnight.

TBST was prepared using Tween 20 (Sigma-Aldrich, TWEEN^®^ 20 for molecular biology, viscous liquid) and a 10 × TBS solution previously prepared using UltraPure™ Tris Buffer (Invitrogen, powder format, Waltham, MA, USA), NaCl (Sigma-Aldrich, Darmstadt, Germany), and KCl (Sigma-Aldrich, Darmstadt, Germany); HCl (Sigma-Aldrich, ACS Reagent, 37%, Darmstadt, Germany) was used to adjust the pH to 7.8.

Then, the membrane was washed three times with TBST, incubated with the corresponding Li-Cor secondary antibody, and incubated again at room temperature for 1 h. The membrane was washed three times (15 min total) with TBST. The blots were visualized using an Odyssey scanner (LI-COR Biosciences, Lincoln, NE, USA). β-actin (β-actin mouse monoclonal IgG) was used to normalize the FOXM1 levels after treatment. The quantification was carried out for all proteins relative to β-actin using ImageJ for each lane. We analyzed the data using GraphPad Prism 4.0. The average values in all the experiments were calculated after three independent experiments (*n* = 3) and are expressed as means ± SEMs; *p* values were calculated by one way ANOVA (** = *p* ≤ 0.01; *** = *p* ≤ 0.001).

### 3.5. Cell-Proliferation-Inhibition (MTT) Assay

MDA-MB-231 cells were cultured in RPMI medium (Gibco^TM^, Waltham, MA, USA), supplemented with 10% fetal bovine serum (FBS) in a 5% CO_2_ atmosphere at 37 °C. The cells were seeded in 96-well plates (approx. 2000 cells/well); then treated with **FDI-6**, **1**, **6**, and **16** at different concentrations (100, 25, 6.25, 1.56, 0.39, and 0.098 μM); and incubated for 72 h. After 72 h, we added 30 μL of MTT solution (3 mg/mL) and continued the incubation for 3 h at 37 °C (humidified incubator; 5% CO_2_). The precipitated crystals were dissolved using DMSO, and the absorbance of the resulting solution was recorded at 570 nm using a Synergy H1-Hybrid Multi-Mode Reader (BioTek, Winooski, VT, USA). We analyzed the data using GraphPad Prism. All the experiments were carried out in biological and experimental triplicates. The data are expressed as means (*n* = 9) ± SEMs.

### 3.6. Molecular Docking

The preparation of the protein and ligands, as well as the molecular-docking calculations, were carried out in the different modules available in the Molecular Operating Environment (MOE) software [20].

The crystal structure of FOXM1 bound to the DNA-binding domain was retrieved from the Protein Data Bank (PDB_ID: 3G73) with a resolution of 2.21 Å [25]. Using the LigX preparation module, FOXM1’s chain A, DNA-binding domain, and water molecules were removed; simultaneously, the correct protonation states were assigned using Protonate3D. The energy of the protein was minimized using the Energy Minimize module with the AMBER99 forcefield. The compounds (**FDI-6** and **1**–**18**) were drawn in MOE and were also prepared using the LigX and Energy Minimize tools with the MMFF94x forcefield.

The molecular docking was carried out with the Dock module using the following parameters. The key residues His287, Arg297, Val305, Lys260, Arg286, and Trp308, which we identified in our previous work [6], were used to define the docking binding site. The selected placement methodology was “Triangle Matcher”, which is considered the best method for standard and well-defined binding sites [21]. Thirty complexes were allowed to be generated for each tested ligand. Duplicate complexes were then removed: poses were considered as duplicates if the same set of ligand–receptor atom pairs was involved in hydrogen-bond interactions and the same set of ligand-atom-receptor residue pairs were involved in hydrophobic interactions. The accepted poses were scored according to the London dG scoring function. All the saved solutions were subjected to a further refinement step, based on the molecular mechanics MMFF94x field. All the receptor atoms were held fixed during the refinement. The docking score (S) was evaluated using the GBVI/WSA dG scoring function with the Generalized Born solvation model (GBVI). The GBVI/WSA dG is a forcefield-based scoring function, which has been trained using the MMFF94x and AMBER99 forcefields on the 99 protein–ligand complexes of the Solvated Interaction Energy (SIE) training set [26]. A maximum of five conformations were allowed to be saved for each ligand.

### 3.7. MEP Maps

First, we carried out a conformational analysis of all the **FDI-6** derivatives (**1**–**18**) and **FDI-6**, and used the minimum energy conformer to perform a geometry optimization using the PM6 semiempirical approach [27] (the calculated IR spectra showed that all the frequencies were positive, meaning that the molecules were in a minimum on the potential energy surface). Then, we carried out a geometry re-optimization, for a more precise determination of the energy value and electronic-density characteristics, with density functional level theory (DFT) with the hybrid functional of Becke, Lyn, Yang, and Parr (B3LYP) and the 6–31*G basis set [28]. By using the DFT re-optimized structures, we calculated the molecular electrostatic potential (MEP) mapped onto an iso-density surface (0.002 e^−^/Å^3^), and the polar surface area (PSA) for **FDI-6** and derivatives **1**–**18** [23,24]. All these calculations were performed by using SPARTAN 18 [29].

## 4. Conclusions

We prepared 18 thieno[2,3-*b*]pyridine derivatives of the **FDI-6** compound that have the ring of the phenylacetamide moiety substituted at positions 2 and 4, with good yields, through a Thorpe–Ziegler-type isomerization. The evaluation of the FOXM1-inhibitory activity for all the compounds showed that the -CN substituted compounds, especially **6** and **16**, exerted significant FOXM1-inhibitory activity. The correlation between the results for FOXM1 inhibition and the cell anti-proliferative-activity assay suggests that the cytotoxic activity of the compounds might be related to their interaction with FOXM1 inhibition. On the other hand, molecular-docking calculations showed that the S scores for compounds **6** and **16** were more favorable than those of the parent compounds, regardless of the halogen. The MEP maps show that the presence of a -CN moiety favors the formation of a high-electron-density region over the -CN and the adjacent carbonyl that affects the overall electron density of the phenylacetamide ring, which may be related to its interaction with the DBD site and, hence, with the FOXM1-inhibitory activity. Together with the synthesis, the experimental results for FOXM1 inhibition, the molecular docking, and the MEP map calculations provide information that will help us to design more potent FOXM1 inhibitors.

## Figures and Tables

**Table 1:** IC_50_ values determined for test molecules **FDI-6**, **1**, **6**, and **16** using the human breast cancer cell line MDA-MB-231.

Compound	MDA-MB-231 IC_50_ (μM) ^a^
**FDI-6**	11.67 ± 1.35
**1**	171.5 ± 1.49
**6**	8.75 ± 1.27
**16**	4.61 ± 1.34

**^a^** These values were calculated after a 72 h incubation period with the drug molecules; all values represent the mean (*n* = 9) ± SEM.

**Table 2:** Docking scores calculated for the FDI derivatives during the MOE docking protocol.

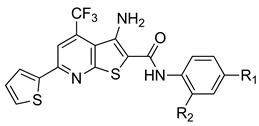			
Compound	S (kcal/mol) ^a^	R_1_	R_2_
**FDI-6**	−5.3383	-F	-H
**1**	−5.6402	-F	-CH_3_
**2**	−5.4899	-F	-CN
**3**	−5.9180	-F	-NO_2_
**4**	−5.4391	-F	-CF_3_
**5**	−5.5565	-Cl	-H
**6**	−7.2807	-Cl	-CN
**7**	−6.1224	-Cl	-NO_2_
**8**	−5.5823	-Cl	-CF_3_
**9**	−5.6117	-Br	-H
**10**	−5.8423	-Br	-CH_3_
**11**	−5.9348	-Br	-CN
**12**	−6.1845	-Br	-NO_2_
**13**	−5.7709	-Br	-CF_3_
**14**	−5.6386	-I	-H
**15**	−5.5661	-I	-CH_3_
**16**	−5.8317	-I	-CN
**17**	−5.7237	-I	-NO_2_
**18**	−5.7932	-I	-CF_3_

**^a^** S corresponds to the score of the last stage, in this case, calculated under the Generalized-Born Volume Integral/Weighted Surface area scoring function (GBVI/WSA dG) in kcal/mol.

**Table 3:** Summary of MEP values of selected thieno[2,3-*b*]pyridines.

MEP Values (kJ/mol) ^a^			
	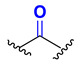	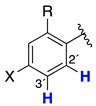	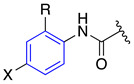
**FDI-6**	−112.0	44.0	−50.0 to 17.4
**5**	−117.4	83.4	−65.0 to 4.1
**9**	−111.4	81.4	−65.0 to 0.4
**14**	−109.0	77.4	−69.7 to −14.7
**2**	−128.7	108.2	−5.0 to 37.5
**6**	−154.1	110.0	−7.0 to 50.0
**11**	−165.0	98.8	−18.1 to 51.3
**16**	−160.0	108.8	−23.2 to 54.7
**1**	−167.6	75.3	−104 to 59
**7**	−151.0	119.3	−7.0 to 30.4
**1**	−157.1	76.4	−72.0 to 3.0
**17**	−146.3	112.0	−36.4 to 58.1

**^a^** The average MEP values correspond to those parts of the molecule (top row) depicted in blue that showed different MEP values from FDI-6 and parent molecules. For a more detailed list of values, please see Table S5.

## Data Availability

Data sharing contains in this article and Supplementary Martials.

## Supplementary Materials

The following supporting information can be downloaded at https://www.mdpi.com/article/10.3390/ph15030283/s1. General methods for the synthesis of the 2-chloro-*N*-phenylacetamides (**cF**, **c1**–**c18**); Procedure for the synthesis of 6-(Thiophen-2-yl)-2-thioxo-4-(trifluoromethyl)-1,2-dihydropyridine-3-carbonitrile (**TPR**); Spectra of compounds **FDI-6** and **1**–**18**; Western blot images; Cell viability detailed results; Molecular-docking complete energy results. Reference [30] is cited in the supplementary materials.
