# Supplementary material for: Structure–Activity Relationship of N-Phenylthieno[2,3-b]pyridine-2-carboxamide Derivatives Designed as Forkhead Box M1 Inhibitors: The Effect of Electron-Withdrawing and Donating Substituents on the Phenyl Ring
Source: Pharmaceuticals (Basel). 2022 Feb 24;15(3):283. doi: 10.3390/ph15030283 (PMC8949145; doi:10.3390/ph15030283)
Supplement: Supplementary file 1 [file pharmaceuticals-15-00283-s001.zip › pharmaceuticals-1578546-supplementary.pdf]

Supporting information 1

Structure-Activity Relationship of *N*-phenylthieno[2,3-*b*]pyridine-2-carboxamide derivatives designed as Forkhead Box M1 inhibitors: the effect of electron-withdrawing and donating substituents on the phenyl ring

César Sebastian Huerta-García[a], David J. Pérez[b,c], Carlos A. Velázquez-Martínez[b], Seyed Amirhossein Tabatabaei Dakhili[b], Antonio Romo-Mancillas[d], Rafael Castillo[a], Alicia Hernández-Campos[a,*]

[a] Facultad de Química, Departamento de Farmacia, Universidad Nacional Autónoma de México, México, CdMx 04510, Mexico.

[b] Faculty of Pharmacy and Pharmaceutical Sciences, University of Alberta, Edmonton, AB, Canada, T6E 2E1.

[c] Unidad Radiofarmacia-Ciclotrón, División de Investigación, Facultad de Medicina, Universidad Nacional Autónoma de México, 04510, Mexico City, México

[d] Laboratorio de Diseño Asistido por Computadora y Síntesis de Fármacos, Facultad de Química, Universidad Autónoma de Querétaro, Centro Universitario, Querétaro, 76010, Mexico

* Corresponding author

Content table

1. CHEMISTRY 1

1.1. GENERAL INFORMATION 1
1.2. GENERAL METHODS FOR THE SYNTHESIS OF THE 2-CHLORO-*N*-PHENYLACETAMIDES (cF, c1-c18) 2
1.3. PROCEDURE FOR THE SYNTHESIS OF 6-(THIOPHEN-2-YL)-2-THIOXO-4-(TRIFLUOROMETHYL)-1,2-DIHYDROPYRIDINE-3-CARBONITRILE (TPR) 9
1.4. YIELDS FROM THE OBTENTION REACTIONS OF FINAL COMPOUNDS (FDI-6, AND 1–19) 10
1.5. SPECTRA OF COMPOUNDS FDI-6, 1–18 11

2. WESTERN BLOT 47

2.1. BLOT IMAGES 47

3. CELL PROLIFERATION INHIBITION (MTT) ASSAY 53

3.1. CELL VIABILITY CURVES 53
3.2. CELL VIABILITY RESULTS FOR EACH CONCENTRATION 54

4. DOCKING 56

4.1. COMPLETE ENERGY RESULTS 56
4.2. BEST DOCKING POSE OF EACH COMPOUND 59

5. MEP MAPS 61

6. ADDITIONAL REFERENCES 62

1. Chemistry

1.1. General information

Anhydrous dichloromethane, triethylamine, dimethyl sulfoxide (DMSO), acetone, the corresponding anilines, chloroacetyl chloride, 4,4,4-trifluoro-1-(thiophen-2-yl)butane-1,3-dione and 2-cyanothiocetamide were purchased from Sigma-Aldrich and used without further purification. Sodium bicarbonate, anhydrous sodium sulfate, ethyl acetate (EtOAc), methanol, toluene, chloroform, *N*,*N*-dimethylformamide (DMF), sulfuric acid, and potassium carbonate were purchased from J.T. Baker.

Melting points (mp) were determined on a Büchi B-540 apparatus and are uncorrected. Reactions were monitored by TLC on 0.2 pre-coated silica gel 60 F_{254} plates (Merck).

Flash chromatography purifications were carried out in a CombiFlash EZ Prep equipment. Microwave reactions were performed in a Biotage Initiator+ Microwave Synthesizer.

^{1}H NMR and ^{13}C NMR spectra were recorded either on a 9.4 T Varian VNMRS equipped with a Broad Band Switchable probe of two radio frequency channels (^{1}H/^{19}F) (^{31}P/^{15}N) or in a 14.1 T Bruker Avance III equipped with a SmartProbe. The deuterated solvents used were dimethyl sulfoxide (DMSO-d_6) from Cambridge Isotope Laboratories and chloroform ($CDCl_3$) from Sigma-Aldrich. Chemical shifts (δ) are given in ppm relative to tetramethylsilane (Me_4Si, δ= 0) in DMSO-d_6; *J* values are given in Hz. The following abbreviations are used: s, singlet; bs, broad signal; d, doublet; dd, doublet of doublet; t, triplet; m, multiplet.

Mass spectroscopy was recorded either through electronic impact mass spectroscopy (EIMS) using a gas chromatography Perkin Elmer Clarus 680 with a 30 m column and a DB5 stationary phase, coupled to a mass spectrometer Perkin Elmer Clarus SQ 8 C using 200 ºC and 70 eV in the ionization chamber and a spectral window between 33-500 u, or through high-resolution mass spectrometry (HRMS) on a Perkin Elmer AxION 2 TOF coupled to an AxION DSA module using APCI as the ionization technique, with a crown temperature of 280 ºC and 3 µA, using N_2 as drying gas at 4 L/min and a spectral window between 50-3000 u.

IR spectra were determined using an FT-IR Spectrum 400 from Perkin Elmer equipped with the Perkin Elmer ATR universal accessory, with a resolution of 4 cm^{-1}.

1.2. General methods for the synthesis of the 2-chloro-*N*-phenylacetamides (cF, c1-c18)

Method A (cF, c5, c9, c14, c15)

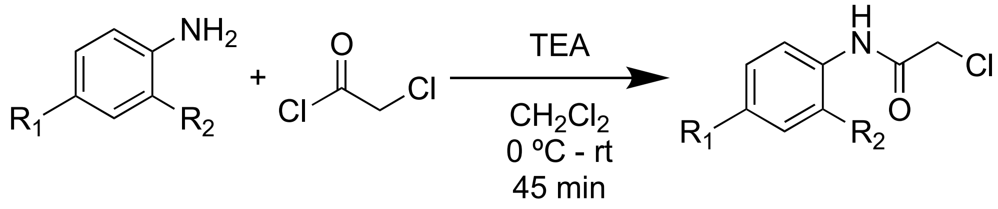

cF: R_1 = -F, R_2 = -H
c5: R_1 = -Cl, R_2 = -H
c9: R_1 = -Br, R_2 = -H
c14: R_1 = -I, R_2 = -H
c15: R_1 = -I, R_2 = $-CH_3$

The appropriate aniline (1 equiv.), triethylamine (1.1 equiv.), and dichloromethane were added to a 50 mL round-bottomed flask equipped with a magnetic stirrer. The flask was placed on an ice mixture and stirred vigorously, then chloroacetyl chloride (1.1 equiv.) was added dropwise to the reaction mixture. The reaction was monitored by TLC, using hexane:ethyl acetate 90:10 with 1% of NH_4OH as an eluent system. Once the starting materials were not seen (as judged by the TLC), the solvent was distilled from the reaction mixture under reduced pressure. The crude product was dissolved in 100 mL EtOAc, and this was transferred to a separation funnel and washed with water (2 x 60 mL). The organic phase was collected, dried with anhydrous Na_2SO_4, the solvent was removed under reduced pressure to afford the corresponding phenylacetamide in a quantitative yield.

2-Chloro-*N*-(4-fluorophenyl)acetamide (cF)

p-Fluoroaniline (0.85 mL, 9.01 mmol), chloroacetyl chloride (0.79 mL, 9.92 mmol), triethylamine (1.38 mL, 9.92 mmol). Silver-colored solid (1. 78 g, 9.00 mmol). **mp:** 132.2–133.0 °C. **^{1}H NMR** (400 MHz, DMSO-d_6, δ in ppm): 10.33 (s, 1H, Int D_2O), 7.60 (dd, *J* = 9.1, 5.0 Hz, 2H), 7.17 (t, *J* = 8.9 Hz, 2H), 4.24 (s, 2H). **IR** (ATR-FTIR, cm^{-1}): 3274 ($\mathbf{v}_{CON-H}$), 3102 ($\mathbf{v}_{Ar-H}$), 2951 ($\mathbf{v}_{C-H}$), 1666 ($\mathbf{v}_{NHC=O}$), 1211 ($\mathbf{v}_{C-F}$), 747 ($\mathbf{v}_{C-Cl}$). **MS** (EI, [M^+], *m/z*): 197 (Calculated: 197 for C_8H_7ClFNO).

2-Chloro-*N*-(4-chlorophenyl)acetamide (c5)

p-Chloroaniline (1.0 g, 7.84 mmol), chloroacetyl chloride (0.69 mL, 8.62 mmol), triethylamine (1.20 mL, 8.62 mmol). Green colored solid (1.58 g, 7.80 mmol). **mp:** 168.6–171.6 ºC. **^{1}H NMR** (400 MHz, DMSO-d_6, δ in ppm): 10.42 (s,1H, Int D_2O), 7.62 (d, *J* = 8.9 Hz, 1H), 7.39 (d, *J* = 8.9 Hz, 1H), 4.25 (s, 2H). **IR** (ATR-FTIR, cm^{-1}): 3263 ($\mathbf{v}_{CON-H}$), 3084 ($\mathbf{v}_{Ar-H}$), 2954–2858 ($\mathbf{v}_{C-H}$), 1667 ($\mathbf{v}_{NHC=O}$), 736 ($\mathbf{v}_{C-Cl}$).

***N*-(4-Bromophenyl)-2-chloroacetamide (c9)**

p-Bromoaniline (1.15 g, 6.69 mmol), chloroacetyl chloride (0.53 mL, 6.69 mmol), triethylamine (0.93 mL, 6.69 mmol). White colored solid (1.65 g, 6.69 mmol). **mp:** 182.8–184.1 ºC. **^{1}H NMR** (400 MHz, DMSO-d_6, δ in ppm): 10.41 (s, 1H, Int D_2O), 7.58–7.50 (m, 4H), 4.25 (s, 2H). **IR** (ATR-FTIR, cm^{-1}): 3263 ($\mathbf{v}_{CON\text{-}H}$), 3079 ($\mathbf{v}_{Ar\text{-}H}$), 2954 ($\mathbf{v}_{C\text{-}H}$), 1667 ($\mathbf{v}_{NHC=O}$), 735 ($\mathbf{v}_{C\text{-}Cl}$). **MS** (EI, [M$^+$], *m/z*): 247 (Calculated: 247 for $C_8H_7BrClNO$).

2-Chloro-*N*-(4-iodophenyl)acetamide (c14)

p-Iodoaniline (1.0 g, 4.59 mmol), chloroacetyl chloride (0.40 mL, 5.05 mmol), triethylamine (0.70 mL, 5.05 mmol). Yellow-colored solid (1.33 g, 4.50 mmol). **mp:** 192.4–194.8 ºC. **^{1}H NMR** (400 MHz, $CDCl_3$, δ in ppm): 8.20 (s, 1H, Int D_2O), 7.66 (d, *J* = 8.8 Hz, 2H), 7.34 (d, *J* = 8.7 Hz, 2H), 4.18 (s, 2H). **IR** (ATR-FTIR, cm^{-1}): 3258 ($\mathbf{v}_{CON\text{-}H}$), 3071 ($\mathbf{v}_{Ar\text{-}H}$), 2946 ($\mathbf{v}_{C\text{-}H}$), 1674 ($\mathbf{v}_{NHC=O}$), 820 ($\mathbf{v}_{C\text{-}Cl}$), 745 y 499 ($\mathbf{v}_{C\text{-}I}$). **MS** (EI, [M$^+$], *m/z*): 295 (Calculated: 295 for C_8H_7IClNO).

2-Chloro-*N*-(4-iodo-2-methylphenyl)acetamide (c15)

4-Iodo-2-methylaniline (300 mg, 1.29 mmol), chloroacetyl chloride (0.11 mL, 1.42 mmol), triethylamine (0.20 mL, 1.42 mmol). Yellow-colored solid (386 mg, 1.25 mmol). **mp:** 158.6–159.8 ºC. **^{1}H NMR** (400 MHz, $CDCl_3$, δ in ppm): 9.55 (s, 1H, Int D_2O), 7.62 (dd, *J* = 2.1, 0.8 Hz, 1H), 7.53 (dd, *J* = 8.5, 2.1 Hz, 1H), 7.24 (d, *J* = 8.4 Hz, 1H), 4.30 (s, 2H), 2.17 (s, 3H). **IR** (ATR-FTIR, cm^{-1}): 3251 ($\mathbf{v}_{CON\text{-}H}$), 3040 ($\mathbf{v}_{Ar\text{-}H}$), 2952–2862 ($\mathbf{v}_{C\text{-}H}$), 1667 ($\mathbf{v}_{NHC=O}$), 813 ($\mathbf{v}_{C\text{-}Cl}$), 686 y 534 ($\mathbf{v}_{C\text{-}I}$). **MS** (EI, [M$^+$], *m/z*): 309 (Calculated: 309 for C_9H_9IClNO).

Method B (c1–c4, c7, c8, c10, c11, c13, c16, c18)

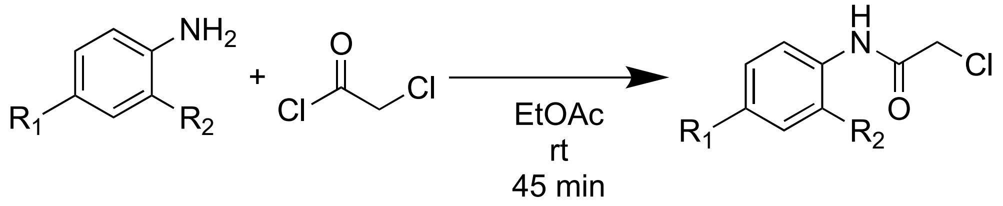

c1: R_1 = -F, R_2 = -CH_3
c2: R_1 = -F, R_2 = -CN
c3: R_1 = -F, R_2 = -NO_2
c4: R_1 = -F, R_2 = -CF_3
c7: R_1 = -Cl, R_2 = -NO_2
c8: R_1 = -Cl, R_2 = -CF_3
c10: R_1 = -Br, R_2 = -CH_3
c11: R_1 = -Br, R_2 = -CN
c13: R_1 = -Br, R_2 = -CF_3
c16: R_1 = -I, R_2 = -CN
c18: R_1 = -I, R_2 = -CF_3

The appropriate aniline (1 equiv.) and EtOAc were added to a 50 mL round-bottomed flask and stirred, followed by the addition of chloroacetyl chloride (1.1 equiv.). The reaction mixture was stirred for 45 minutes and monitored by TLC using Hex/EtOAc 90:10 elution system with 1% of NH_4OH (2x).

Subsequently, the reaction mixture was transferred to a separatory funnel and washed with water (2 x 30 mL). The organic phase was collected, treated with anhydrous Na_2SO_4, and concentrated by distillation under reduced pressure to obtain the product as a solid. The corresponding phenylacetamides were dried under vacuum for a period of 12 hours. All the products were obtained with quantitative yields.

***N*-(Chloromethyl)-4-fluoro-2-methylbenzamide (c1)**

4-Fluoro-2-methylaniline (500 mg, 3.53 mmol), chloroacetyl chloride (0.31 mL, 3.88 mmol). Beige solid (703 mg, 3.50 mmol). **mp:** 121–123 °C. **^{1}H NMR** (600 MHz, $CDCl_3$, δ in ppm): 8.12 (s, 1H, IntD_2O), 7.74 (m, 1H), 6.93 (m, 2H), 4.23 (s, 2H), 2.29 (s, 3H). **^{13}C NMR** (150 MHz, $CDCl_3$, δ in ppm): 164.1, 160.4 (d, *J* = 245.3 Hz), 132.5 (d, *J* = 8.13 Hz), 130.6 (d, *J* = 2.98 Hz), 124.9 (d, *J* = 8.63 Hz), 117.3 (d, *J* = 22.52 Hz), 113.6 (d, *J* = 22.40 Hz), 43.2, 17.8.

***N*-(Chloromethyl)-2-cyano-4-fluorobenzamide (c2)**

2-Amino-5-fluorobenzonitrile (300 mg, 2.20 mmol), chloroacetyl chloride (0.26 mL, 2.42 mmol). White solid (466 mg, 2.20 mmol). **mp:** 103–105 °C. **^{1}H NMR** (600 MHz, $CDCl_3$, δ in ppm): 8.80 (s, 1H, IntD_2O), 8.35 (dd, *J* = 9.1, 4.8 Hz, 1H), 7.36 (dt, *J* = 8.3, 1.1, 0.3 Hz, 1H), 7.34 (tdd, *J* = 7.5, 7.4, 3.0, 0.6 Hz, 1H),

4.25 (s, 2H). **^{13}C NMR** (150 MHz, $CDCl_3$, δ in ppm): 164.5, 158.7 (d, *J* = 248.8 Hz), 123.6 (d, *J* = 8.1 Hz), 121.9 (d, *J* = 22.3 Hz), 119.0 (d, *J* = 25.6 Hz), 114.8 (d, *J* = 2.8 Hz), 104.5 (d, *J* = 9.1 Hz).

***N*-(Chloromethyl)-4-fluoro-2-nitrobenzamide (c3)**

4-Fluoro-2-nitroaniline (1.0 g, 6.41 mmol), chloroacetyl chloride (0.61 mL, 7.05 mmol). Orange solid (1.48 g, 6.40 mmol). **mp:** 94.2–95.2 ºC. **^{1}H NMR** (400 MHz, DMSO-d_6, δ in ppm): 10.63 (s, 1H, Int D_2O), 7.96 (dd, *J* = 8.5, 2.9 Hz, 1H), 7.76 (dd, *J* = 9.1, 5.2 Hz, 1H), 7.67 (ddd, *J* = 9.0, 7.8, 3.0 Hz, 1H), 4.35 (s, 2H). **IR** (ATR-FTIR, cm^{-1}): 3323 ($\mathbf{v}_{CON\text{-}H}$), 3092 ($\mathbf{v}_{Ar\text{-}H}$), 2965 ($\mathbf{v}_{C\text{-}H}$), 1684 ($\mathbf{v}_{NHC=O}$), 1508 ($\mathbf{v}_{Ar\text{-}NO2}$)1248 ($\mathbf{v}_{C\text{-}F}$), 676 ($\mathbf{v}_{C\text{-}Cl}$). **MS** (EI, [$M^+$], *m/z*): 232 (Calculated: 232 for $C_8H_6ClFN_2O_3$).

***N*-(Chloromethyl)-4-fluoro-2-(trifluoromethyl)benzamide (c4)**

4-Fluoro-2-(trifluoromethyl)aniline (500 mg, 2.79 mmol), chloroacetyl chloride (0.27 mL, 3.07 mmol). Beige solid (701 mg, 2.75 mmol). **mp:** 89–92 ºC. **^{1}H NMR** (600 MHz, $CDCl_3$, δ in ppm): 8.64 (s, 1H, IntD_2O), 8.16 (dd, *J* = 9.1, 4.9 Hz, 1H), 7.36 (dd, *J* = 8.4, 3.0 Hz, 1H), 7.30 (m, 1H), 4.23 (s, 2H). **^{13}C NMR** (150 MHz, $CDCl_3$, δ in ppm): 164.5, 159.4 (d, *J* = 247.83 Hz), 126.8 (d, *J* = 7.8), 123.0 (dd, *J* = 273.3, 2.4 Hz), 123.0 (dd, *J* = 31.1, 7.5 Hz), 120.0 (d, *J* = 22.0 Hz), 113.9 (dq, *J* = 26.3, 5.4, 5.4, 5.4 Hz), 42.9.

4-Chloro-*N*-(chloromethyl)-2-cyanobenzamide (c6)

2-Amino-5-chlorobenzonitrile (500 mg, 3.27 mmol), chloroacetyl chloride (0.39 mL, 3.60 mmol). White solid (741 mg, 3.25 mmol). **mp:** 122.9–124.7 ºC. **^{1}H NMR** (600 MHz, $CDCl_3$, δ en ppm): 10.56 (s, 1H, Int D_2O), 8.04 (d, *J* = 2.5 Hz, 1H), 7.79 (dd, *J* = 8.8, 2.5 Hz, 1H), 7.66 (dd, *J* = 8.8 Hz, 1H), 4.38 (s, 2H). **IR** (ATR-FTIR, cm^{-1}): 3336 ($\mathbf{v}_{CON\text{-}H}$), 3066 ($\mathbf{v}_{Ar\text{-}H}$), 2961 ($\mathbf{v}_{C\text{-}H}$), 2229 ($\mathbf{v}_{C\equiv N}$), 1699 ($\mathbf{v}_{NHC=O}$), 834 ($\mathbf{v}_{C\text{-}Cl}$). **MS** (EI, [$M^+$], *m/z*): 228 (Calculated: 228 for $C_9H_6Cl_2N_2O$).

4-Chloro-*N*-(chloromethyl)-2-(trifluoromethyl)benzamide (c8)

4-Chloro-2-(trifluoromethyl)aniline (0.36 mL, 2.56 mmol), chloroacetyl chloride (0.22 mL, 2.81 mmol). White solid (691 mg, 2.55 mmol). **mp:** 80–81 °C. **^{1}H NMR** (600 MHz, $CDCl_3$, δ in ppm): 8.73 (s, 1H, IntD_2O), 8.22 (d, *J* = 8.9 Hz, 1H), 7.63 (d, *J* = 2.5 Hz, 1H), 7.55 (dd, *J* = 8.8, 2.5 Hz, 1H), 4.23 (s, 2H). **^{13}C NMR** (150 MHz, $CDCl_3$, δ in ppm): 164.4, 133.1, 133.0, 130.9, 126.6 (q, *J* = 5.5 Hz), 125.3, 123.2 (d, *J* = 273.7 Hz), 122.0 (d, *J* = 30.9 Hz), 43.0.

4-Bromo-*N*-(chloromethyl)-2-methylbenzamide (c10)

4-Bromo-2-methylaniline (500 mg, 2.69 mmol), chloroacetyl chloride (0.23 mL, 2.96 mmol). White solid (692 mg, 2.65 mmol). **mp:** 130–132 °C. **^{1}H NMR** (600 MHz, $CDCl_3$, δ in ppm): 8.19 (s, 1H, IntD_2O), 7.82 (d, *J* = 9.3 Hz, 1H), 7.36 (m, 2H), 4.23 (s, 2H), 2.28 (s, 3H). **^{13}C NMR** (150 MHz, $CDCl_3$, δ in ppm): 163.9, 134.0, 133.4, 131.0, 130.1, 123.8, 118.7, 43.3, 17.5.

4-Bromo-*N*-(chloromethyl)-2-cyanobenzamide (c11)

2-Amino-5-bromobenzonitrile (558 mg, 2.83 mmol), chloroacetyl chloride (0.34 mL, 3.12 mmol). White solid (762 mg, 2.80 mmol). **mp:** 114.0–116.2 ºC. **^{1}H NMR** (400 MHz, DSMO-d_6, δ in ppm): 10.55 (s, 1H, Int D_2O), 8.15 (d, *J* = 2.3 Hz, 1H), 7.91 (dd, *J* = 8.8, 2.4 Hz, 1H), 7.59 (d, *J* = 8.8 Hz, 1H), 4.37 (s, 2H). **IR** (ATR-FTIR, cm^{-1}): 3332 ($\mathbf{v}_{CON-H}$), 3064 ($\mathbf{v}_{Ar-H}$), 2959 ($\mathbf{v}_{C-H}$), 2228 ($\mathbf{v}_{C≡N}$), 1695 ($\mathbf{v}_{NHC=O}$), 822 ($\mathbf{v}_{C-Cl}$). **MS** (EI, [M^+], *m/z*): 272 (Calculated: 272 for $C_9H_6BrClN_2O$).

4-Bromo-*N*-(chloromethyl)-2-(trifluoromethyl)benzamide (c13)

4-Bromo-2-(trifluoromethyl)aniline (1.49 g, 6.21 mmol), chloroacetyl chloride (0.54 mL, 6.83 mmol). White solid (1.95 g, 6.20 mmol). **mp:** 92.3–94.0 ºC. **^{1}H NMR** (400 MHz, DMSO-d_6, δ in ppm): 9.97 (s, 1H, Int D_2O), 7.94 (q, J = 2.1 Hz, 1H), 7.91 (dd, J = 8.2, 2.3 Hz, 1H), 7.50 (dd, J = 8.3, 0.7 Hz, 1H), 4.32 (s, 2H). **IR** (ATR-FTIR, cm^{-1}): 3260 ($\mathbf{v}_{CON-H}$), 3036 ($\mathbf{v}_{Ar-H}$), 2960 ($\mathbf{v}_{C-H}$), 1678 ($\mathbf{v}_{NHC=O}$), 1308 ($\mathbf{v}_{C-F}$), 660 ($\mathbf{v}_{C-Cl}$). **MS** (EI, [M^+], *m/z*): 315 (Calculated: 315 for $C_9H_6BrClF_3NO$).

***N*-(Chloromethyl)-2-cyano-4-iodobenzamide (c16)**

2-Amino-5-iodobenzonitrile (1.32 g, 5.41 mmol), chloroacetyl chloride (0.47 mL, 5.95 mmol). Gray solid (1.73 g, 5.40 mmol). **^{1}H NMR** (600 MHz, $CDCl_3$, δ in ppm): 10.51 (s, 1H, Int D_2O), 8.22 (d, *J* = 2.0 Hz, 1H), 8.04 (dd, *J* = 8.6, 2.1 Hz, 1H), 7.44 (dd, *J* = 8.7 Hz, 1H), 4.37 (s, 2H). **IR** (ATR-FTIR, cm^{-1}): 3321 ($\mathbf{v}_{CON-H}$), 3057 ($\mathbf{v}_{Ar-H}$), 2955 ($\mathbf{v}_{C-H}$), 2225 ($\mathbf{v}_{C≡N}$), 1692 ($\mathbf{v}_{NHC=O}$), 841 ($\mathbf{v}_{C-Cl}$), 490 ($\mathbf{v}_{C-I}$).

***N*-(Chloromethyl)-4-iodo-2-(trifluoromethyl)benzamide (c18)**

4-Iodo-2-(trifluoromethyl)aniline (1.78 g, 6.20 mmol), chloroacetyl chloride (0.54 mL, 6.82 mmol). Beige solid (2.25 g, 6.20 mmol). **mp:** 128.5–129.2 ºC. **^{1}H NMR** (600 MHz, $CDCl_3$, δ en ppm): 9.93 (s, 1H, Int D_2O), 8.07 – 8.04 (m, 2H), 7.34 (d, *J* = 8.3 Hz, 1H), 4.32 (s, 2H). **IR** (ATR-FTIR, cm^{-1}): 3364 ($\mathbf{v}_{CON-H}$), 3031 ($\mathbf{v}_{Ar-H}$), 2957 ($\mathbf{v}_{C-H}$), 1677 ($\mathbf{v}_{NHC=O}$), 1306 ($\mathbf{v}_{C-F}$), 655 ($\mathbf{v}_{C-Cl}$), 521 y 469 ($\mathbf{v}_{C-I}$). **MS** (EI, [M^+], *m/z*): 363 (Calculated: 363 for $C_9H_6ClF_3INO$).

Method C (c7, c12, c17)

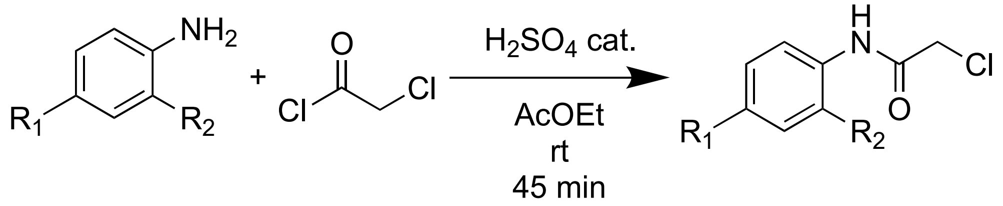

c7: R_1 = -Cl, R_2 = $-NO_2$
c12: R_1 = -Br, R_2 = $-NO_2$
c17: R_1 = -I, R_2 = $-NO_2$

In a 50 mL round-bottomed flask with a magnetic stirrer, the appropriate aniline (1 equiv.) and EtOAc were added and stirred vigorously. Afterward, chloroacetyl chloride (1.1 equiv.) and H_2SO_4 (0.1 equiv.) were added; the reaction was monitored by TLC using Hex/EtOAc 90:10 with 1% of NH_4OH (2x) as the elution system. After 45 min, according to TLC, the starting materials were consumed, and the reaction was stopped.

Subsequently, the reaction mixture was transferred to a separatory funnel with EtOAc (20 mL), and extraction was made using water (2 x 30 mL). The organic phase was collected, treated with anhydrous Na_2SO_4, and concentrated by distillation under reduced pressure to obtain the product as a solid. The products were dried under vacuum for a period of 12 hours. All the products were obtained with quantitative yields.

4-Chloro-*N*-(chloromethyl)-2-nitrobenzamide (c7)

4-Chloro-2-nitroaniline (1.0 g, 5.79 mmol), chloroacetyl chloride (0.50 mL, 6.37 mmol), H_2SO_4 (0.03 mL, 0.58 mmol). Orange solid (1.43 g, 5.75 mmol). **mp:** 140.6–141.5 ºC. **^{1}H NMR** (400 MHz, DMSO-d_6, δ in ppm): 10.71 (s, 1H, Int D_2O), 8.12 (dd, *J* = 2.3, 0.5 Hz, 1H), 7.83 (dd, *J* = 8.8, 2.3 Hz, 1H), 7.80 (dd, *J* = 8.8, 0.6 Hz, 1H), 4.37 (s, 2H). **IR** (ATR-FTIR, cm^{-1}): 3325 ($\mathbf{v}_{CON\text{-}H}$), 3093 ($\mathbf{v}_{Ar\text{-}H}$), 2966 ($\mathbf{v}_{C\text{-}H}$), 1686 ($\mathbf{v}_{NHC=O}$), 1493 y 1341 ($\mathbf{v}_{C\text{-}NO2}$), 751 ($\mathbf{v}_{C\text{-}Cl}$). **MS** (EI, [$M^+$], *m/z*): 248 (Calculated: 248 for $C_8H_6Cl_2N_2O_3$).

4-Bromo-*N*-(chloromethyl)-2-nitrobenzamide (c12)

4-Bromo-2-nitroaniline (752 mg, 3.47 mmol), chloroacetyl chloride (0.30 mL, 3.82 mmol), H_2SO_4 (0.02 mL, 0.35 mmol). Orange solid (1.01 g, 3.45 mmol). **mp:** 132.2–134.4 ºC. **^{1}H NMR** (400 MHz, DMSO-d_6, δ in ppm): 10.70 (s, 1H, Int D_2O), 8.21 (d, *J* = 2.3 Hz, 1H), 7.95 (dd, *J* = 8.7, 2.3 Hz, 1H), 7.74 (d, *J* = 8.8 Hz, 1H), 4.37 (s, 2H). **IR** (ATR-FTIR, cm^{-1}): 3330 ($\mathbf{v}_{CON\text{-}H}$), 3089 ($\mathbf{v}_{Ar\text{-}H}$), 2965 ($\mathbf{v}_{C\text{-}H}$), 1684 ($\mathbf{v}_{NHC=O}$), 1488 y 1336 ($\mathbf{v}_{Ar\text{-}NO2}$), 663 ($\mathbf{v}_{C\text{-}Cl}$). **MS** (EI, [$M^+$], *m/z*): 292 (Calculated: 292 for $C_8H_6BrClN_2O_3$).

***N*-(Chloromethyl)-4-iodo-2-nitrobenzamide (c17)**

4-Iodo-2-nitroaniline (1.0 g, 3.79 mmol), chloroacetyl chloride (0.33 mL, 4.17 mmol), H_2SO_4 (0.02 mL, 0.38 mmol). Orange solid (1.29 g, 3.78 mmol). **mp:** 124.1–124.9 ºC. **^{1}H NMR** (400 MHz, $CDCl_3$, δ in ppm): 11.29 (s, 1H, Int D_2O), 8.55 (m, 2H), 7.95 (dd, *J* = 8.9, 2.0 Hz, 1H), 4.23 (s, 2H). **IR** (ATR-FTIR, cm^{-1}): 3288 ($\mathbf{v}_{CON\text{-}H}$), 3105 ($\mathbf{v}_{Ar\text{-}H}$), 2950 ($\mathbf{v}_{C\text{-}H}$), 1682 ($\mathbf{v}_{NHC=O}$), 1491 ($\mathbf{v}_{C\text{-}NO2}$), 717 ($\mathbf{v}_{C\text{-}Cl}$). **MS** (EI, [$M^+$], *m/z*): 340 (Calculated: 340 for $C_8H_6ClN_2O_3$).

1.3. Procedure for the synthesis of 6-(Thiophen-2-yl)-2-thioxo-4-(trifluoromethyl)-1,2-dihydropyridine-3-carbonitrile (TPR)

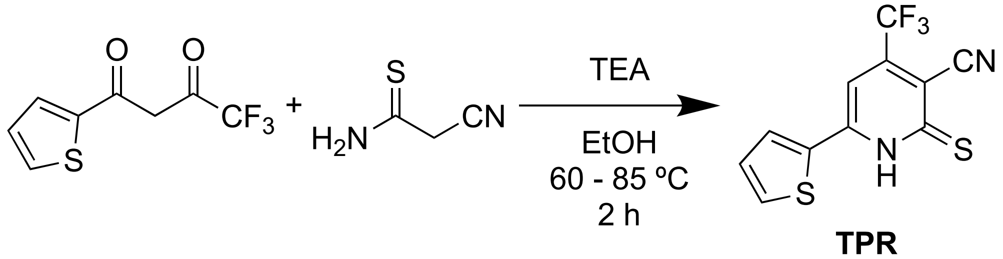

TPR

This methodology was adapted from previous reports [1,2]. In a 50 mL round-bottomed flask with a magnetic stirrer, 4,4,4-trifluoro-1-(thiophen-2-yl)butane-1,3-dione (2.0 g, 9.0 mmol, 1 equiv.) and triethylamine (2.0 mL, 14.31 mmol, 1.59 equiv.) were dissolved in EtOH (10 mL). The flask was immersed in an oil bath at 60 °C and stirring started. After 5 minutes, 2-cyanothiocetamide (901 mg, 9.0 mmol, 1 equiv.) was added to the reaction mixture and heated to 85 °C. After two hours, the reaction was monitored by TLC in a $CHCl_3$/MeOH 80:20 elution system comparing the reaction mixture with both starting materials. Once the starting materials were no longer present in the reaction mixture the heating stopped, whereby the formation of a yellow crystalline solid was observed.

The precipitated solid was filtered off under vacuum and washed using cold water, then the product was allowed to dry under vacuum for 12 hours. A dark yellow crystalline solid was obtained in 84% yield. In the yield calculation, triethylamine is considered in a 1: 1 ratio with TPR. **mp:** 162.8–164.2 ºC. **^{1}H NMR** (400 MHz, DMSO-d_6, δ in ppm): 8.97 (s, 1H), 7.90 (dd, *J* = 3.8, 1.2 Hz, 1H), 7.70 (dd, *J* = 5.1, 1.1 Hz, 1H), 7.29 (s, 1H), 7.16 (dd, *J* = 5.1, 3.7 Hz, 1H), 3.10 (q, 6H), 1.17 (t, 9H). **^{13}C NMR** (100 MHz, DMSO-d_6, δ in ppm) δ 153, 144, 139 (q, *J* = 31.5 Hz), 131, 129, 122 (q, *J* = 274.8 Hz), 118, 117, 104, 103, 46, 9.

1.4. Yields from the obtention reactions of final compounds (FDI-6, and 1–19)

Table S1. Comparison of the yields from the obtention reactions of final compounds (FDI-6, and 1–19) between microwave heating and conventional heating

Conventional heating		Microwave heating	
**Compound**	**Yield (%)**	**Compound**	**Yield (%)**
FDI-6	90	1	91
3	92	2	92
5	95	4	93
6	88	8	90
7	85	10	94
9	94		
11	90		
12	88		
13	92		
14	95		
15	92		
16	91		
17	86		
18	92		

1.5. Spectra of compounds FDI-6, 1–18

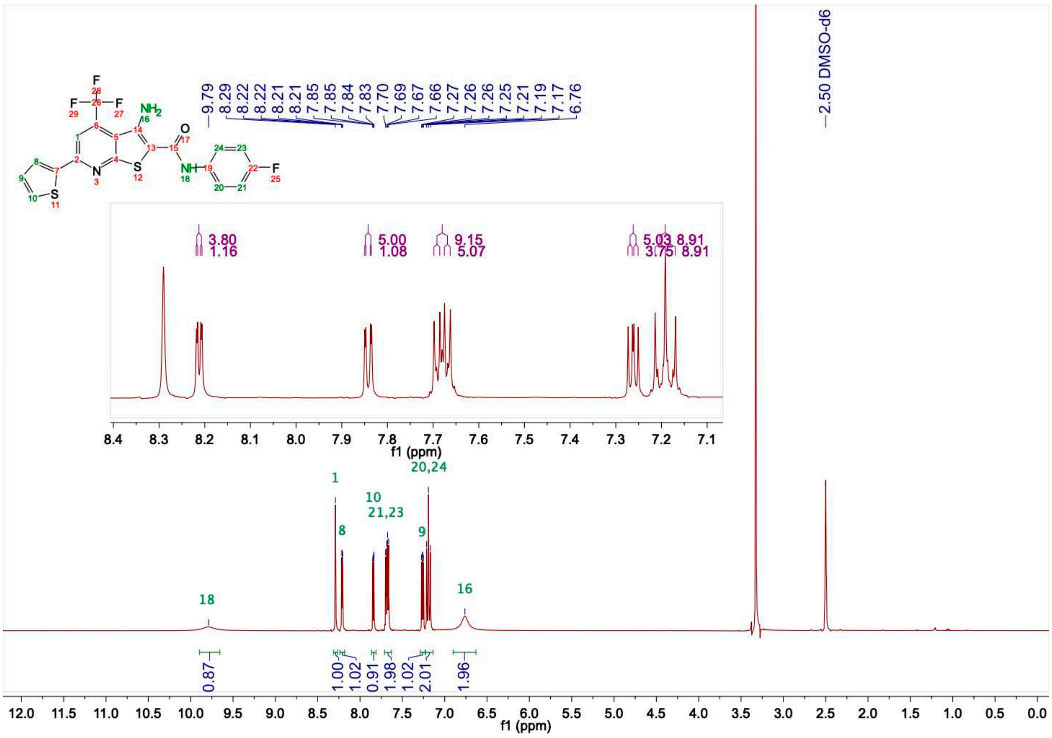

Figure S1. ^{1}H NMR (400 MHz, DMSO-d_6, δ in ppm) 3-Amino-*N*-(4-fluorophenyl)-6-(thiophen-2-yl)-4-(trifluoromethyl)thieno[2,3-*b*]pyridine-2-carboxamide (FDI-6)

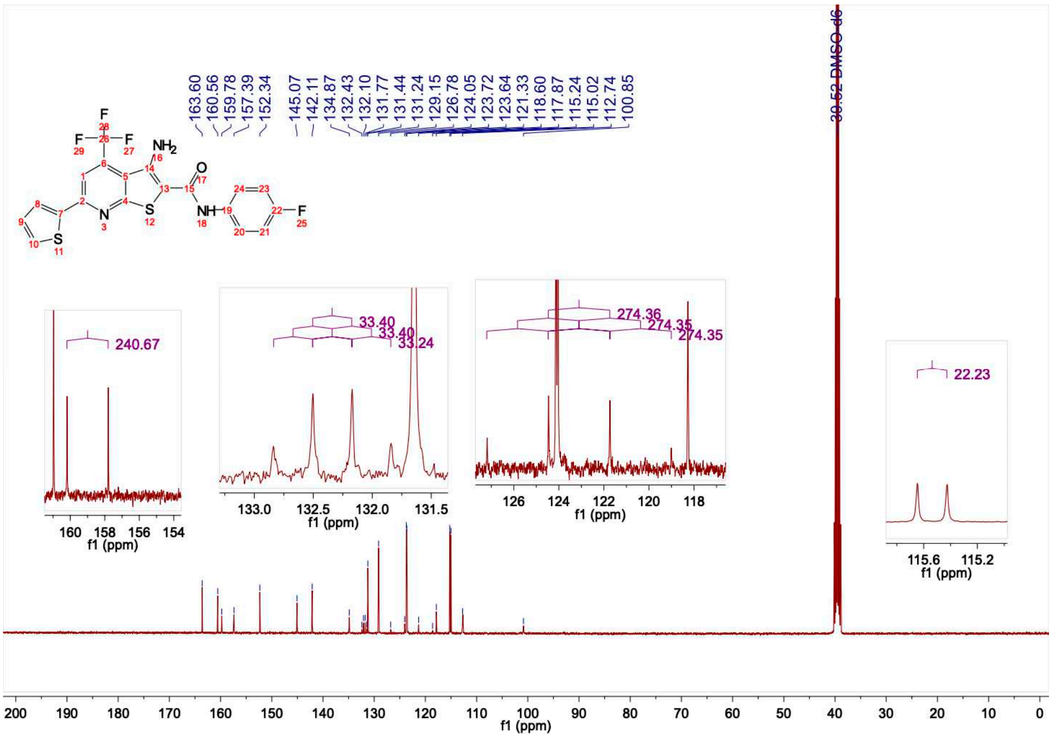

Figure S2. ^{1}H NMR (100 MHz, DMSO-d_6, δ in ppm) 3-Amino-*N*-(4-fluorophenyl)-6-(thiophen-2-yl)-4-(trifluoromethyl)thieno[2,3-*b*]pyridine-2-carboxamide (FDI-6)

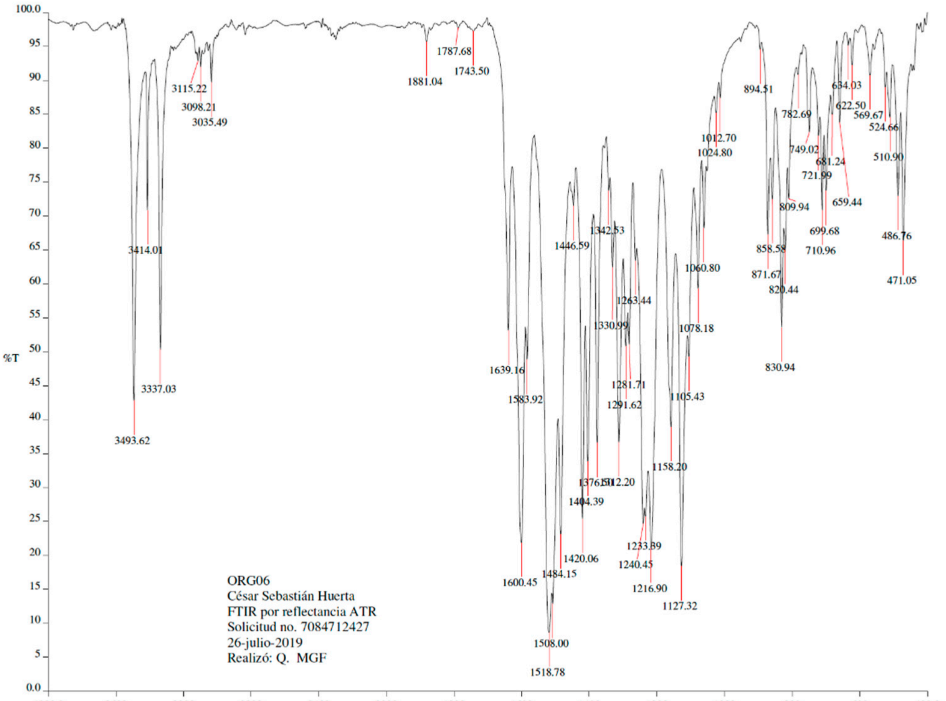

Figure S3. IR (ATR-FTIR, cm^{-1}) 3-Amino-*N*-(4-fluorophenyl)-6-(thiophen-2-yl)-4-(trifluoromethyl)thieno[2,3-*b*]pyridine-2-carboxamide (FDI-6)

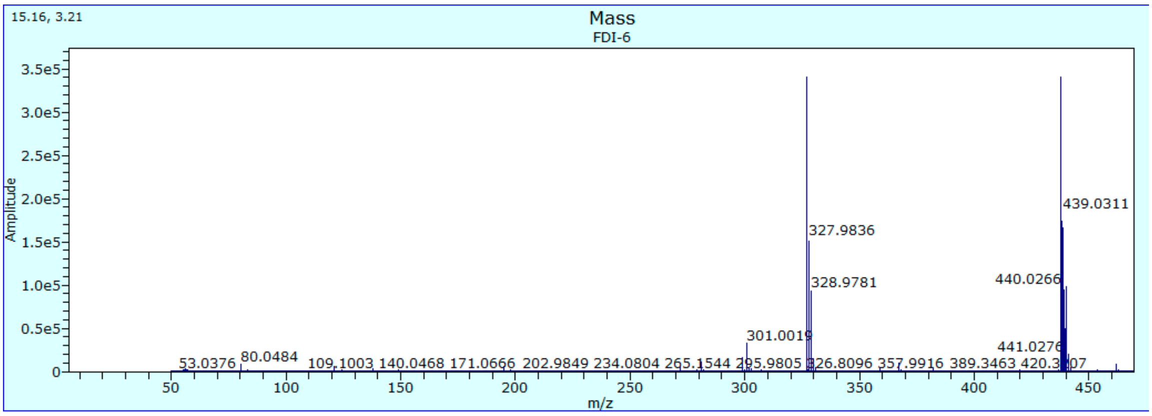

Figure S4. HRMS (APCI, $[M+H]^+$, *m/z*) 3-Amino-*N*-(4-fluorophenyl)-6-(thiophen-2-yl)-4-(trifluoromethyl)thieno[2,3-*b*]pyridine-2-carboxamide (FDI-6)

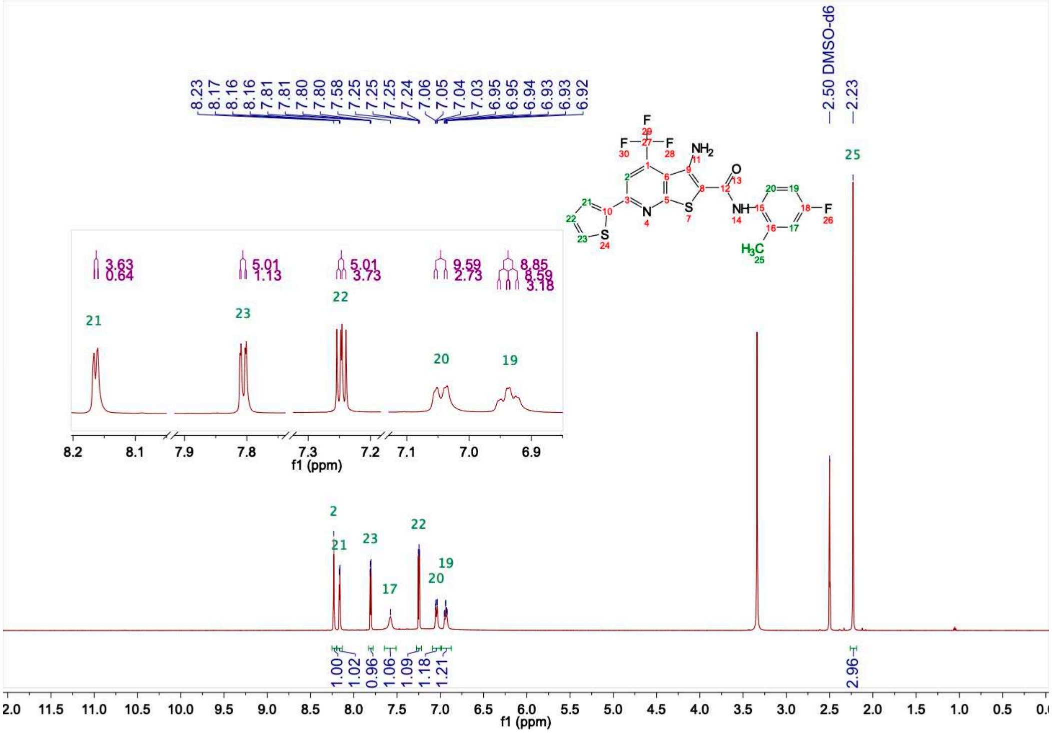

Figure S5. ^{1}H NMR (600 MHz, DMSO-d_6, δ in ppm) 3-Amino-*N*-(4-fluoro-2-methylphenyl)-6-(thiophen-2-yl)-4-(trifluoromethyl)thieno[2,3-*b*]pyridine-2-carboxamide (1)

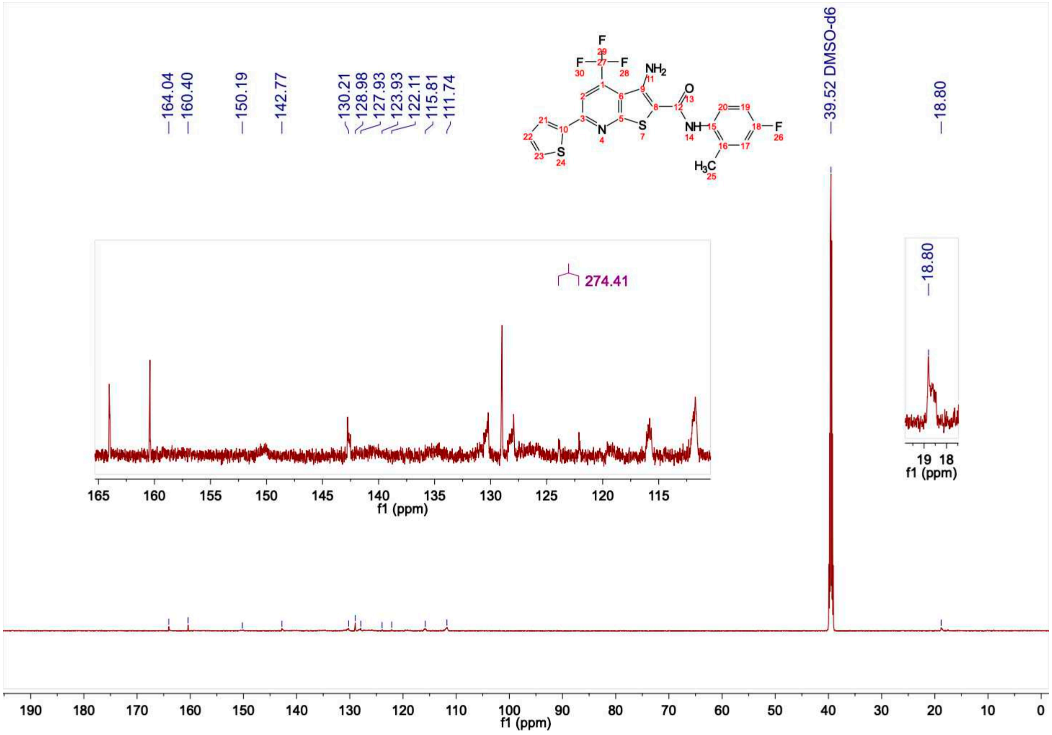

Figure S6. ^{13}C NMR (150 MHz, DMSO-d_6, δ in ppm) 3-Amino-*N*-(4-fluoro-2-methylphenyl)-6-(thiophen-2-yl)-4-(trifluoromethyl)thieno[2,3-*b*]pyridine-2-carboxamide (1)

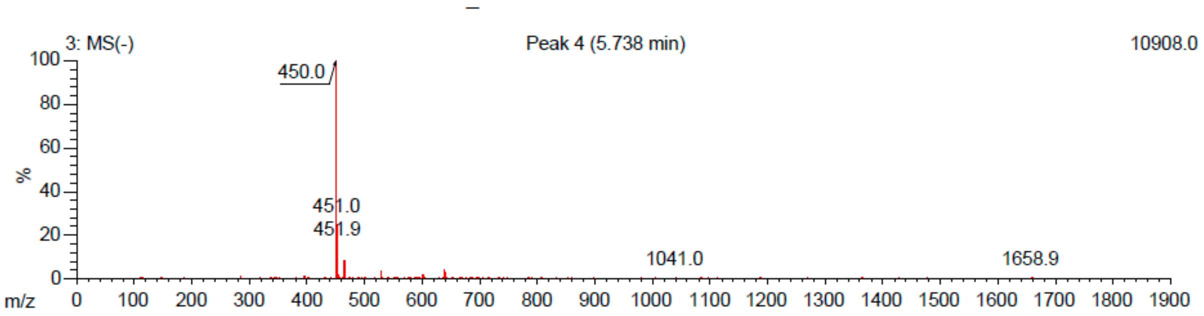

Figure S7. MS (LCMS-DAD/ESI, M$^-$, *m*/*z*) 3-Amino-*N*-(4-fluoro-2-methylphenyl)-6-(thiophen-2-yl)-4-(trifluoromethyl)thieno[2,3-*b*]pyridine-2-carboxamide (1)

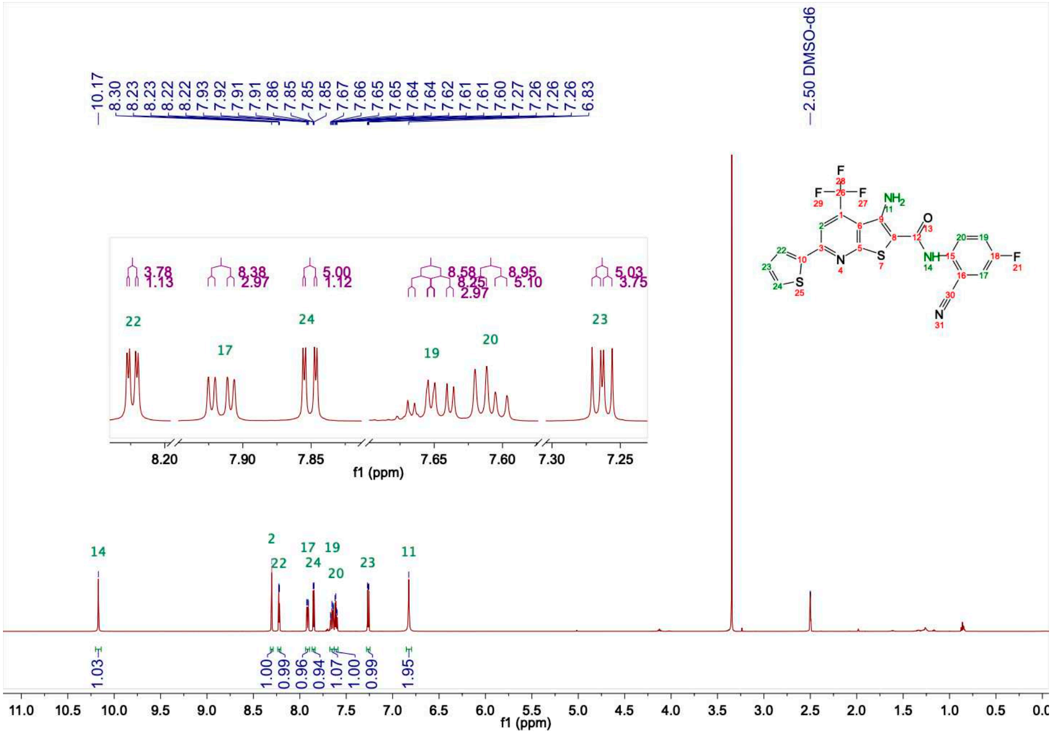

Figure S8. ^{1}H NMR (600 MHz, DMSO-d_6, δ in ppm) 3-Amino-*N*-(2-cyano-4-fluorophenyl)-6-(thiophen-2-yl)-4-(trifluoromethyl)thieno[2,3-*b*]pyridine-2-carboxamide (2)

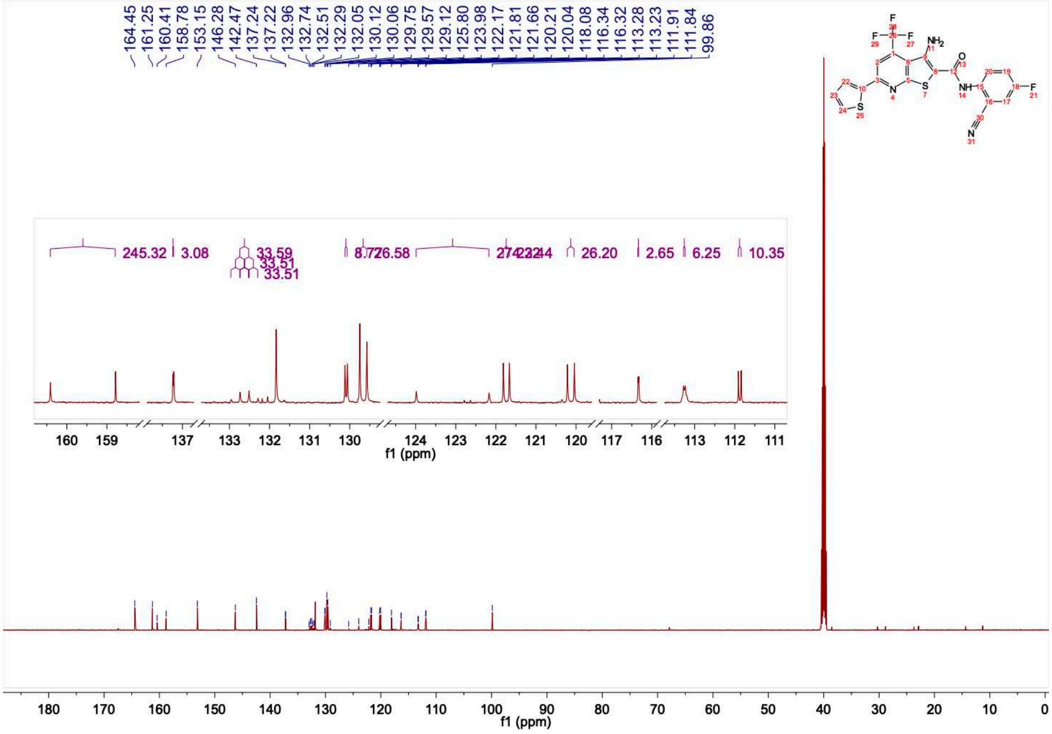

Figure S9. ^{13}C NMR (150 MHz, DMSO-d_6, δ in ppm) 3-Amino-*N*-(2-cyano-4-fluorophenyl)-6-(thiophen-2-yl)-4-(trifluoromethyl)thieno[2,3-*b*]pyridine-2-carboxamide (2)

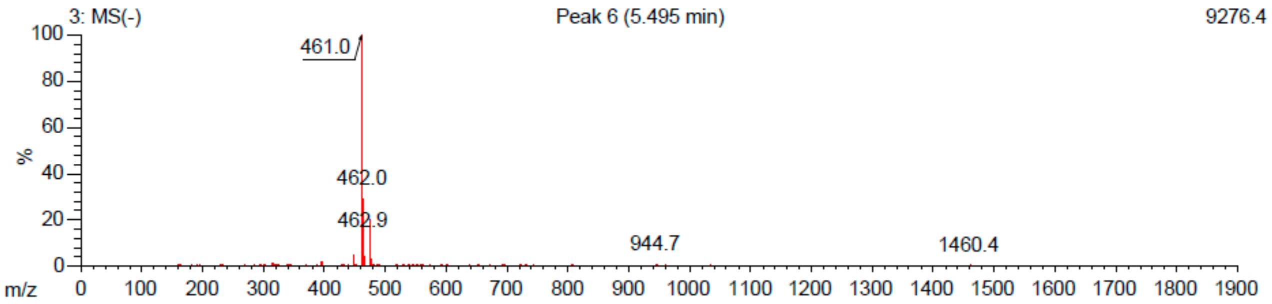

Figure S10. MS (LCMS-DAD/ESI, [M$^-$], *m/z*) 3-Amino-*N*-(2-cyano-4-fluorophenyl)-6-(thiophen-2-yl)-4-(trifluoromethyl)thieno[2,3-*b*]pyridine-2-carboxamide (2)

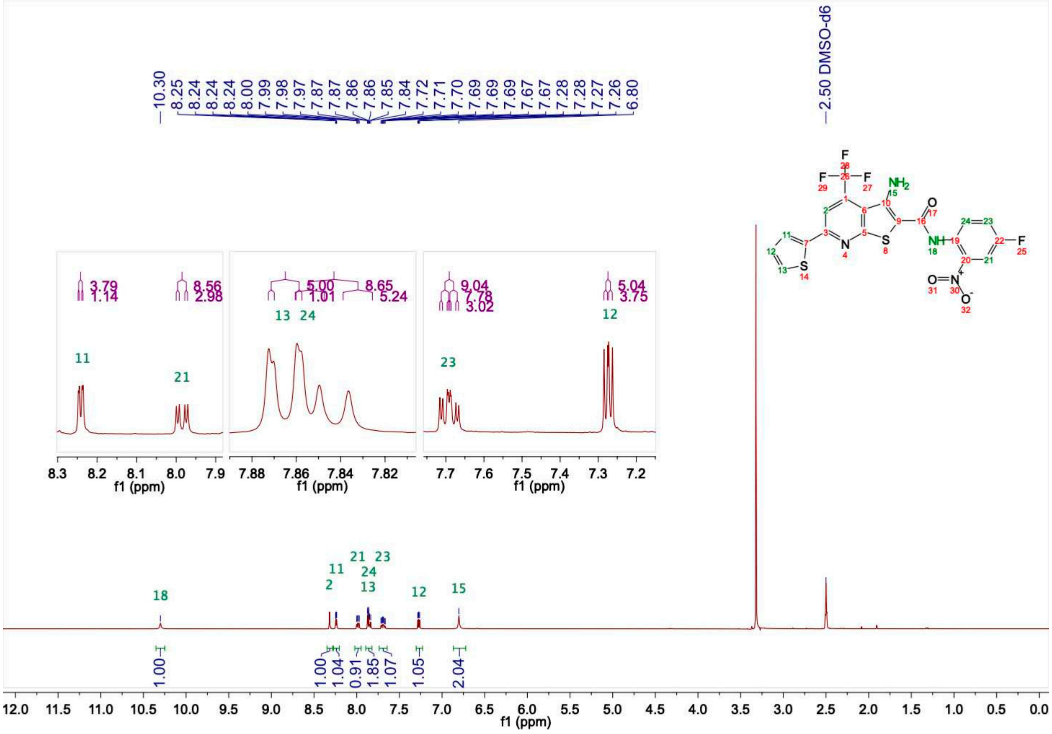

Figure S11. ¹H NMR (400 MHz, DMSO-d_6, δ en ppm) 3-Amino-*N*-(4-fluoro-2-nitrophenyl)-6-(thiophen-2-yl)-4-(trifluoromethyl)thieno[2,3-*b*]pyridine-2-carboxamide (3)

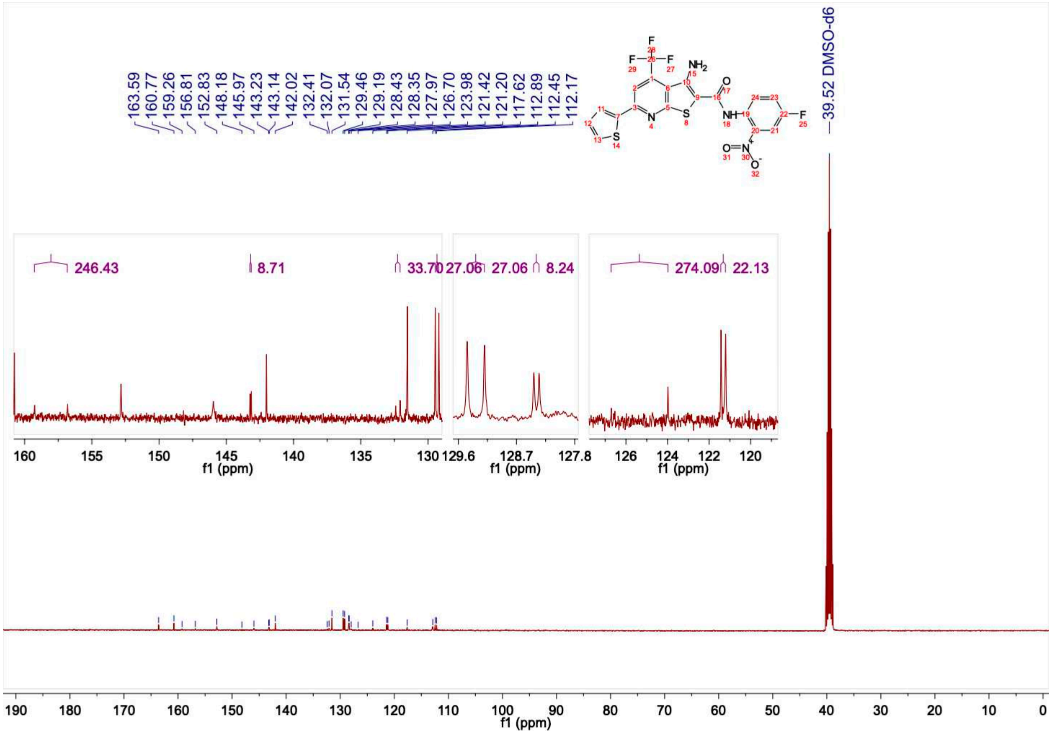

Figure S12. ¹³C NMR (100 MHz, DMSO-d_6, δ en ppm) 3-Amino-*N*-(4-fluoro-2-nitrophenyl)-6-(thiophen-2-yl)-4-(trifluoromethyl)thieno[2,3-*b*]pyridine-2-carboxamide (3)

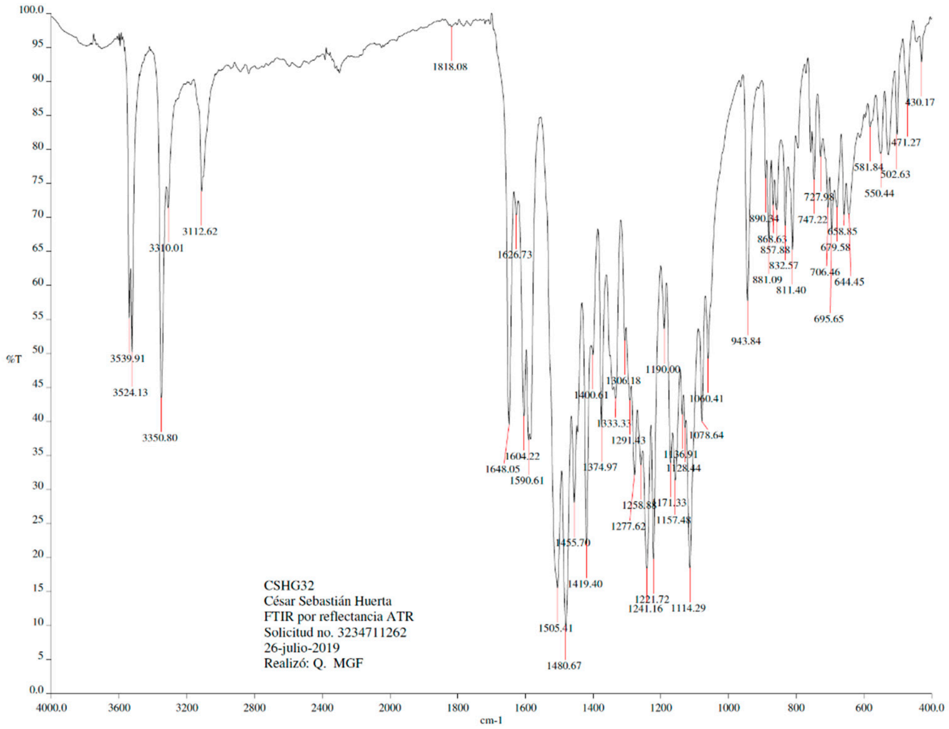

Figure S13. IR (ATR-FTIR, cm^{-1}) 3-Amino-*N*-(4-fluoro-2-nitrophenyl)-6-(thiophen-2-yl)-4-(trifluoromethyl)thieno[2,3-*b*]pyridine-2-carboxamide (3)

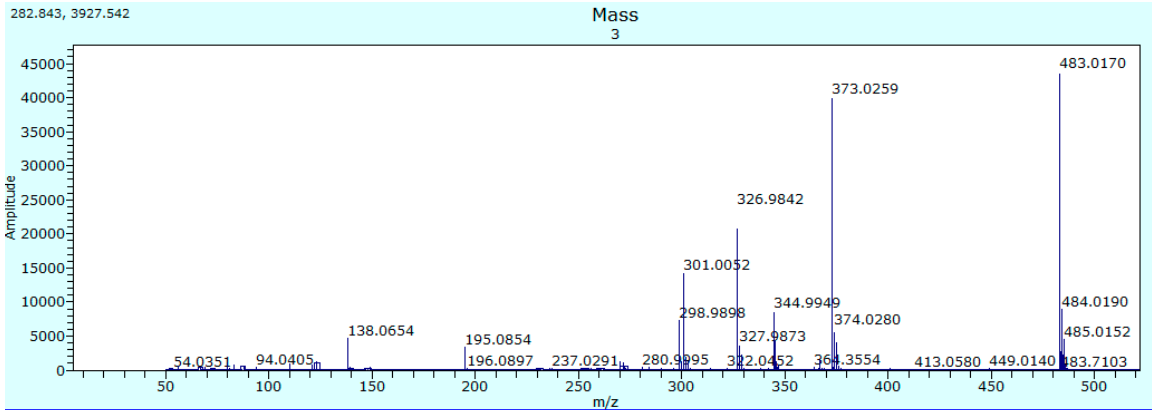

Figure S14. HRMS (APCI, $[M+H]^+$, *m/z*) 3-Amino-*N*-(4-fluoro-2-nitrophenyl)-6-(thiophen-2-yl)-4-(trifluoromethyl)thieno[2,3-*b*]pyridine-2-carboxamide (3)

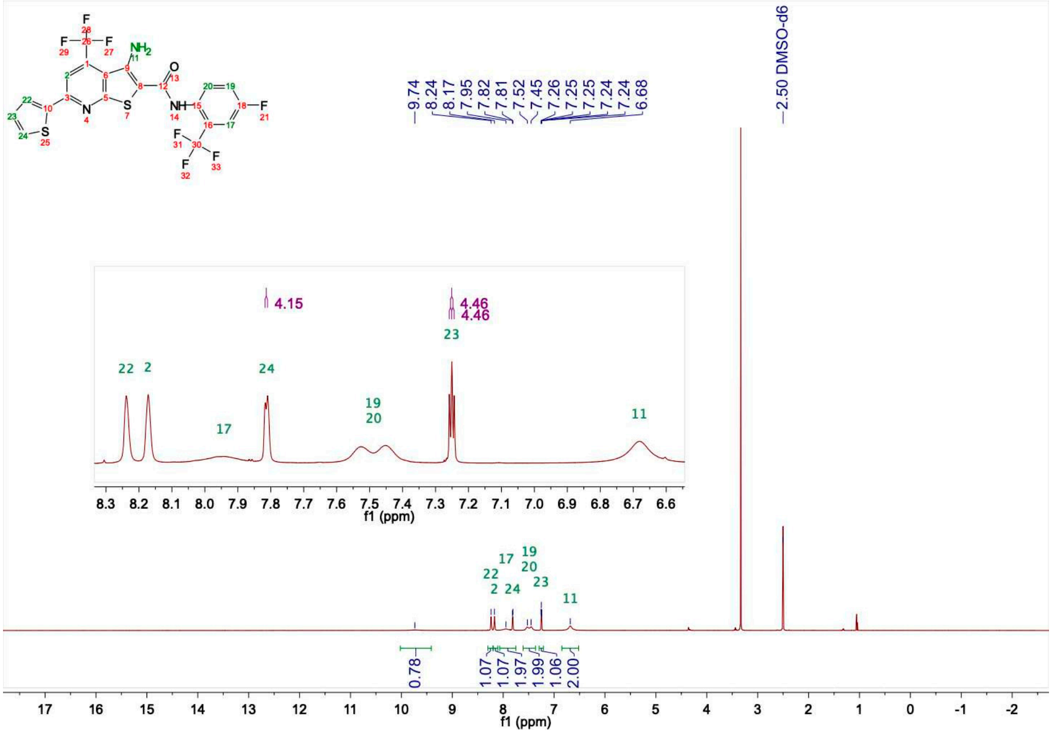

Figure S15. ^{1}H NMR (600 MHz, DMSO-d_6, δ in ppm) 3-Amino-*N*-(4-fluor-2-(trifluoromethyl)phenyl)-6-(thiophen-2-yl)-4-(trifluoromethyl)thieno[2,3-*b*]pyridine-2-carboxamide (4)

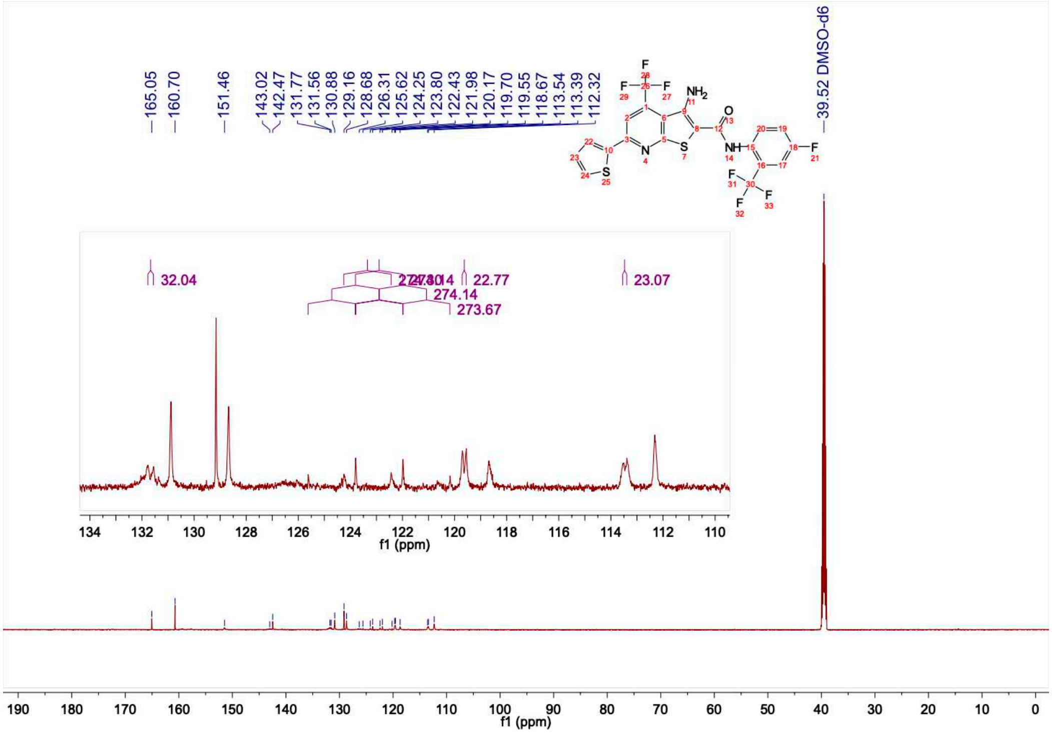

Figure S16. ^{1}H NMR (150 MHz, DMSO-d_6, δ in ppm) 3-Amino-*N*-(4-fluor-2-(trifluoromethyl)phenyl)-6-(thiophen-2-yl)-4-(trifluoromethyl)thieno[2,3-*b*]pyridine-2-carboxamide (4)

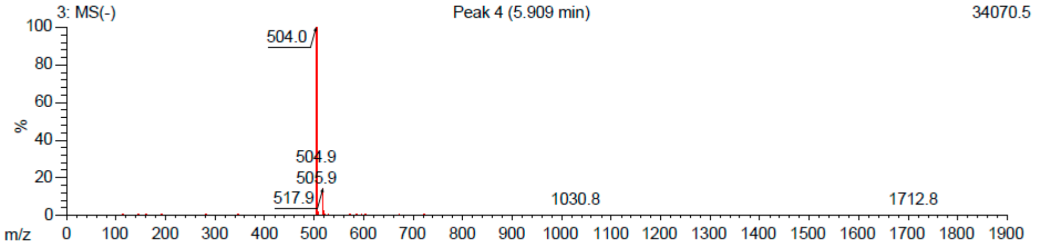

Figure S17. MS (LCMS-DAD/ESI, [M-], *m*/*z*) 3-Amino-*N*-(4-fluor-2-(trifluoromethyl)phenyl)-6-(thiophen-2-yl)-4-(trifluoromethyl)thieno[2,3-*b*]pyridine-2-carboxamide (4)

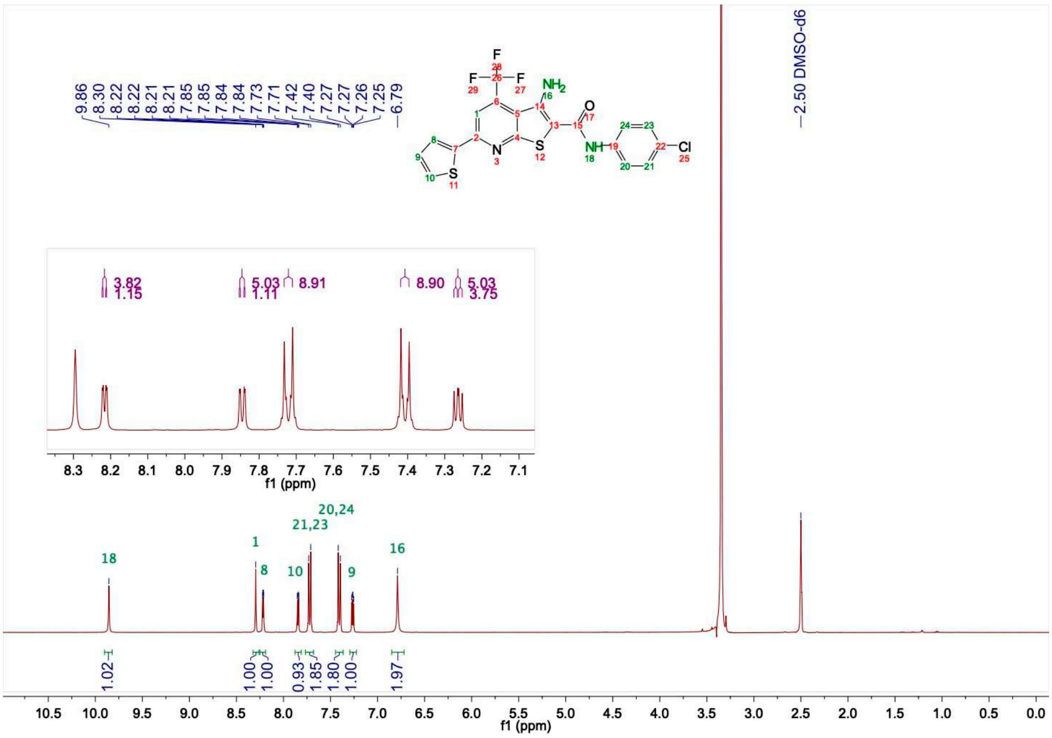

Figure S18. ^{1}H NMR (400 MHz, DMSO-d_6, δ in ppm) 3-Amino-*N*-(4-chlorophenyl)-6-(thiophen-2-yl)-4-(trifluoromethyl)thieno[2,3-*b*]pyridine-2-carboxamide (5)

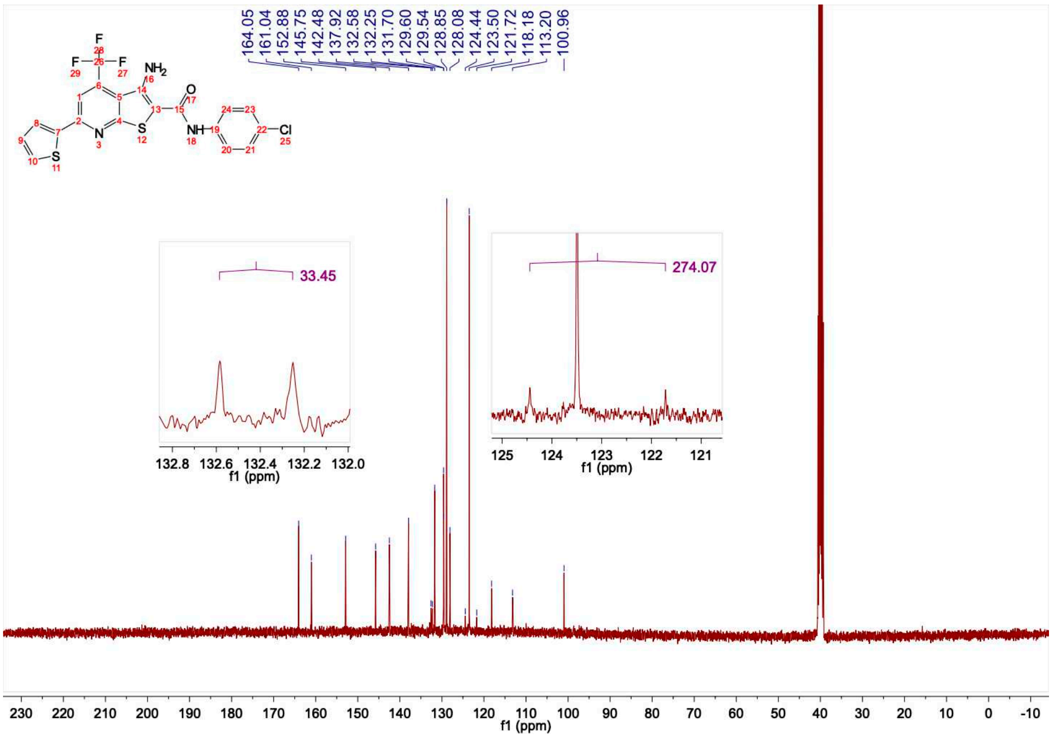

Figure S19. ^{13}C NMR (100 MHz, DMSO-d_6, δ in ppm) 3-Amino-*N*-(4-chlorophenyl)-6-(thiophen-2-yl)-4-(trifluoromethyl)thieno[2,3-*b*]pyridine-2-carboxamide (5)

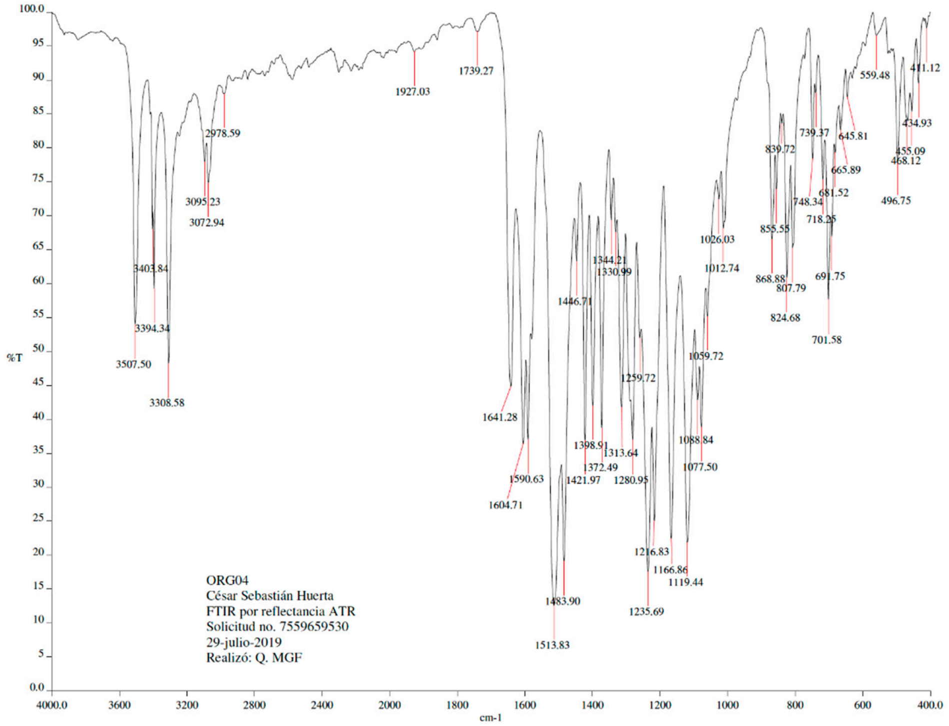

Figure S20. IR (ATR-FTIR, cm^{-1}) 3-Amino-*N*-(4-chlorophenyl)-6-(thiophen-2-yl)-4-(trifluoromethyl)thieno[2,3-*b*]pyridine-2-carboxamide (5)

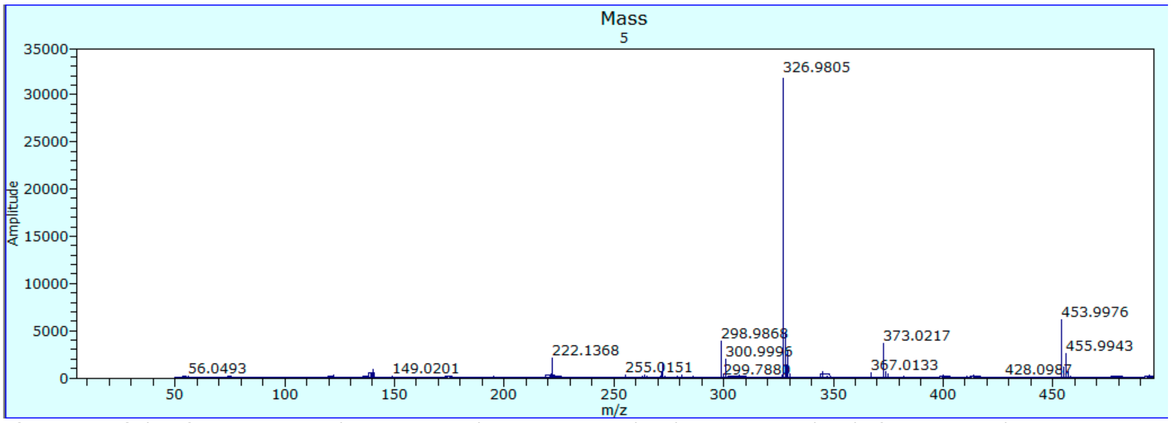

Figure S21. HRMS (APCI, $[M+H]^+$, *m/z*) 3-Amino-*N*-(4-chlorophenyl)-6-(thiophen-2-yl)-4-(trifluoromethyl)thieno[2,3-*b*]pyridine-2-carboxamide (5)

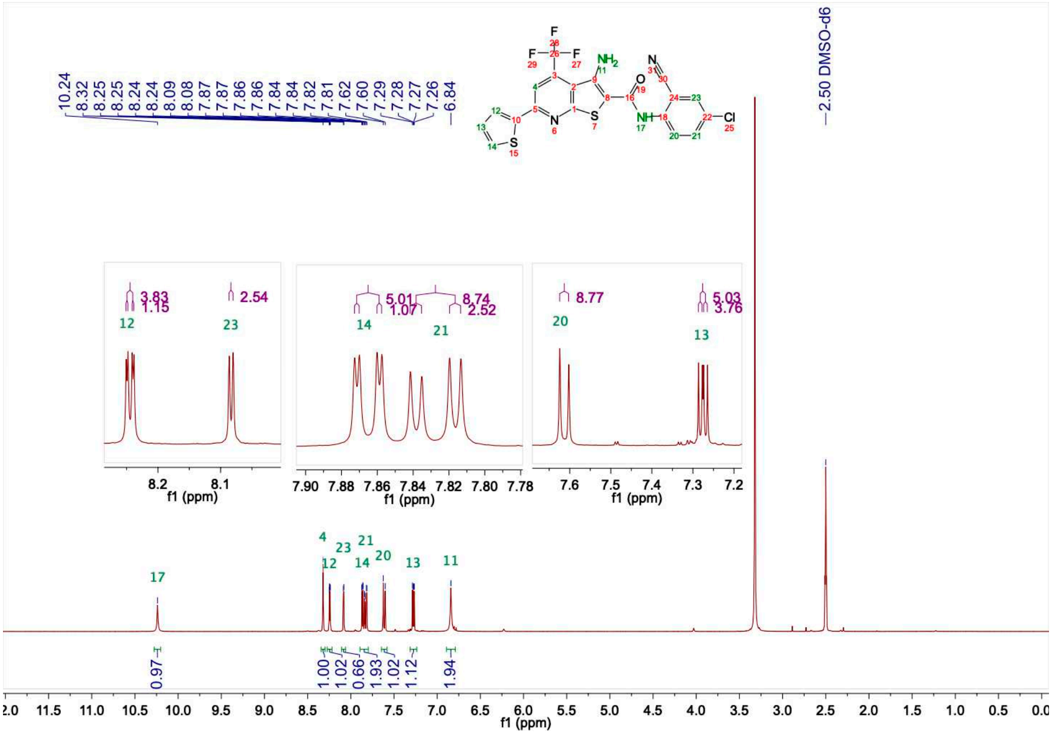

Figure S22. ^1H NMR (400 MHz, DMSO-d_6, δ in ppm) 3-Amino-*N*-(4-chloro-2-cyanophenyl)-6-(thiophen-2-yl)-4-(trifluoromethyl)thieno[2,3-*b*]pyridine-2-carboxamide (6)

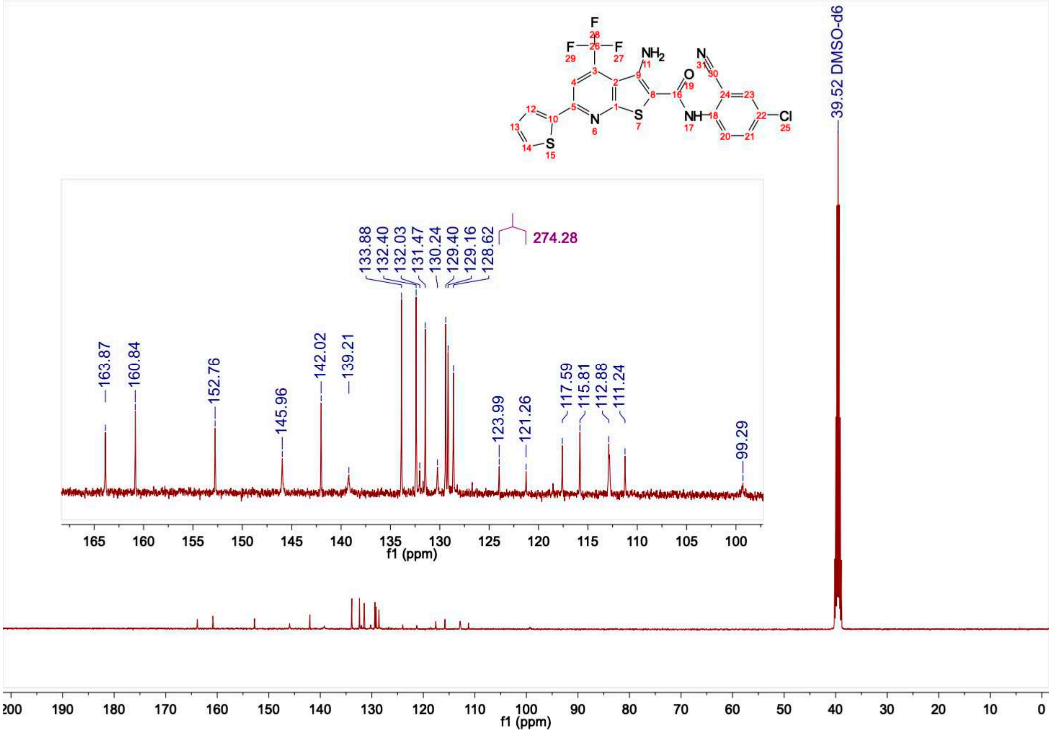

Figure S23. ^{13}C NMR (100 MHz, DMSO-d_6, δ in ppm) 3-Amino-*N*-(4-chloro-2-cyanophenyl)-6-(thiophen-2-yl)-4-(trifluoromethyl)thieno[2,3-*b*]pyridine-2-carboxamide (6)

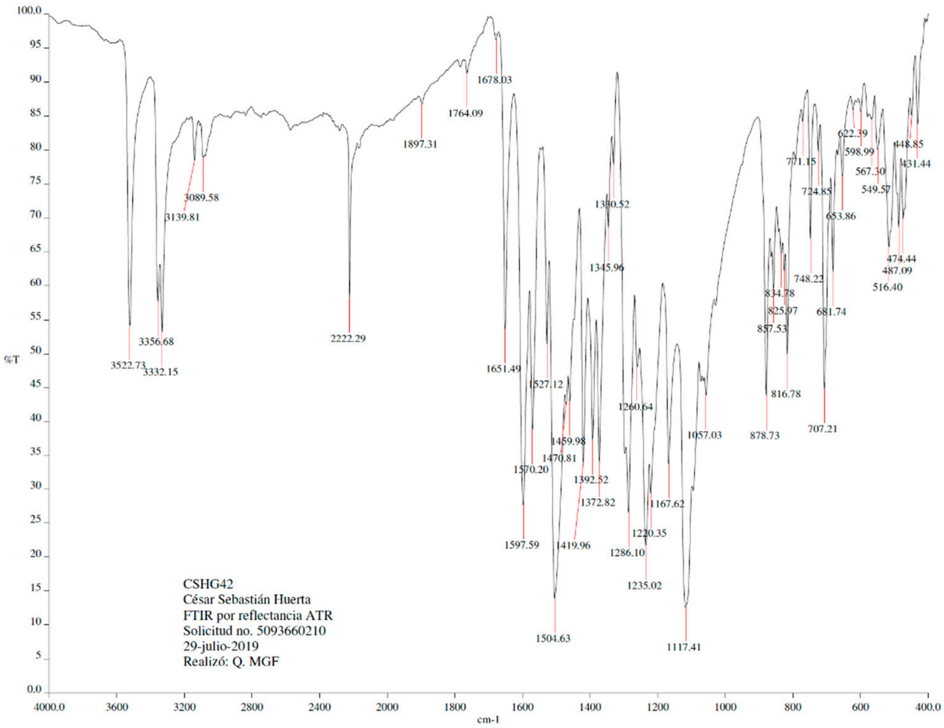

Figure S24. IR (ATR-FTIR, cm^{-1}) 3-Amino-*N*-(4-chloro-2-cyanophenyl)-6-(thiophen-2-yl)-4-(trifluoromethyl)thieno[2,3-*b*]pyridine-2-carboxamide (6)

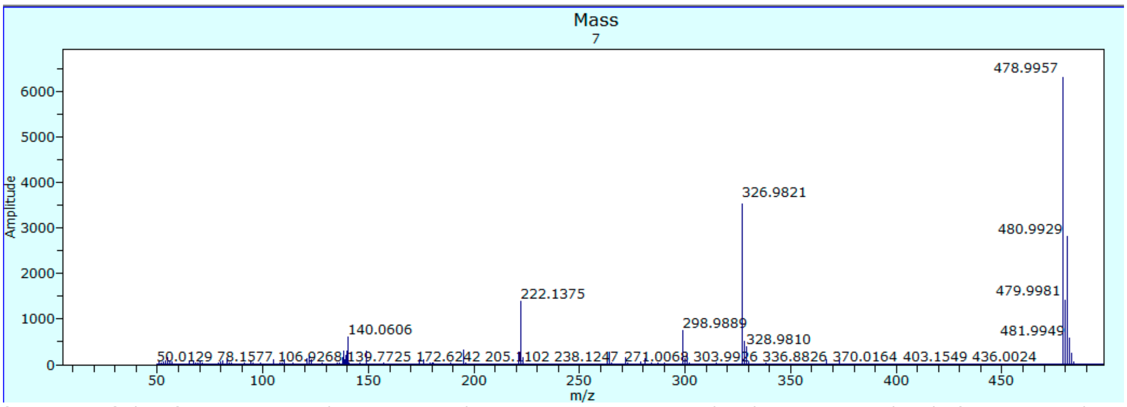

Figure S25. HRMS (APCI, $[M+H]^+$, *m/z*) 3-Amino-*N*-(4-chloro-2-cyanophenyl)-6-(thiophen-2-yl)-4-(trifluoromethyl)thieno[2,3-*b*]pyridine-2-carboxamide (6)

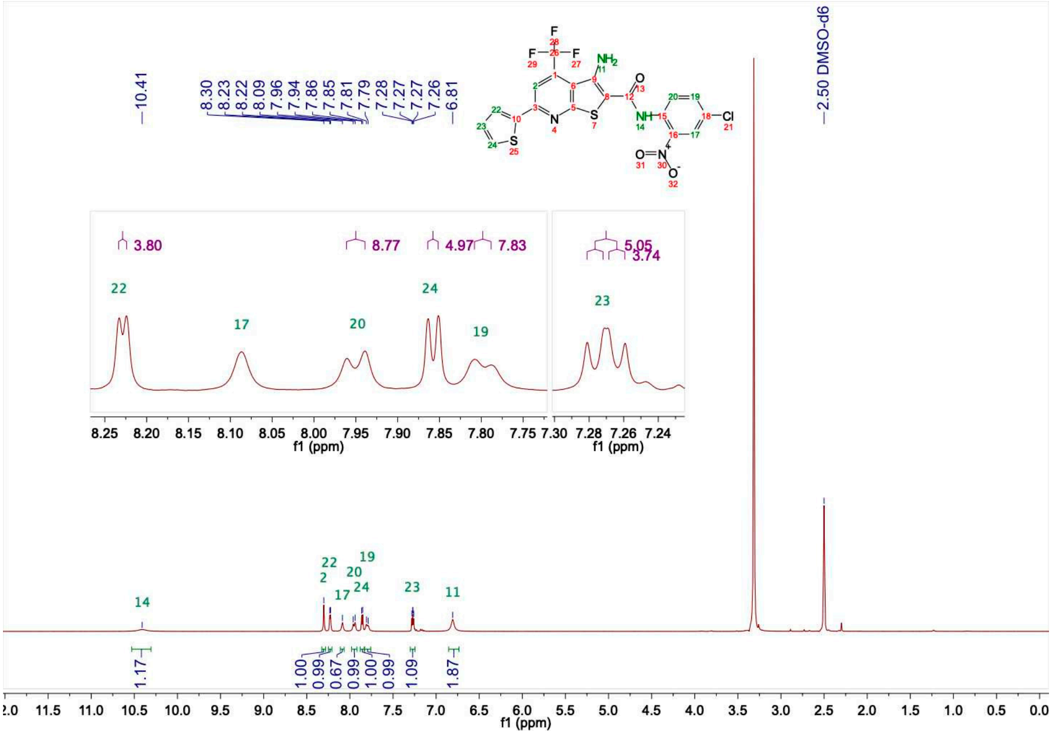

Figure S26. ^{1}H NMR (400 MHz, DMSO-d_6, δ in ppm) 3-Amino-*N*-(4-chloro-2-nitrophenyl)-6-(thiophen-2-yl)-4-(trifluoromethyl)thieno[2,3-*b*]pyridine-2-carboxamide (7)

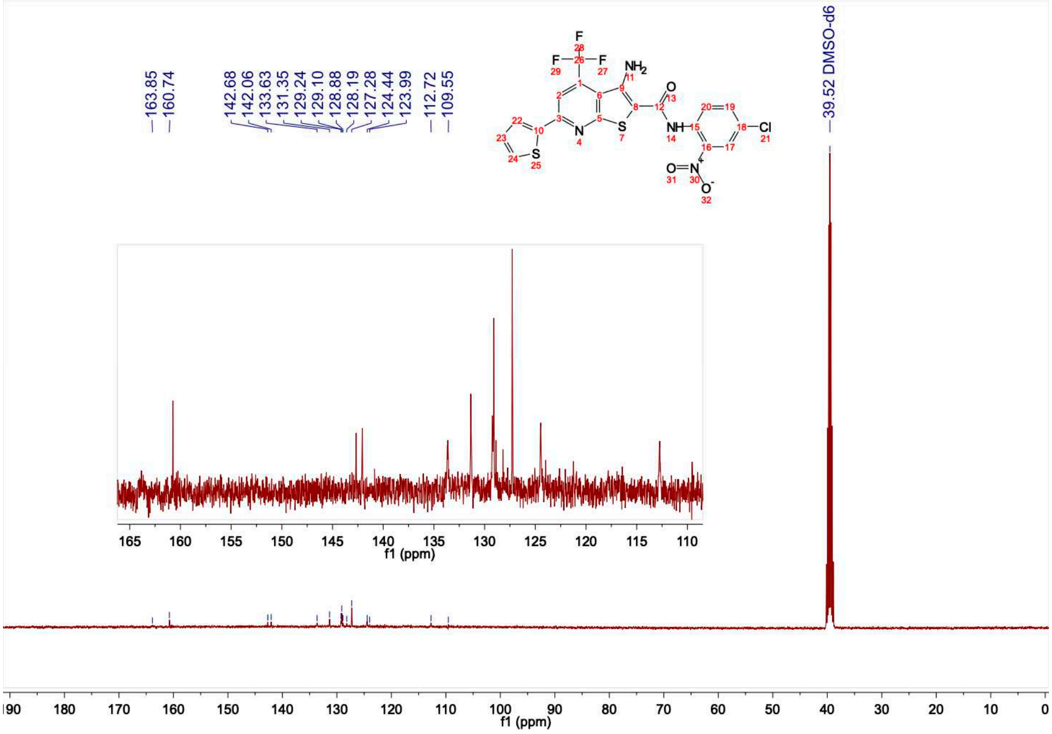

Figure S27. ^{13}C NMR (100 MHz, DMSO-*d*6, δ in ppm) 3-Amino-*N*-(4-chloro-2-nitrophenyl)-6-(thiophen-2-yl)-4-(trifluoromethyl)thieno[2,3-*b*]pyridine-2-carboxamide (7)

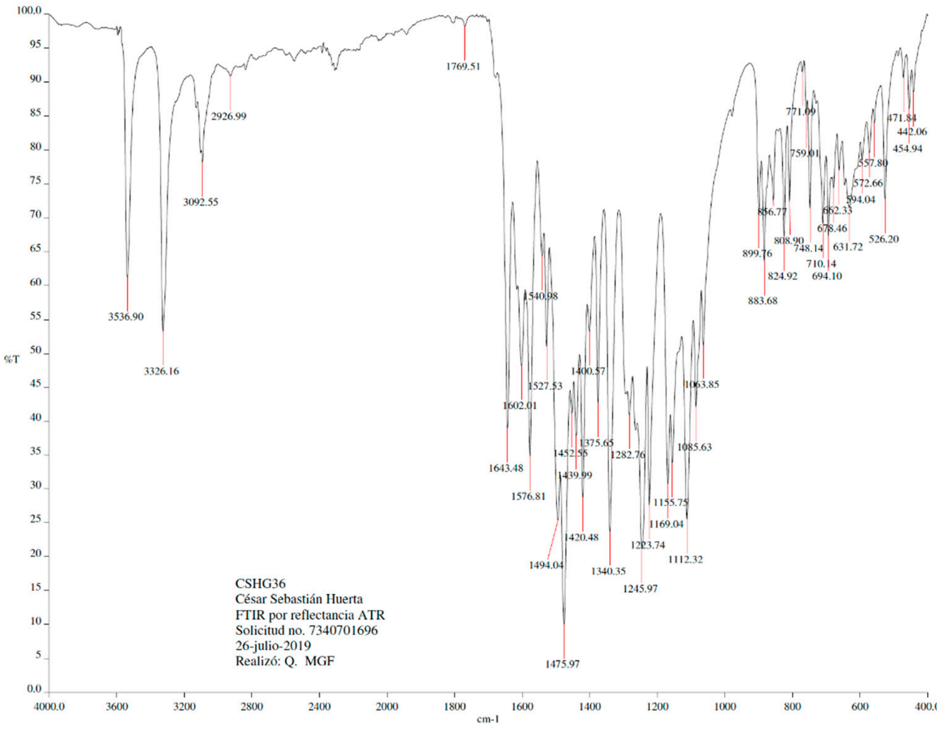

Figure S28. IR (ATR-FTIR, cm^{-1}) 3-Amino-*N*-(4-chloro-2-nitrophenyl)-6-(thiophen-2-yl)-4-(trifluoromethyl)thieno[2,3-*b*]pyridine-2-carboxamide (7)

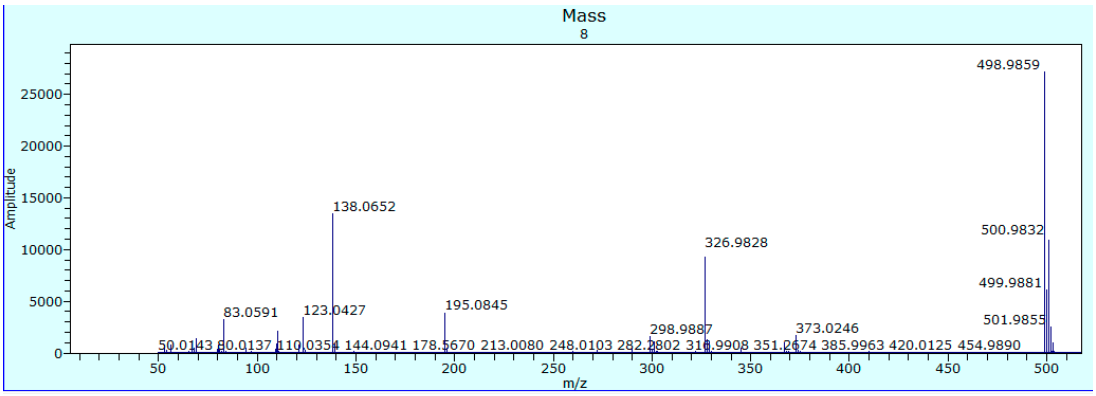

Figure S29. HRMS (APCI, [M+H]$^+$, *m/z*) 3-Amino-*N*-(4-chloro-2-nitrophenyl)-6-(thiophen-2-yl)-4-(trifluoromethyl)thieno[2,3-*b*]pyridine-2-carboxamide (7)

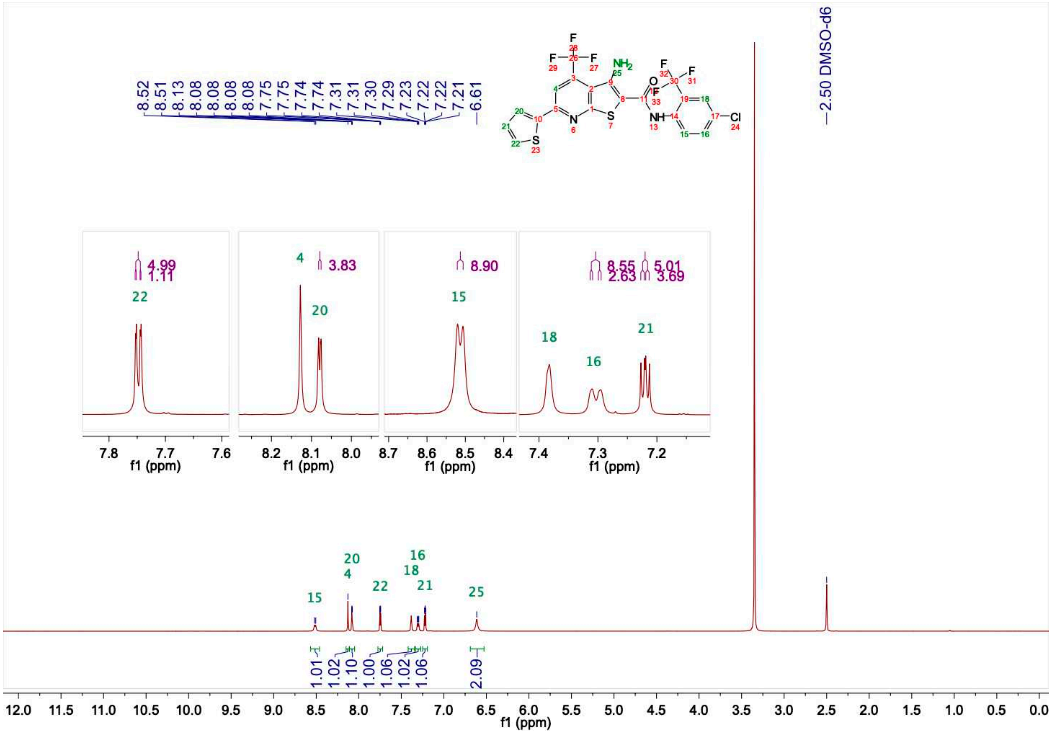

Figure S30. ^{1}H NMR (600 MHz, DMSO-d_6, δ in ppm) 3-Amino-*N*-(4-chloro-2-(trifluoromethyl)phenyl)-6-(thiophen-2-yl)-4-(trifluoromethyl)thieno[2,3-*b*]pyridine-2-carboxamide (8)

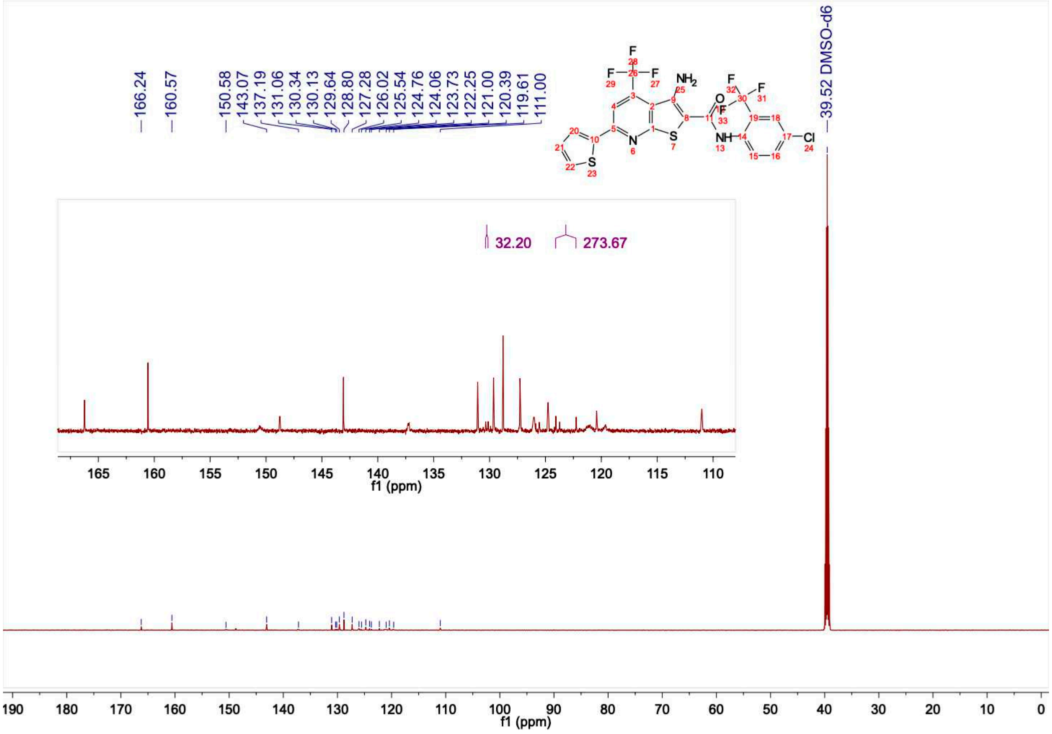

Figure S31. ^{13}C NMR (150 MHz, DMSO-d_6, δ in ppm) 3-Amino-*N*-(4-chloro-2-(trifluoromethyl)phenyl)-6-(thiophen-2-yl)-4-(trifluoromethyl)thieno[2,3-*b*]pyridine-2-carboxamide (8)

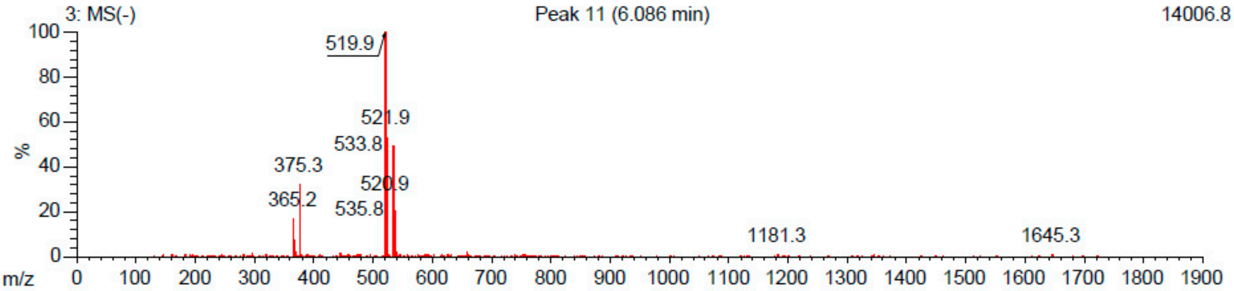

Figure S32. MS (LCMS-DAD/ESI, [M$^-$], *m*/*z*) 3-Amino-*N*-(4-chloro-2-(trifluoromethyl)phenyl)-6-(thiophen-2-yl)-4-(trifluoromethyl)thieno[2,3-*b*]pyridine-2-carboxamide (8)

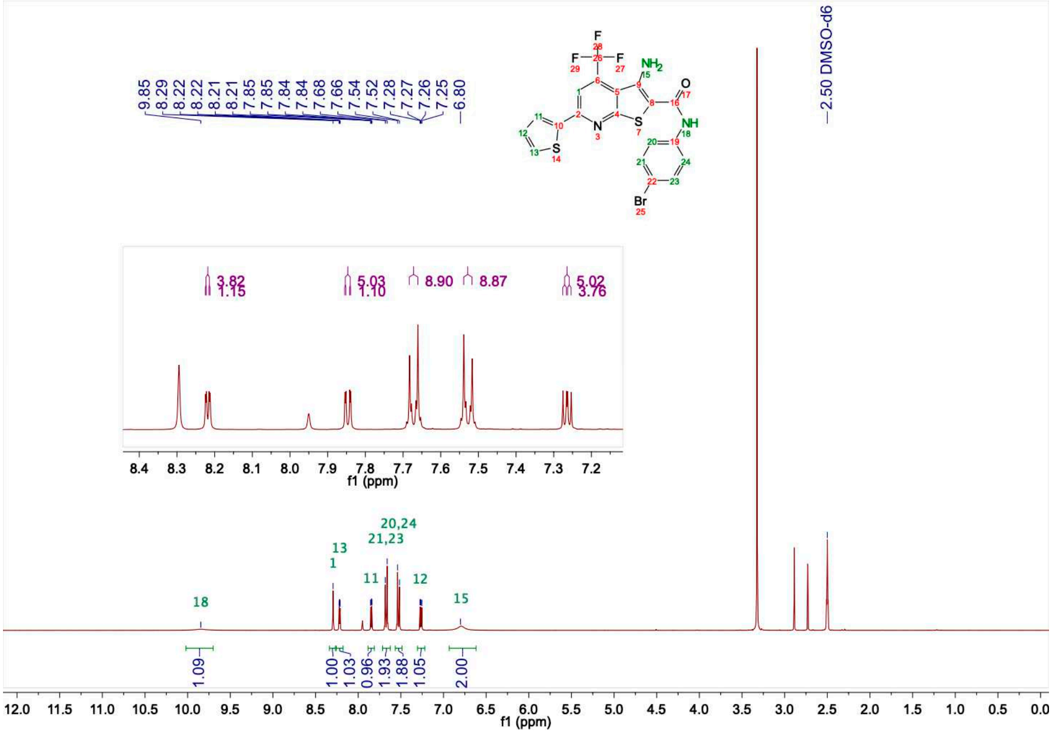

Figure S33. ^{1}H NMR (400 MHz, DMSO-d_6, δ in ppm) 3-Amino-*N*-(4-bromophenyl)-6-(thiophen-2-yl)-4-(trifluoromethyl)thieno[2,3-*b*]pyridine-2-carboxamide (9)

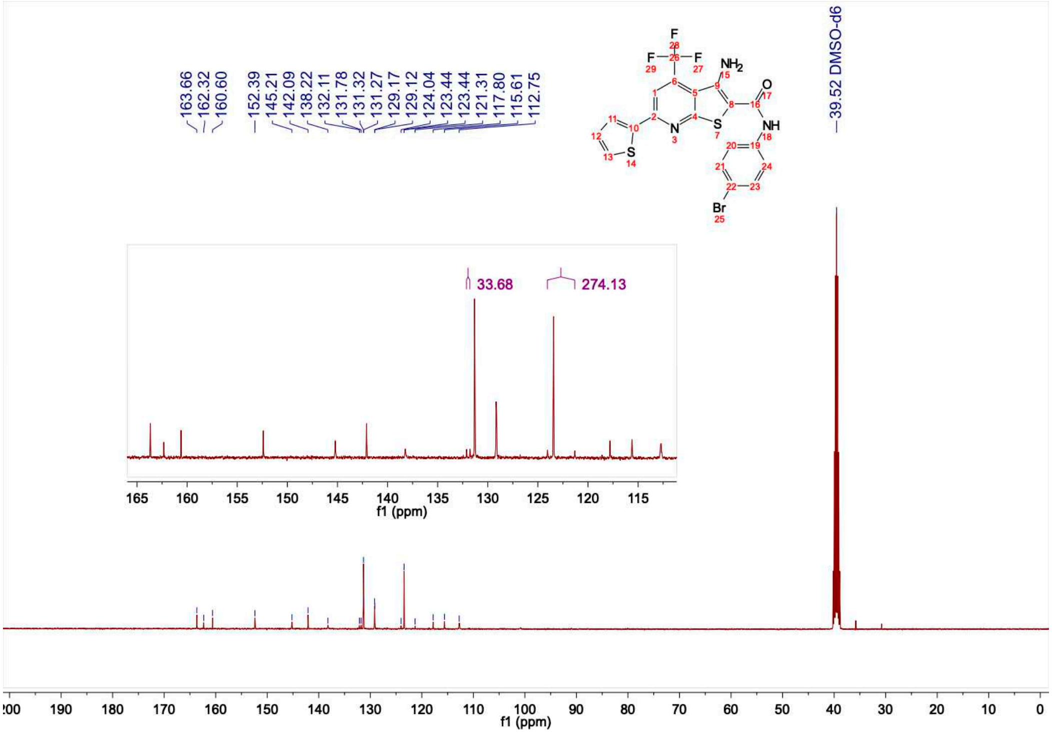

Figure S34. ^{13}C NMR (100 MHz, DMSO-d_6, δ in ppm) 3-Amino-*N*-(4-bromophenyl)-6-(thiophen-2-yl)-4-(trifluoromethyl)thieno[2,3-*b*]pyridine-2-carboxamide (9)

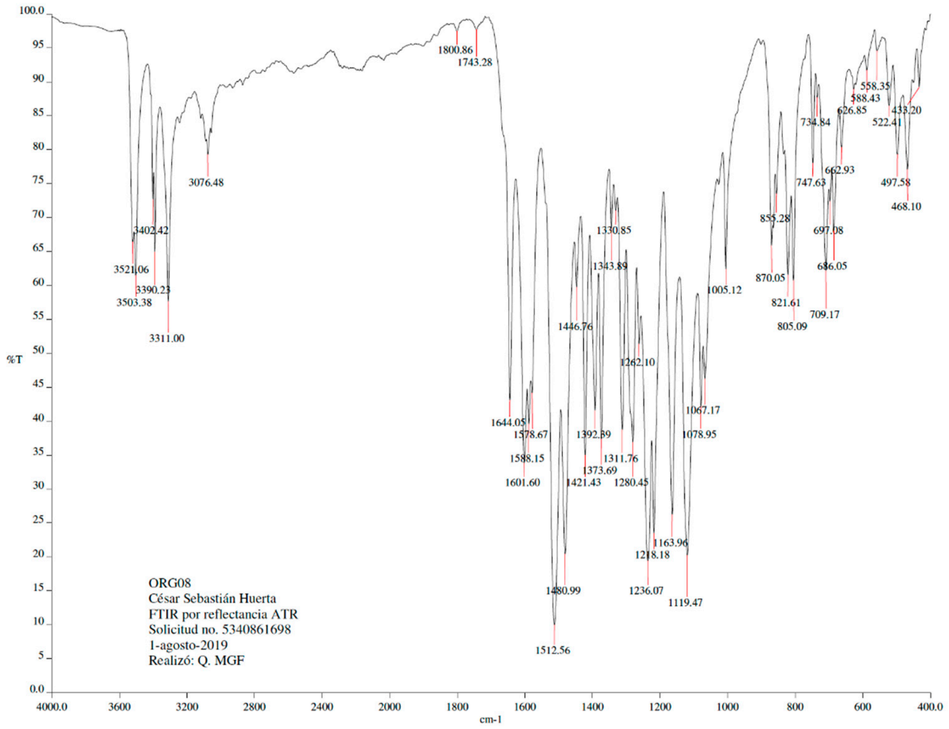

Figure S35. IR (ATR-FTIR, cm^{-1}) 3-Amino-*N*-(4-bromophenyl)-6-(thiophen-2-yl)-4-(trifluoromethyl)thieno[2,3-*b*]pyridine-2-carboxamide (9)

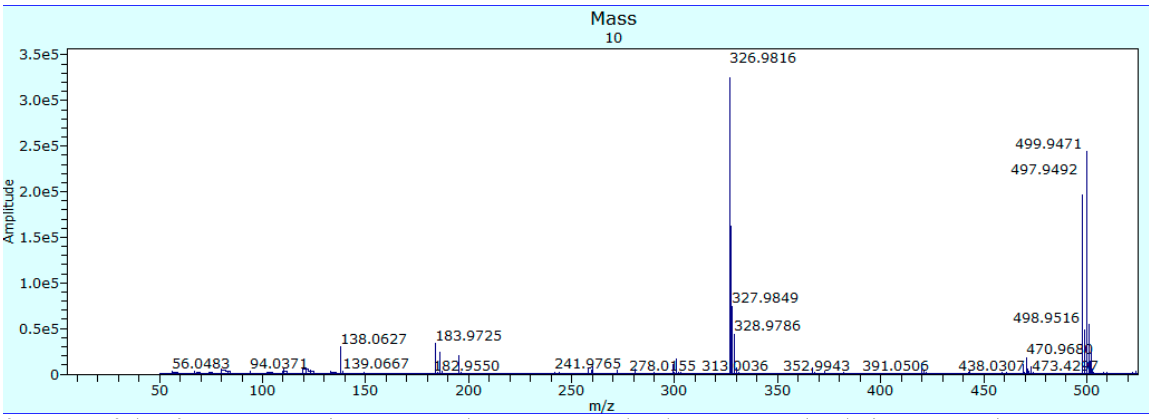

Figure S36. HRMS (APCI, [M+H]$^+$, *m/z*) 3-Amino-*N*-(4-bromophenyl)-6-(thiophen-2-yl)-4-(trifluoromethyl)thieno[2,3-*b*]pyridine-2-carboxamide (9)

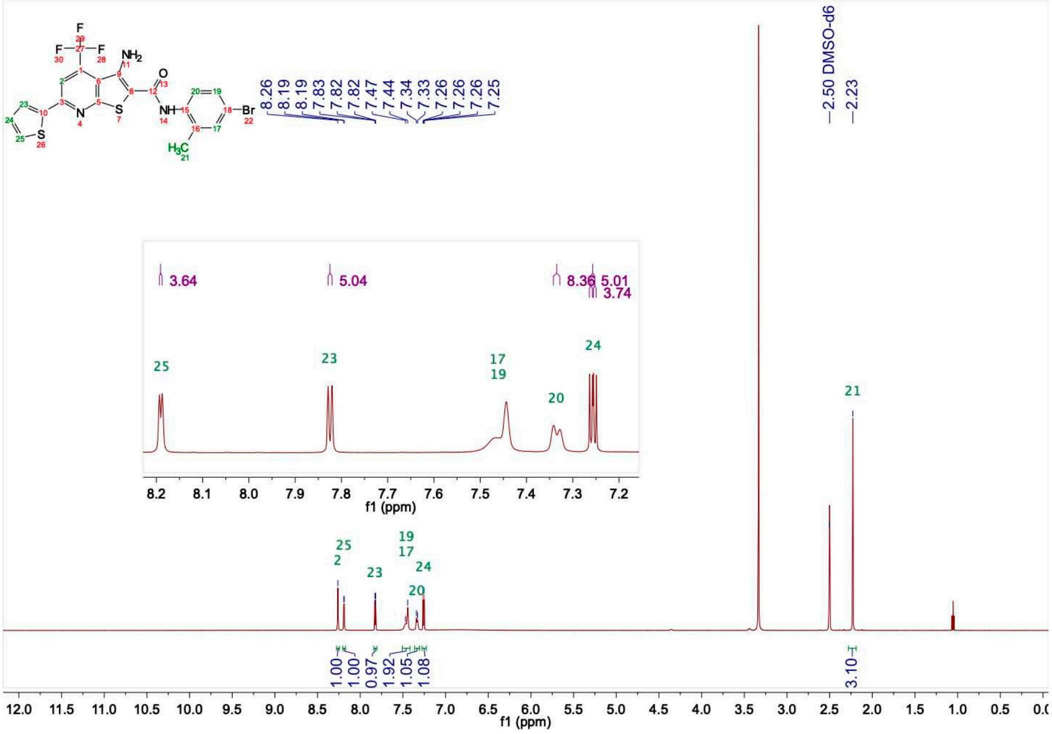

Figure S37. ^{1}H NMR (600 MHz, DMSO-d_6, δ in ppm) 3-Amino-*N*-(4-bromo-2-methylphenyl)-6-(thiophen-2-yl)-4-(trifluoromethyl)thieno[2,3-*b*]pyridine-2-carboxamide (10)

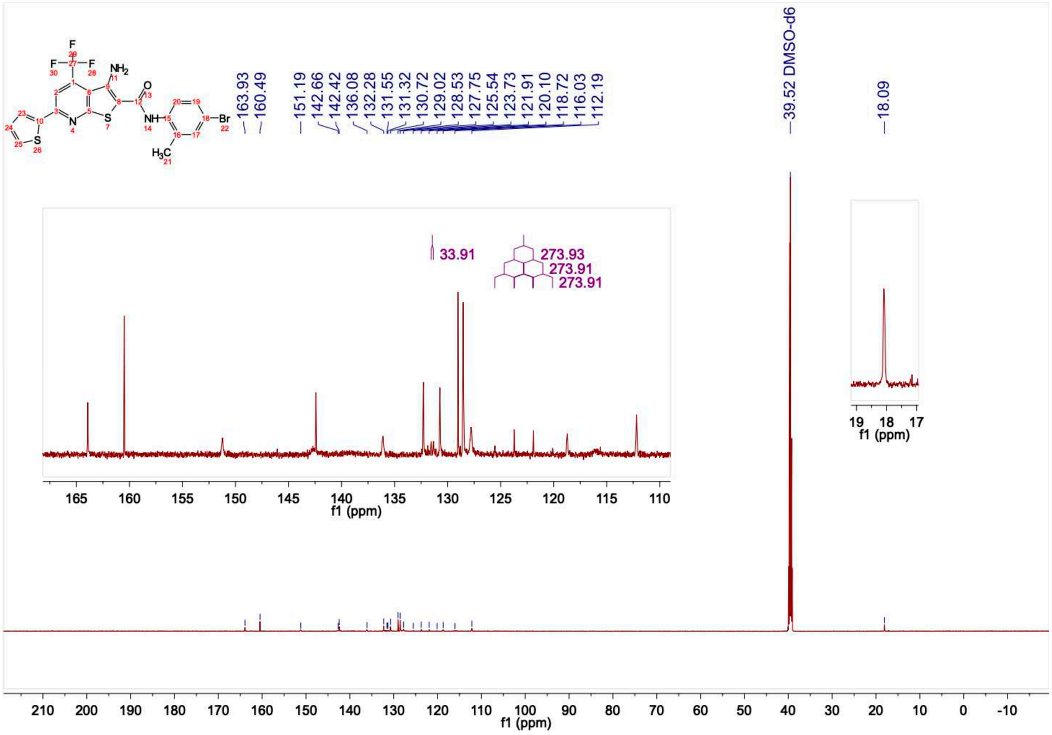

Figure S38. ^{13}C NMR (150 MHz, DMSO-d_6, δ in ppm) 3-Amino-*N*-(4-bromo-2-methylphenyl)-6-(thiophen-2-yl)-4-(trifluoromethyl)thieno[2,3-*b*]pyridine-2-carboxamide (10)

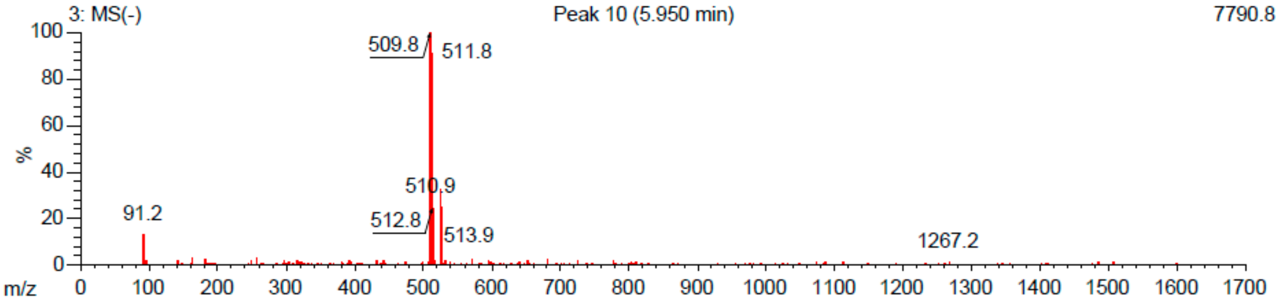

Figure S39. MS (LCMS-DAD/ESI, [M-], *m/z*) 3-Amino-*N*-(4-bromo-2-methylphenyl)-6-(thiophen-2-yl)-4-(trifluoromethyl)thieno[2,3-*b*]pyridine-2-carboxamide (10)

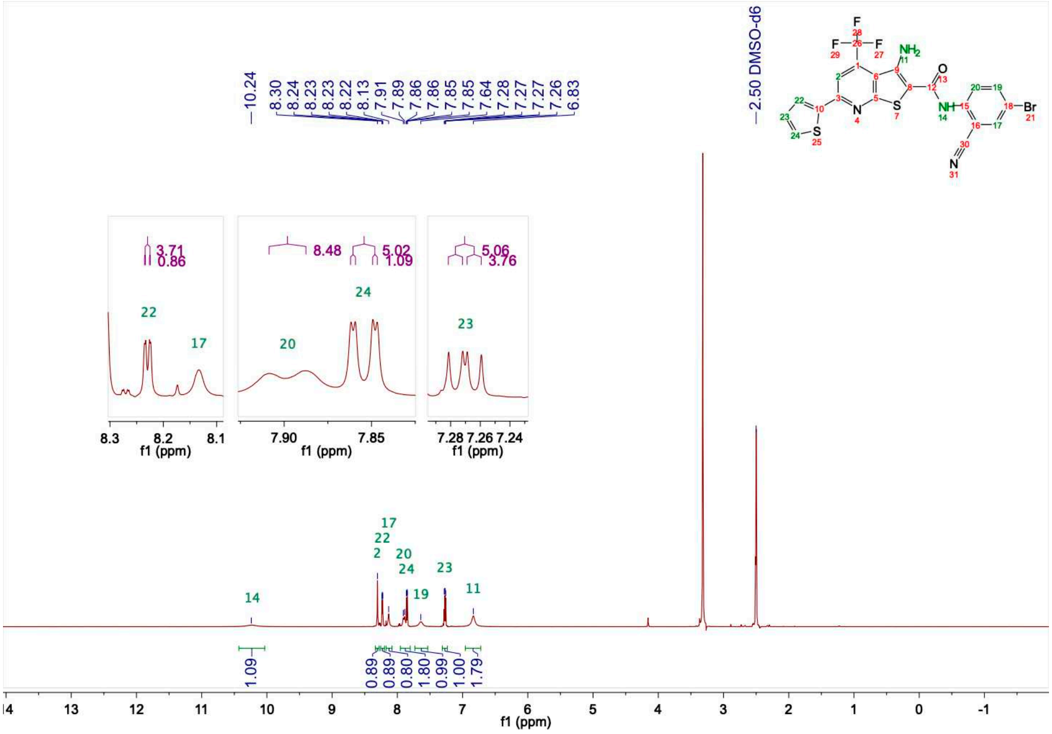

Figure S40. ^{1}H NMR (400 MHz, DMSO-d_6, δ in ppm) 3-Amino-*N*-(4-bromo-2-cyanophenyl)-6-(thiophen-2-yl)-4-(trifluoromethyl)thieno[2,3-*b*]pyridine-2-carboxamide (11)

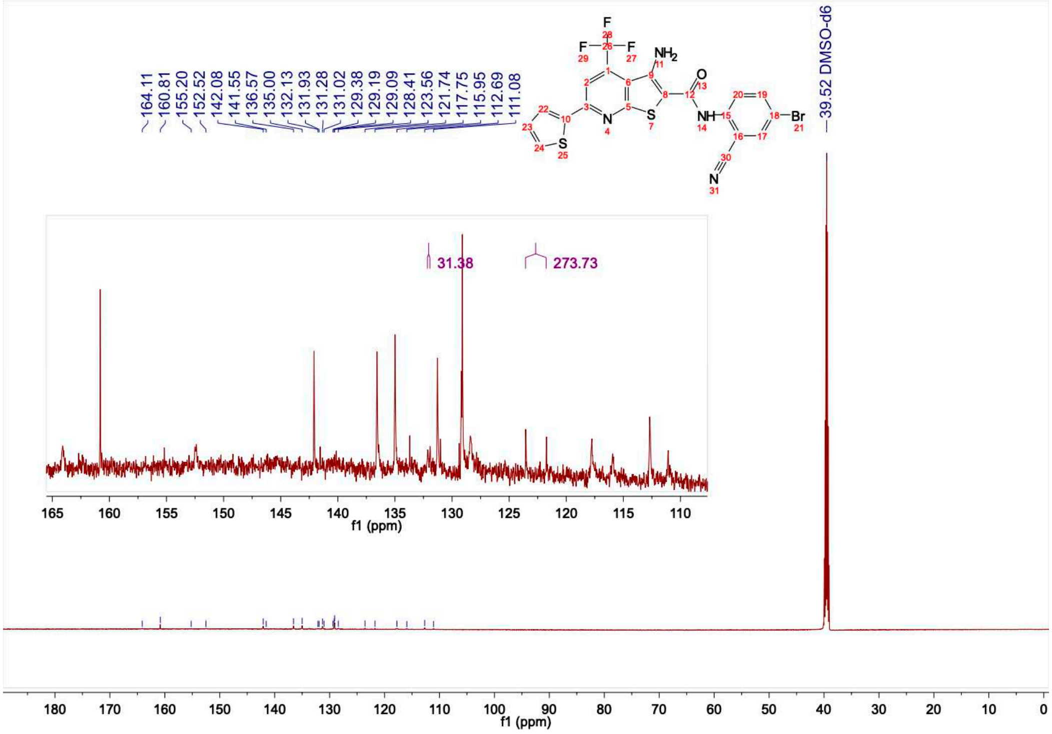

Figure S41. ^{13}C NMR (400 MHz, DMSO-d_6, δ in ppm) 3-Amino-*N*-(4-bromo-2-cyanophenyl)-6-(thiophen-2-yl)-4-(trifluoromethyl)thieno[2,3-*b*]pyridine-2-carboxamide (11)

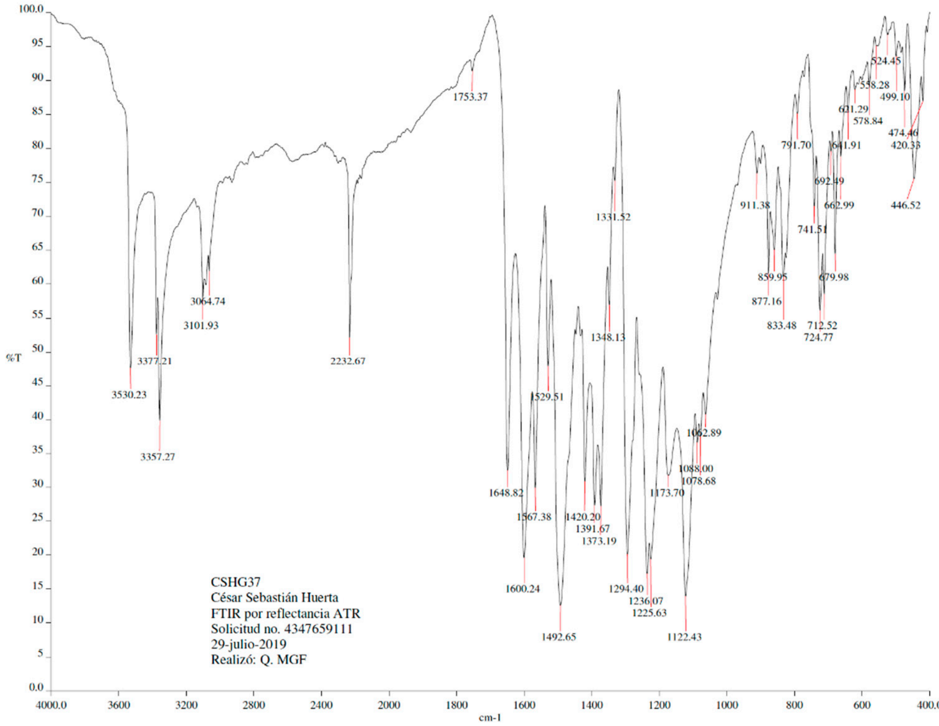

Figure S42. IR (ATR-FTIR, cm^{-1}) 3-Amino-*N*-(4-bromo-2-cyanophenyl)-6-(thiophen-2-yl)-4-(trifluoromethyl)thieno[2,3-*b*]pyridine-2-carboxamide (11)

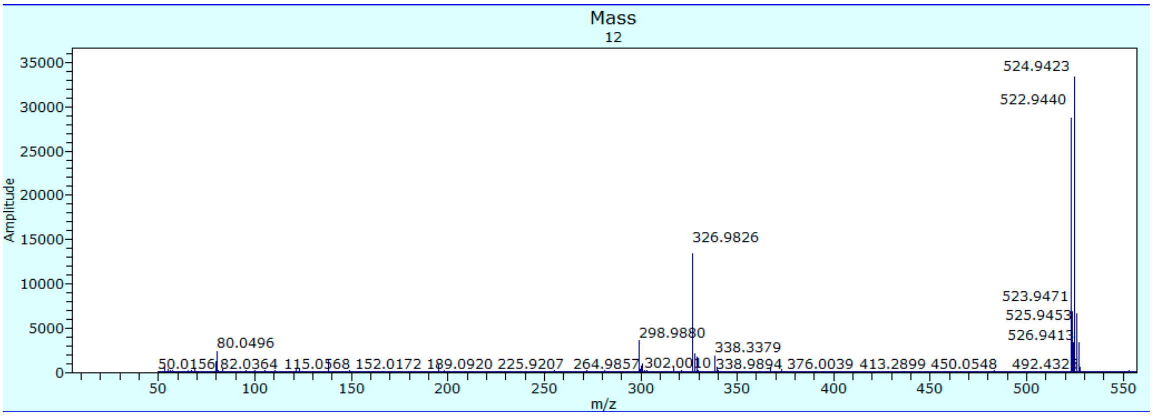

Figure S43. HRMS (APCI, [M+H]+, *m/z*) 3-Amino-*N*-(4-bromo-2-cyanophenyl)-6-(thiophen-2-yl)-4-(trifluoromethyl)thieno[2,3-*b*]pyridine-2-carboxamide (11)

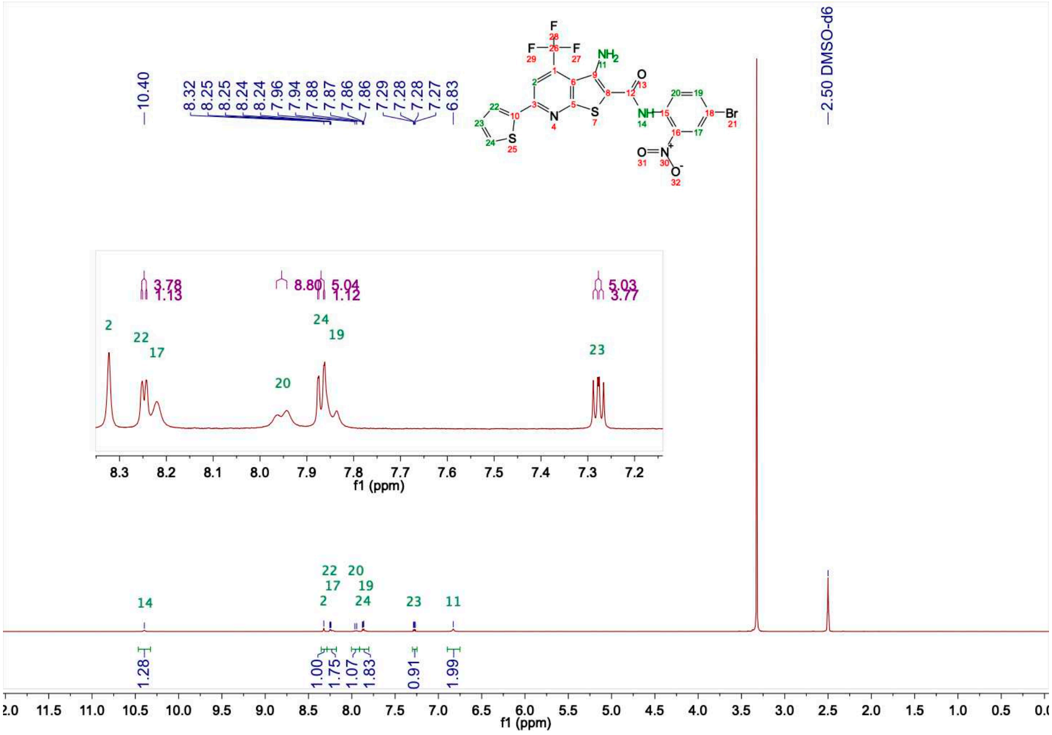

Figure S44. ^{1}H NMR (400 MHz, DMSO-d_6, δ in ppm) 3-Amino-*N*-(4-bromo-2-nitrophenyl)-6-(thiophen-2-yl)-4-(trifluoromethyl)thieno[2,3-*b*]pyridine-2-carboxamide (12)

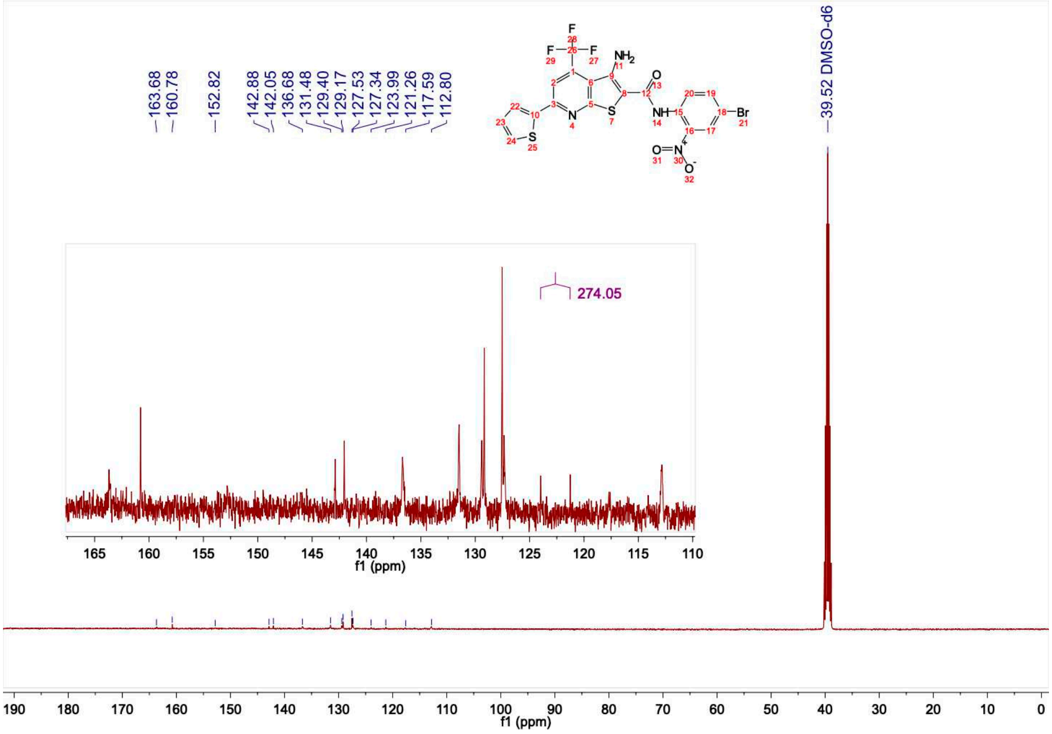

Figure S45. ^{13}C NMR (100 MHz, DMSO-d_6, δ in ppm) 3-Amino-*N*-(4-bromo-2-nitrophenyl)-6-(thiophen-2-yl)-4-(trifluoromethyl)thieno[2,3-*b*]pyridine-2-carboxamide (12)

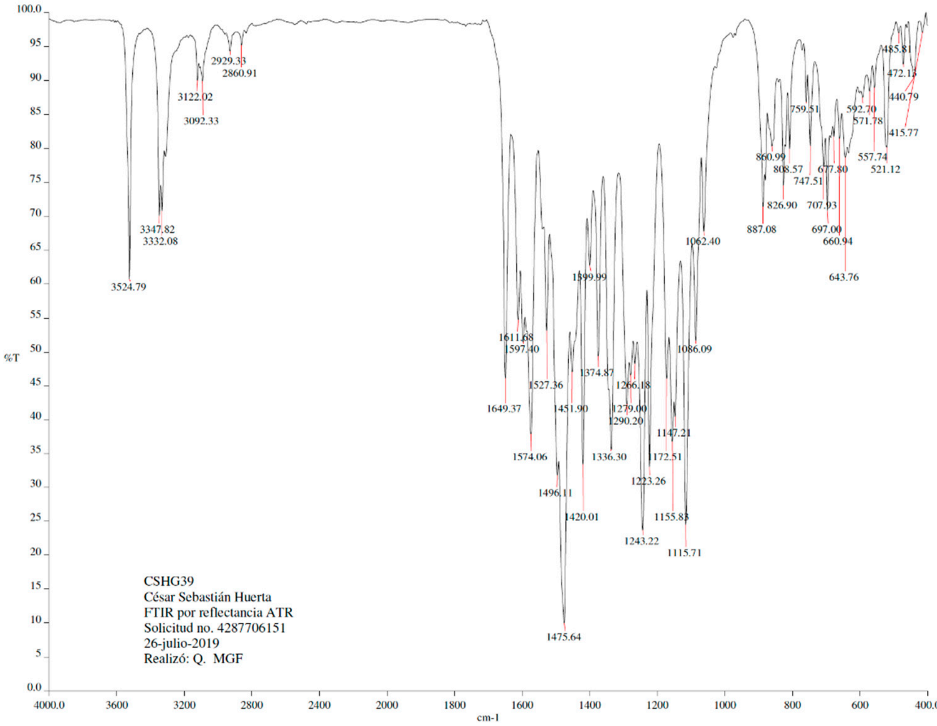

Figure S46. IR (ATR-FTIR, cm^{-1}) 3-Amino-*N*-(4-bromo-2-nitrophenyl)-6-(thiophen-2-yl)-4-(trifluoromethyl)thieno[2,3-*b*]pyridine-2-carboxamide (12)

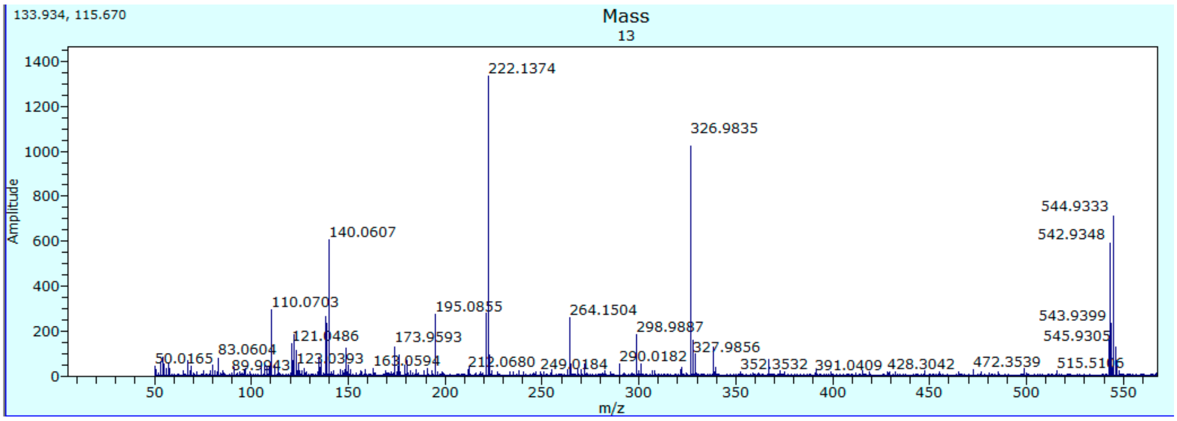

Figure S47. HRMS (APCI, [M+H]$^+$, *m/z*) 3-Amino-*N*-(4-bromo-2-nitrophenyl)-6-(thiophen-2-yl)-4-(trifluoromethyl)thieno[2,3-*b*]pyridine-2-carboxamide (12)

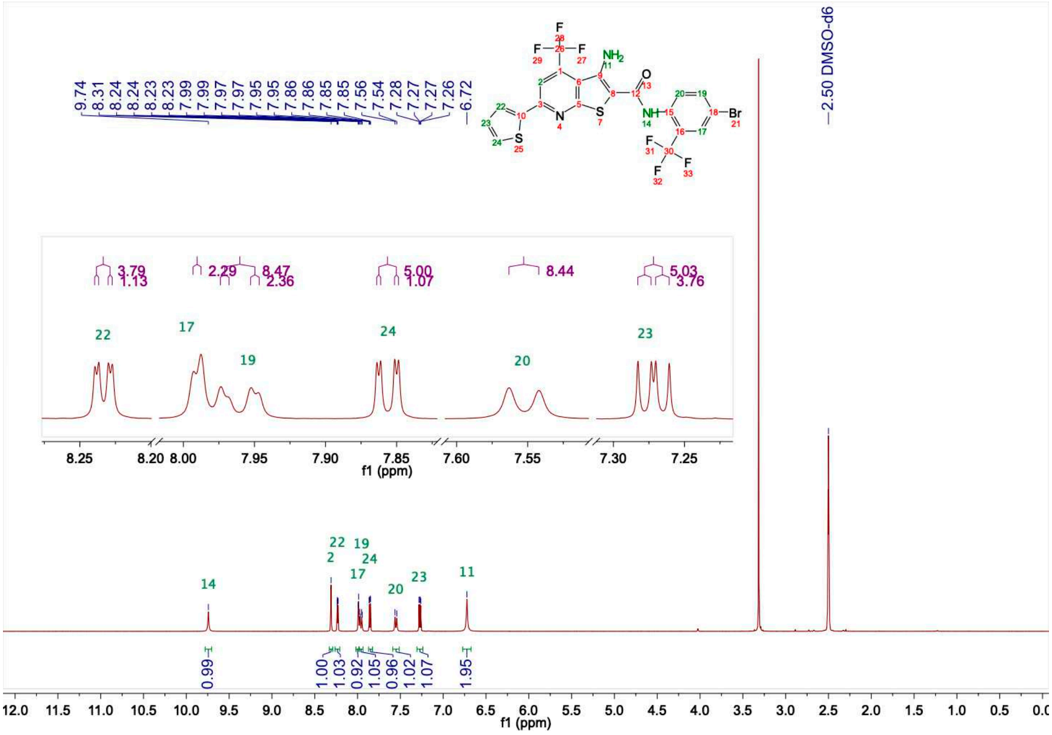

Figure S48. ^{1}H NMR (400 MHz, DMSO-d_6, δ in ppm) 3-Amino-*N*-(4-bromo-2-(trifluoromethyl)phenyl)-6-(thiophen-2-yl)-4-(trifluoromethyl)thieno[2,3-*b*]pyridine-2-carboxamide (13)

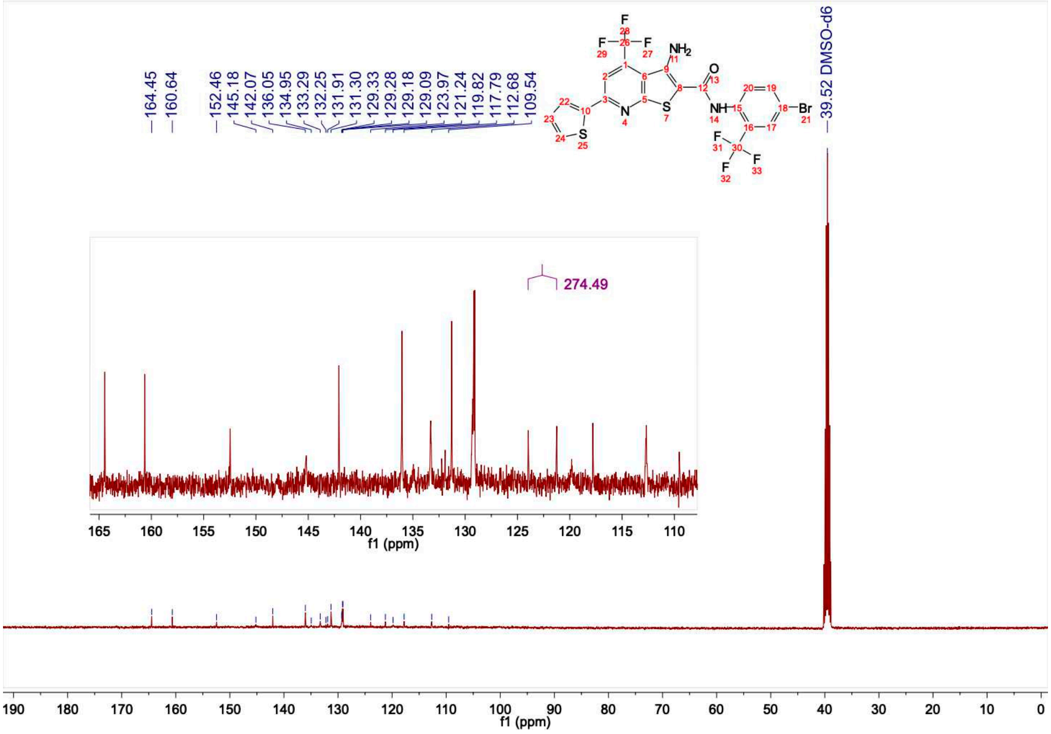

Figure S49. ^{13}C NMR (100 MHz, DMSO-d_6, δ in ppm) 3-Amino-*N*-(4-bromo-2-(trifluoromethyl)phenyl)-6-(thiophen-2-yl)-4-(trifluoromethyl)thieno[2,3-*b*]pyridine-2-carboxamide (13)

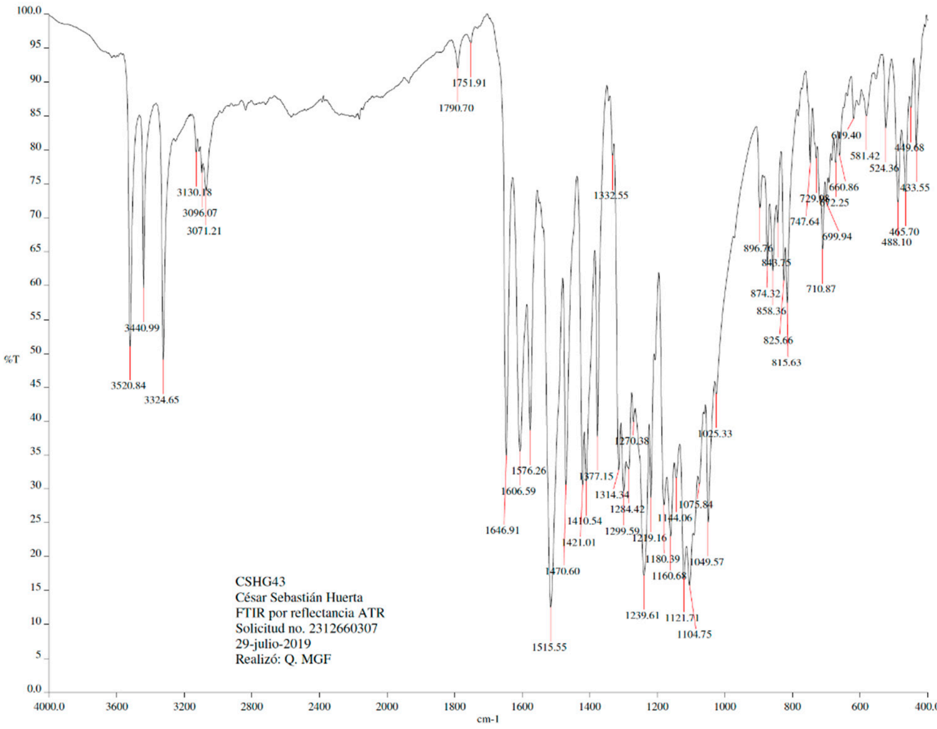

Figure S50. IR (ATR-FTIR, cm^{-1}) 3-Amino-*N*-(4-bromo-2-(trifluoromethyl)phenyl)-6-(thiophen-2-yl)-4-(trifluoromethyl)thieno[2,3-*b*]pyridine-2-carboxamide (13)

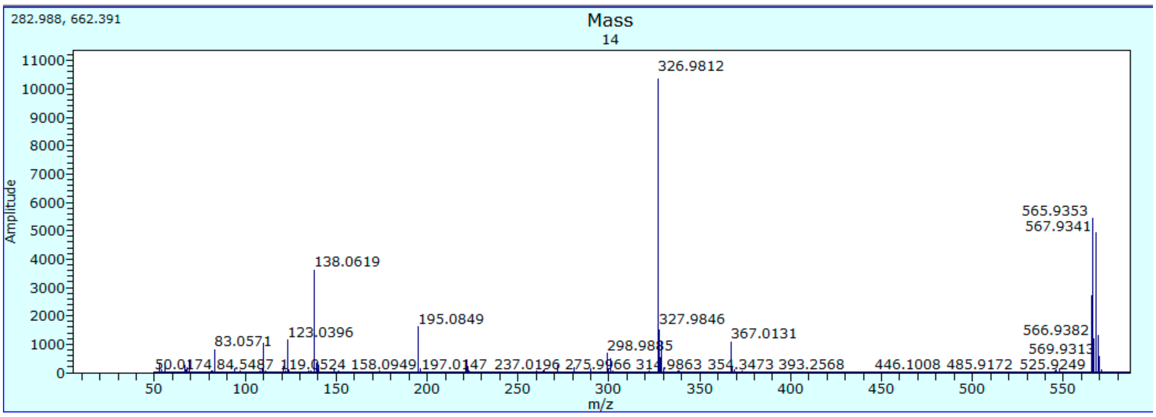

Figure S51. HRMS (APCI, [M+H]$^+$, *m/z*)3-Amino-*N*-(4-bromo-2-(trifluoromethyl)phenyl)-6-(thiophen-2-yl)-4-(trifluoromethyl)thieno[2,3-*b*]pyridine-2-carboxamide (13)

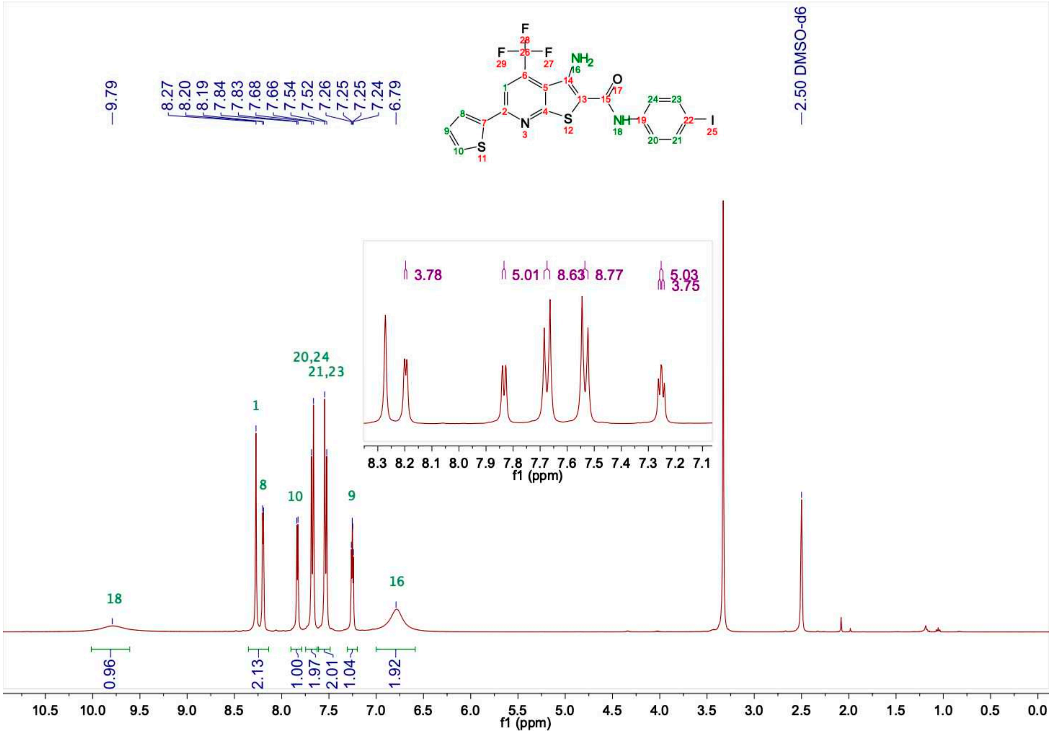

Figure S52. ^{1}H NMR (400 MHz, DMSO-d_6, δ in ppm) 3-Amino-*N*-(4-iodophenyl)-6-(thiophen-2-yl)-4-(trifluoromethyl)-thieno[2,3-*b*]pyridine-2-carboxamide (14)

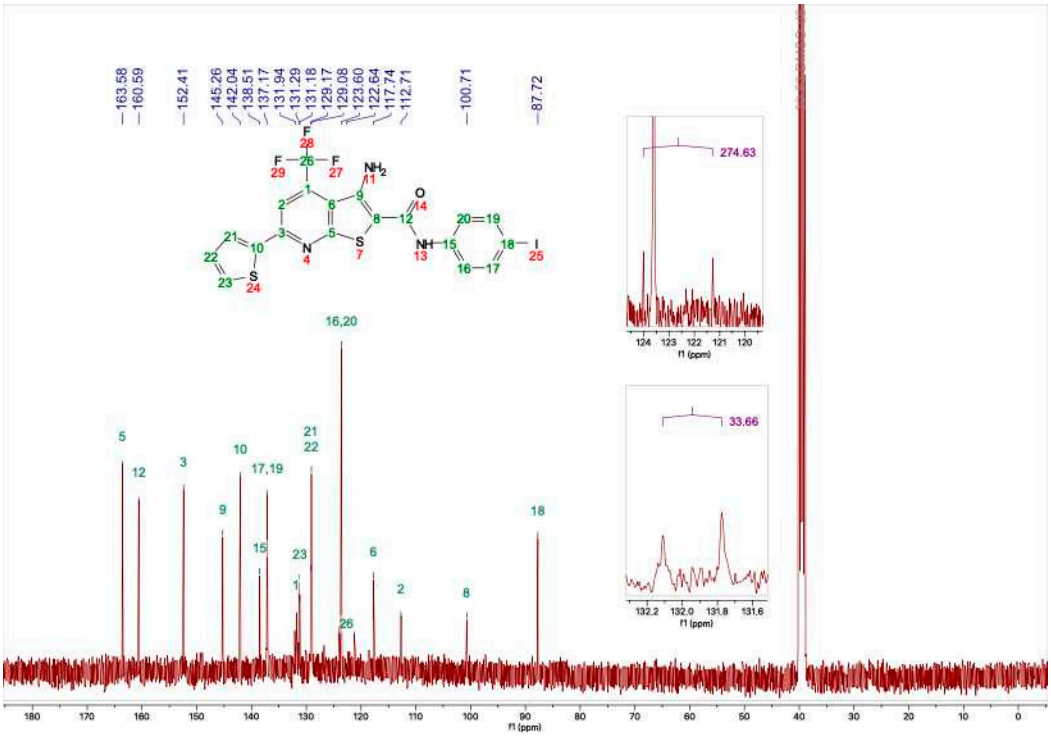

Figure S53. ^{13}C NMR (100 MHz, DMSO-d_6, δ in ppm) 3-Amino-*N*-(4-iodophenyl)-6-(thiophen-2-yl)-4-(trifluoromethyl)-thieno[2,3-*b*]pyridine-2-carboxamide (14)

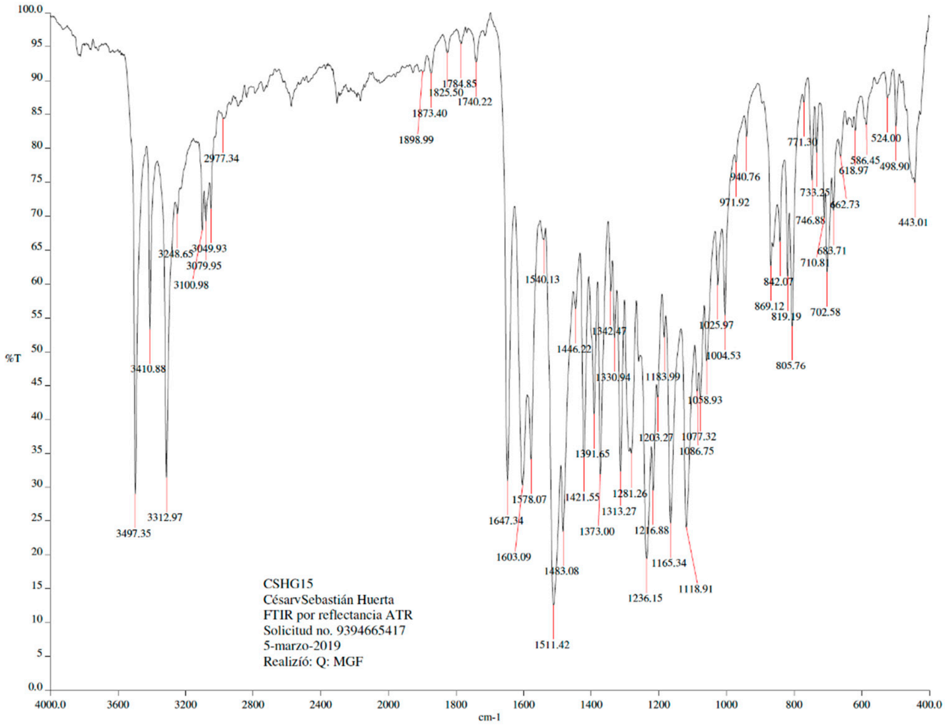

Figure S54. IR (ATR-FTIR, cm^{-1}) 3-Amino-*N*-(4-iodophenyl)-6-(thiophen-2-yl)-4-(trifluoromethyl)-thieno[2,3-*b*]pyridine-2-carboxamide (14)

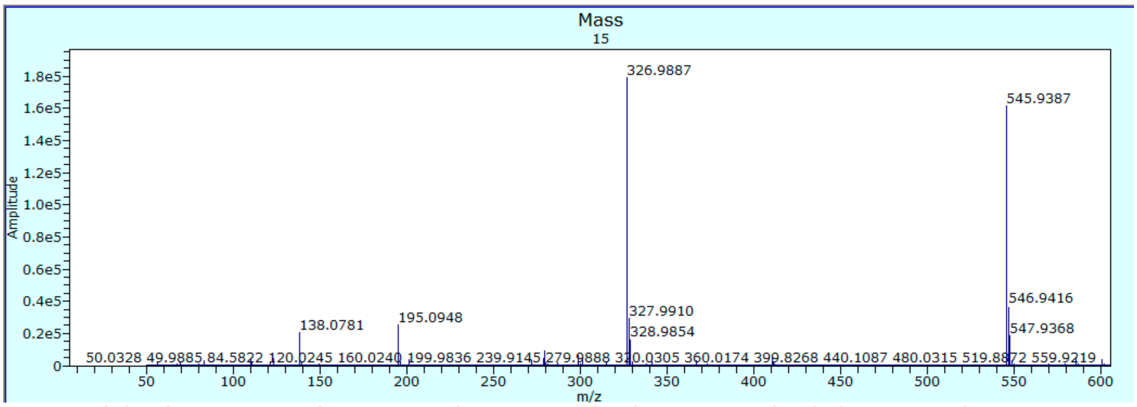

Figure S55. HRMS (APCI, [M+H]$^+$, *m/z*) 3-Amino-*N*-(4-iodophenyl)-6-(thiophen-2-yl)-4-(trifluoromethyl)-thieno[2,3-*b*]pyridine-2-carboxamide (14)

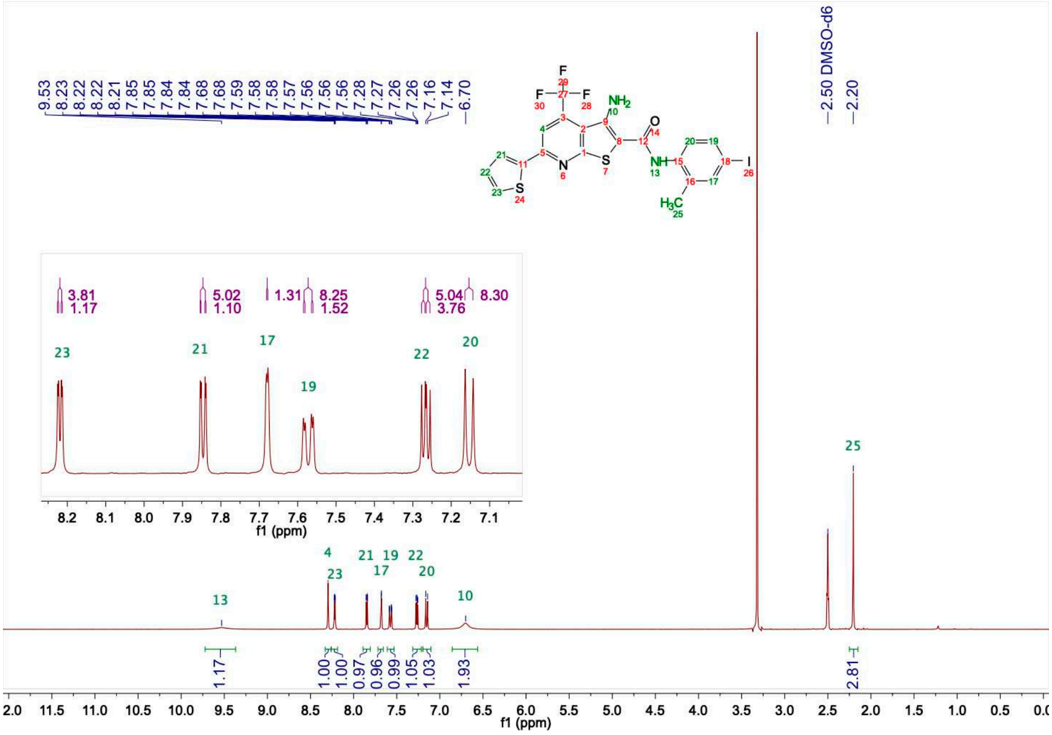

Figure S56. ^{1}H NMR (400 MHz, DMSO-d_6, δ in ppm) 3-Amino-*N*-(4-iodo-2-methylphenyl)-6-(thiophen-2-yl)-4-(trifluoromethyl)thieno[2,3-*b*]pyridine-2-carboxamide (15)

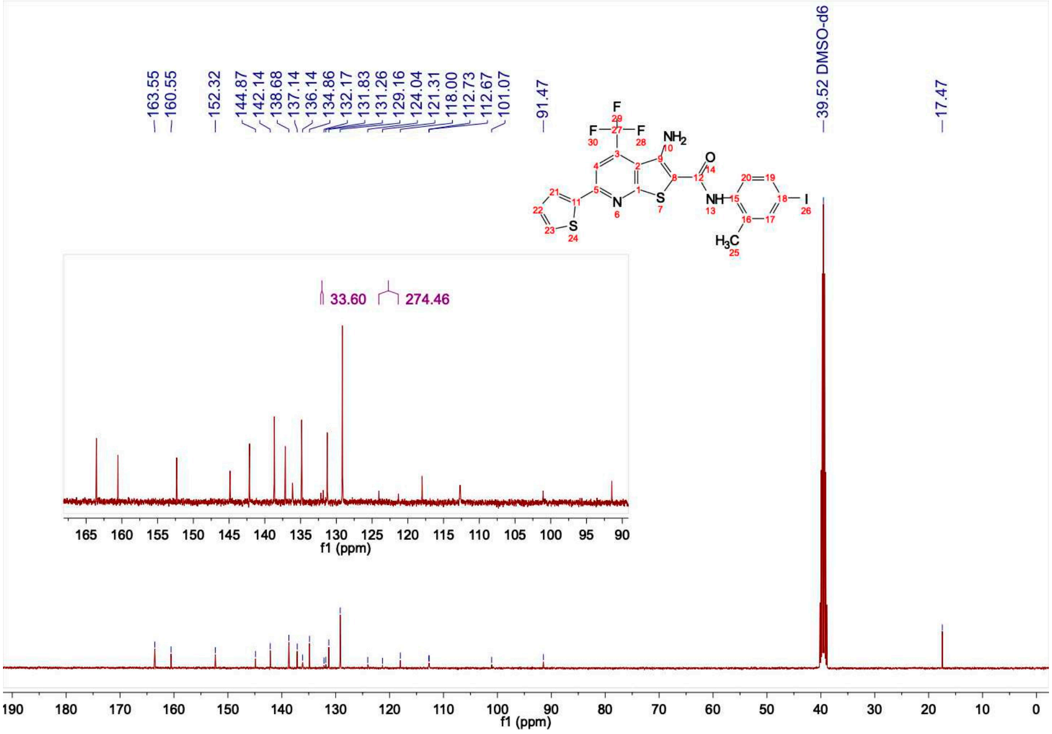

Figure S57. ^{13}C NMR (100 MHz, DMSO-d_6, δ in ppm) 3-Amino-*N*-(4-iodo-2-methylphenyl)-6-(thiophen-2-yl)-4-(trifluoromethyl)thieno[2,3-*b*]pyridine-2-carboxamide (15)

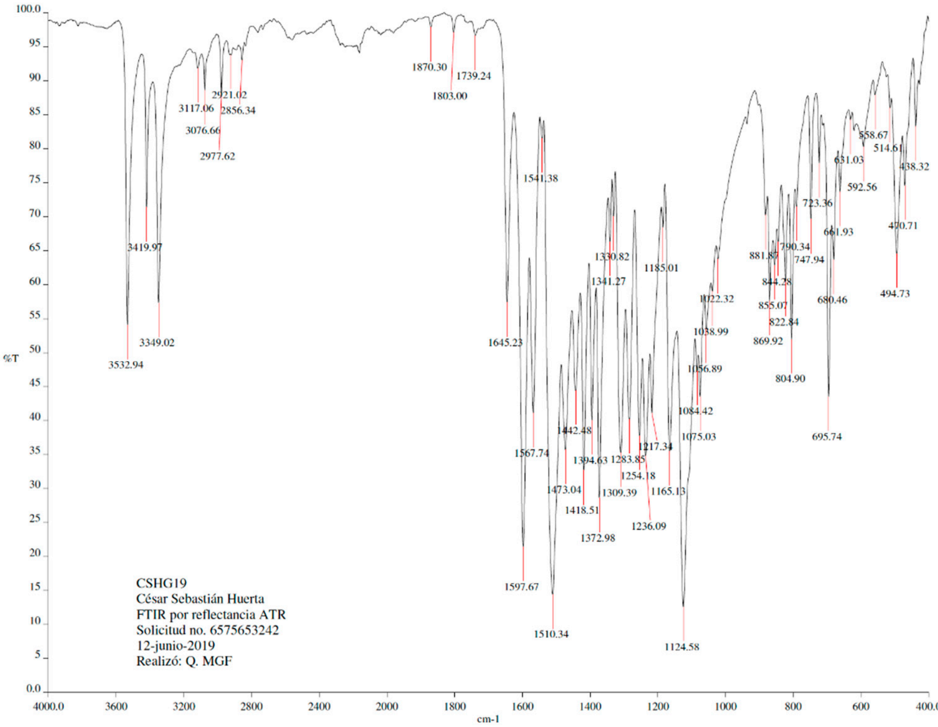

Figure S58. IR (ATR-FTIR, cm^{-1}) 3-Amino-*N*-(4-iodo-2-methylphenyl)-6-(thiophen-2-yl)-4-(trifluoromethyl)thieno[2,3-*b*]pyridine-2-carboxamide (15)

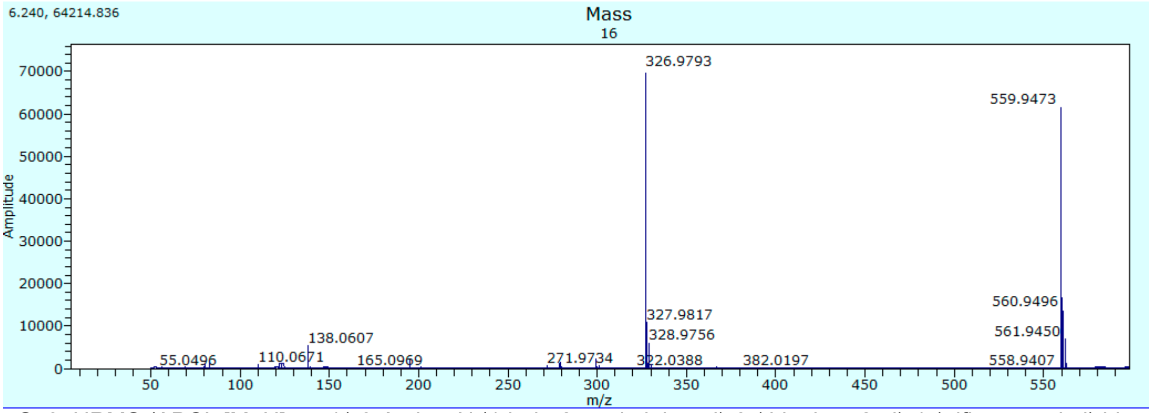

Figure S59. HRMS (APCI, [M+H]+, *m/z*) 3-Amino-*N*-(4-iodo-2-methylphenyl)-6-(thiophen-2-yl)-4-(trifluoromethyl)thieno[2,3-*b*]pyridine-2-carboxamide (15)

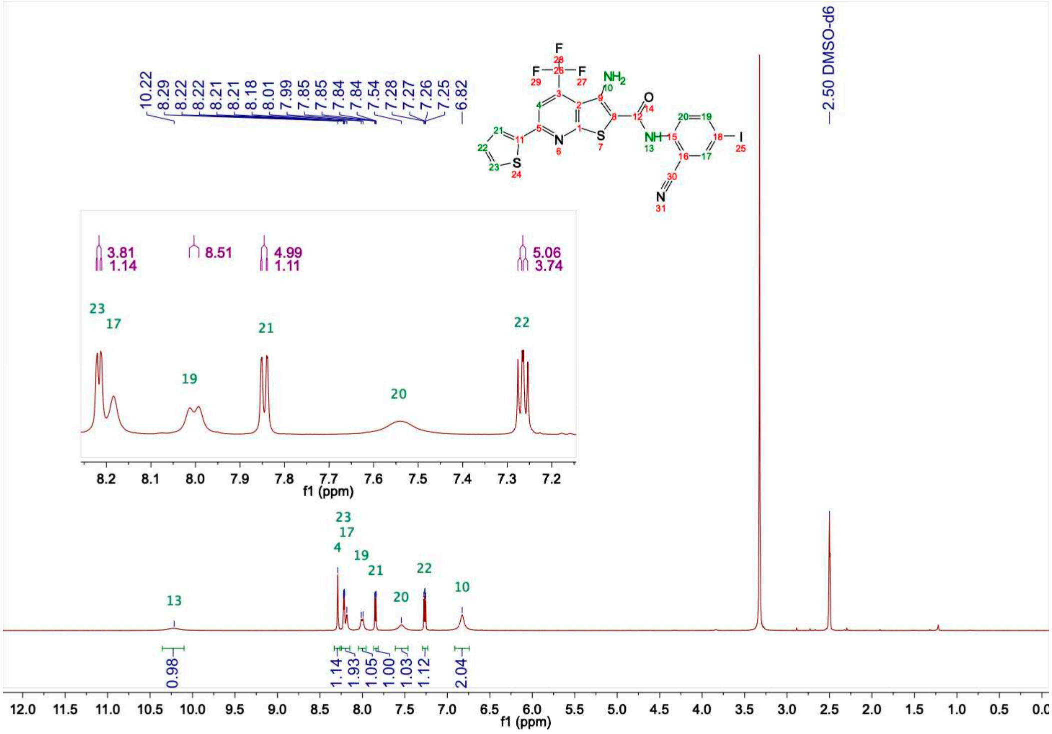

Figure S60. ^{1}H NMR (400 MHz, DMSO-d_6, δ in ppm) 3-Amino-*N*-(2-cyano-4-iodophenyl)-6-(thiophen-2-yl)-4-(trifluoromethyl)thieno[2,3-*b*]pyridine-2-carboxamide (16)

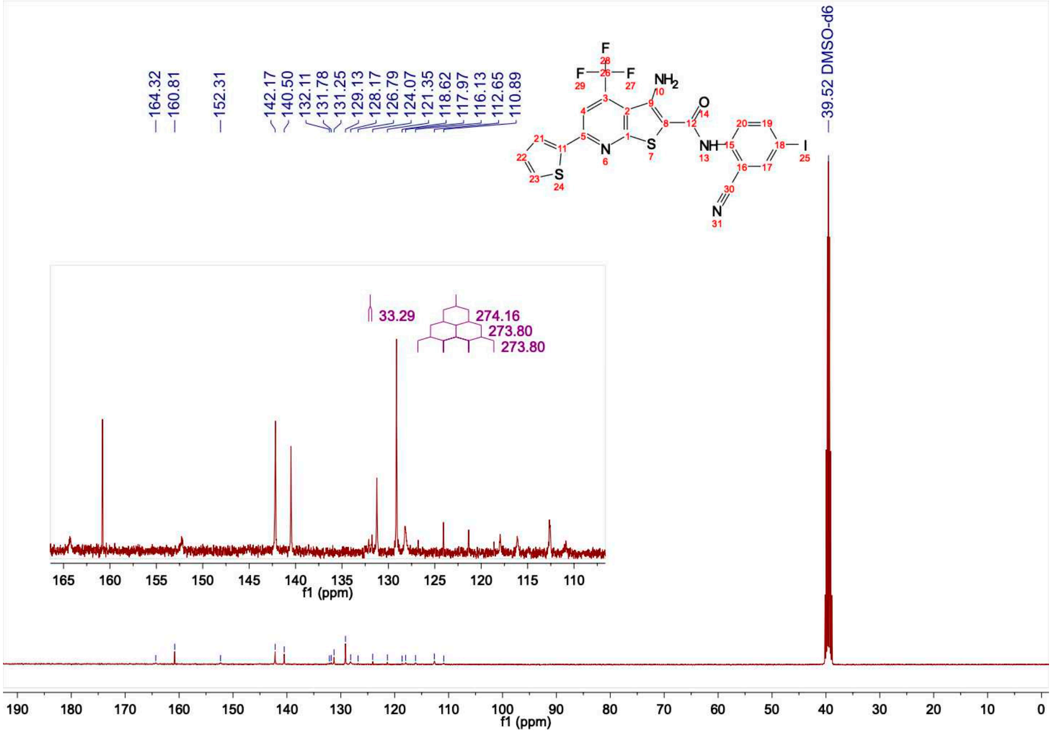

Figure S61. ^{13}C NMR (100 MHz, DMSO-d_6, δ in ppm) 3-Amino-*N*-(2-cyano-4-iodophenyl)-6-(thiophen-2-yl)-4-(trifluoromethyl)thieno[2,3-*b*]pyridine-2-carboxamide (16)

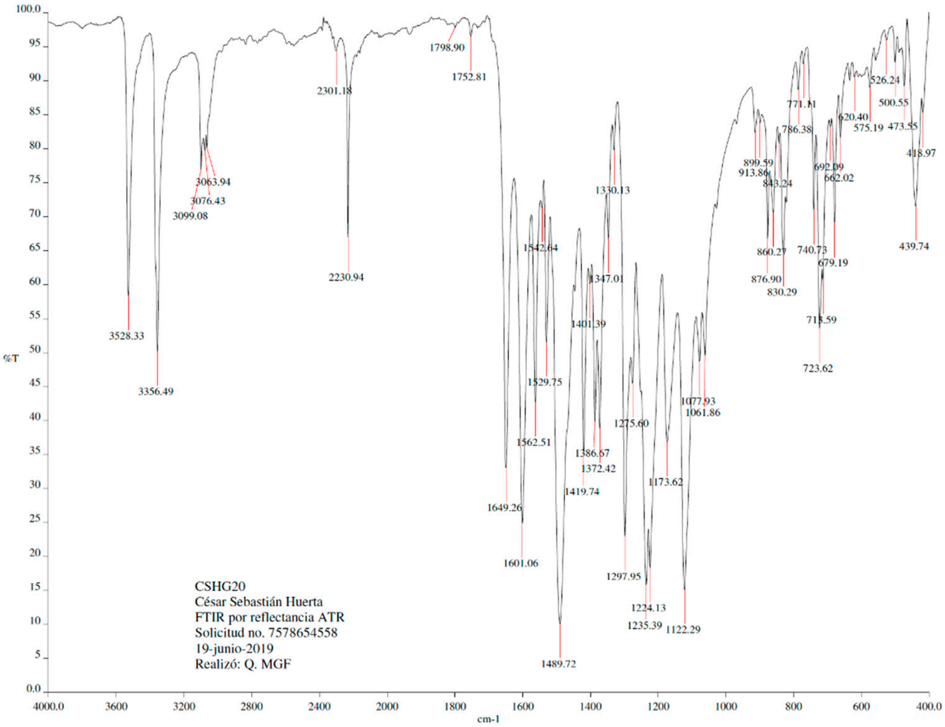

Figure S62. IR (ATR-FTIR, cm^{-1}) 3-Amino-*N*-(2-cyano-4-iodophenyl)-6-(thiophen-2-yl)-4-(trifluoromethyl)thieno[2,3-*b*]pyridine-2-carboxamide (16)

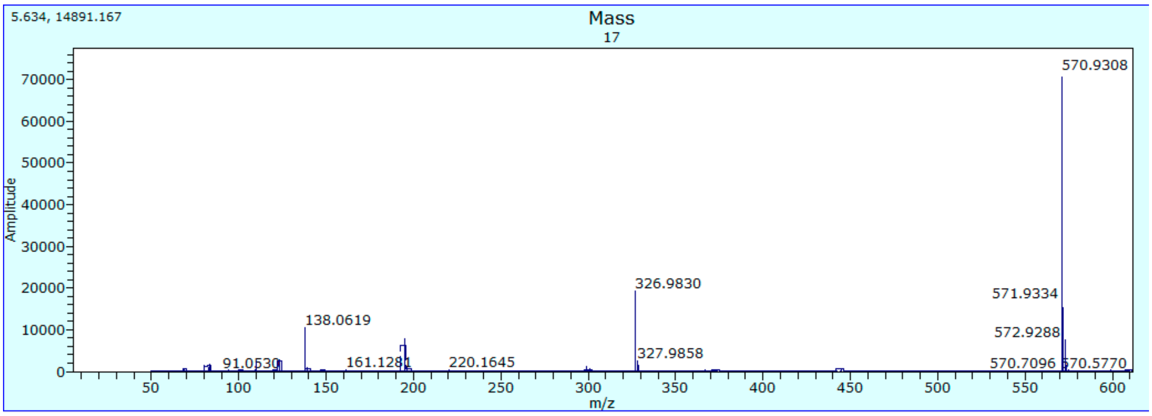

Figure S63. HRMS (APCI, $[M+H]^+$, *m/z*) 3-Amino-*N*-(2-cyano-4-iodophenyl)-6-(thiophen-2-yl)-4-(trifluoromethyl)thieno[2,3-*b*]pyridine-2-carboxamide (16)

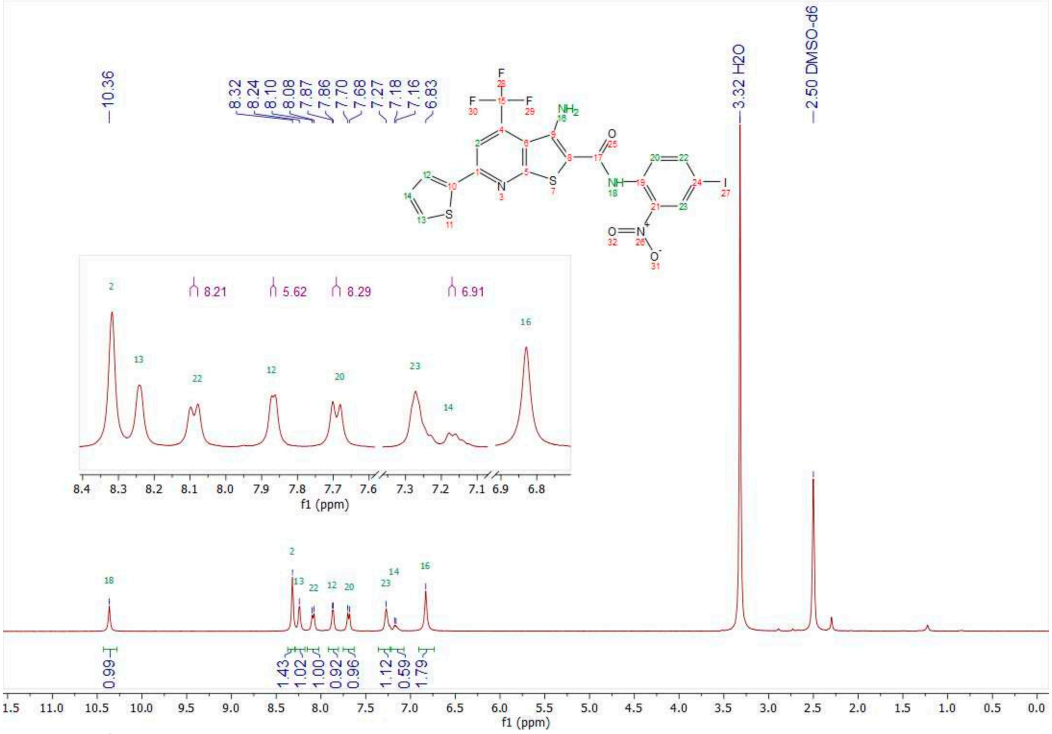

Figure S64. ^{1}H NMR (400 MHz, DMSO-d_6, δ in ppm) 3-Amino-*N*-(4-iodo-2-nitrophenyl)-6-(thiophen-2-yl)-4-(trifluoromethyl)thieno[2,3-*b*]pyridine-2-carboxamide (17)

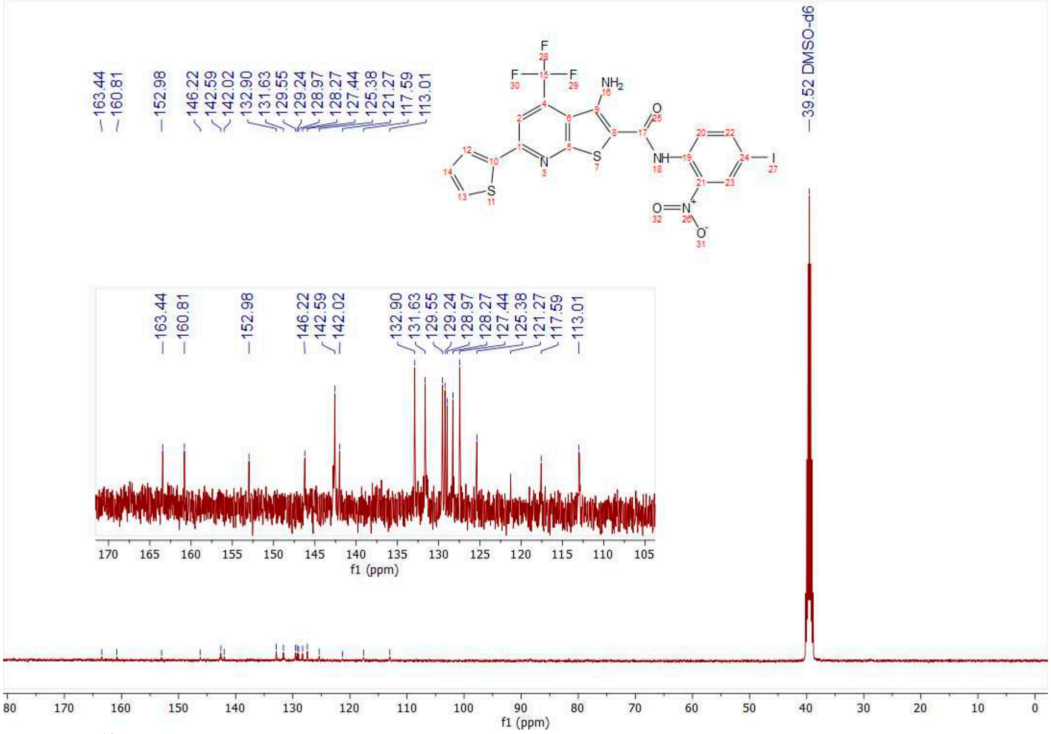

Figure S65. ^{13}C NMR (100 MHz, DMSO-d_6, δ in ppm) 3-Amino-*N*-(4-iodo-2-nitrophenyl)-6-(thiophen-2-yl)-4-(trifluoromethyl)thieno[2,3-*b*]pyridine-2-carboxamide (17)

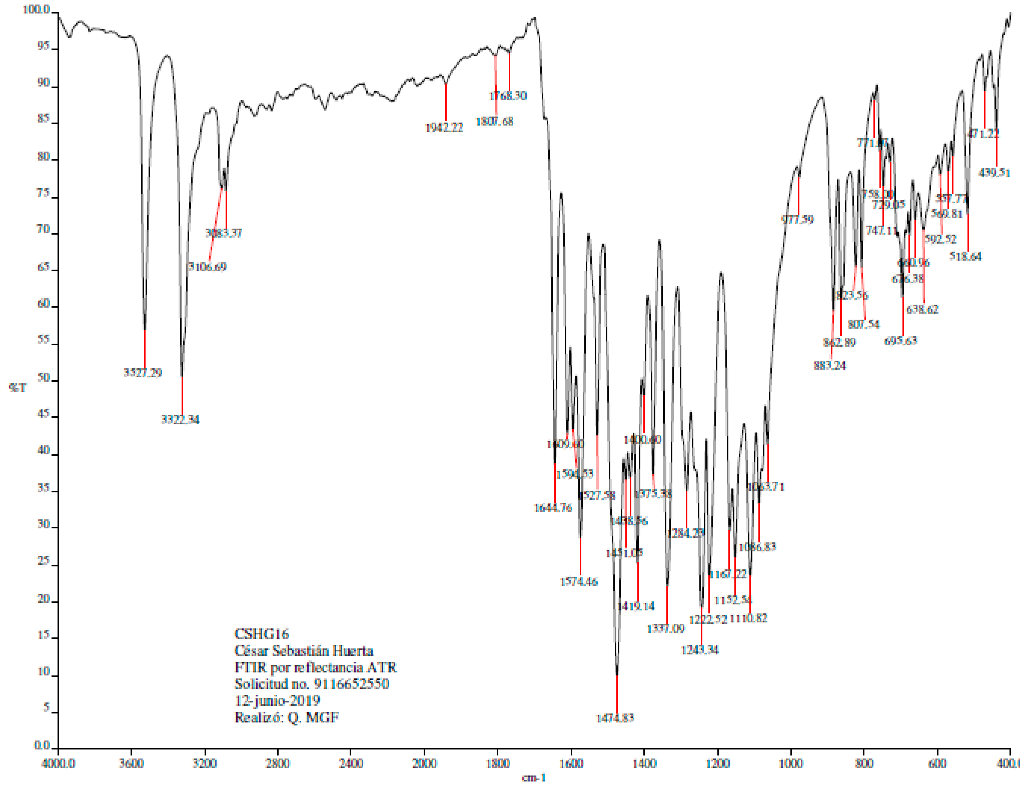

Figure S66. IR (ATR-FTIR, cm^{-1}) 3-Amino-*N*-(4-iodo-2-nitrophenyl)-6-(thiophen-2-yl)-4-(trifluoromethyl)thieno[2,3-*b*]pyridine-2-carboxamide (17)

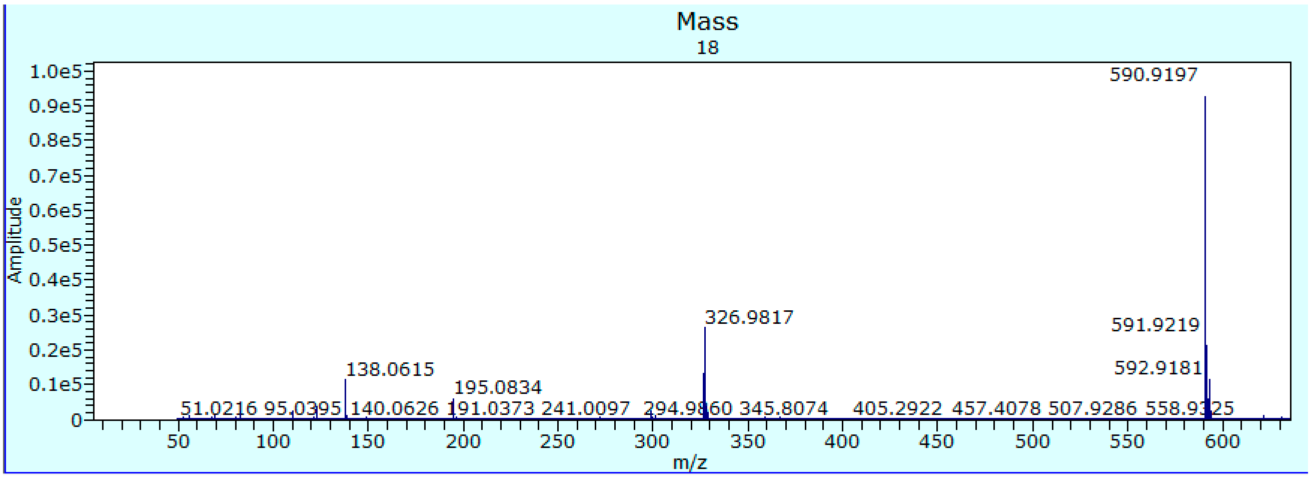

Figure S67. HRMS (APCI, [M+H]$^+$, *m/z*) 3-Amino-*N*-(4-iodo-2-nitrophenyl)-6-(thiophen-2-yl)-4-(trifluoromethyl)thieno[2,3-*b*]pyridine-2-carboxamide (17)

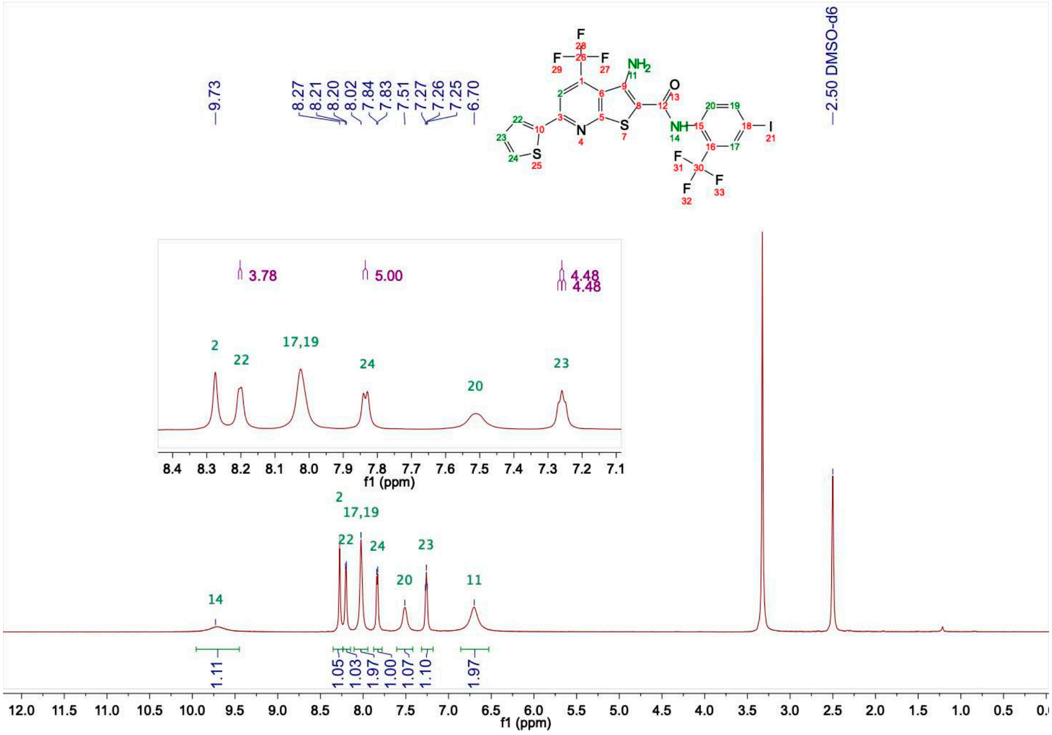

Figure S68. ^{1}H NMR (400 MHz, DMSO-d_6, δ in ppm) 3-Amino-*N*-(4-iodo-2-(trifluoromethyl)phenyl)-6-(thiophen-2-yl)-4-(trifluoromethyl)thieno[2,3-*b*]pyridine-2-carboxamide (18)

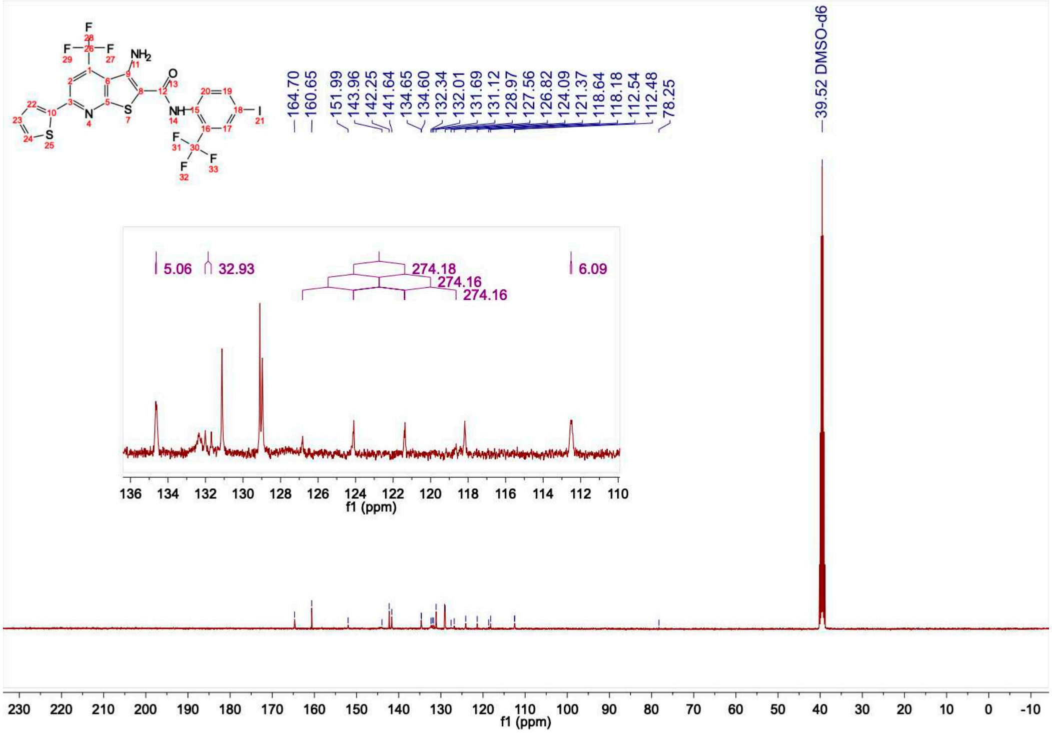

Figure S69. ^{13}C NMR (100 MHz, DMSO-d_6, δ in ppm) 3-Amino-*N*-(4-iodo-2-(trifluoromethyl)phenyl)-6-(thiophen-2-yl)-4-(trifluoromethyl)thieno[2,3-*b*]pyridine-2-carboxamide (18)

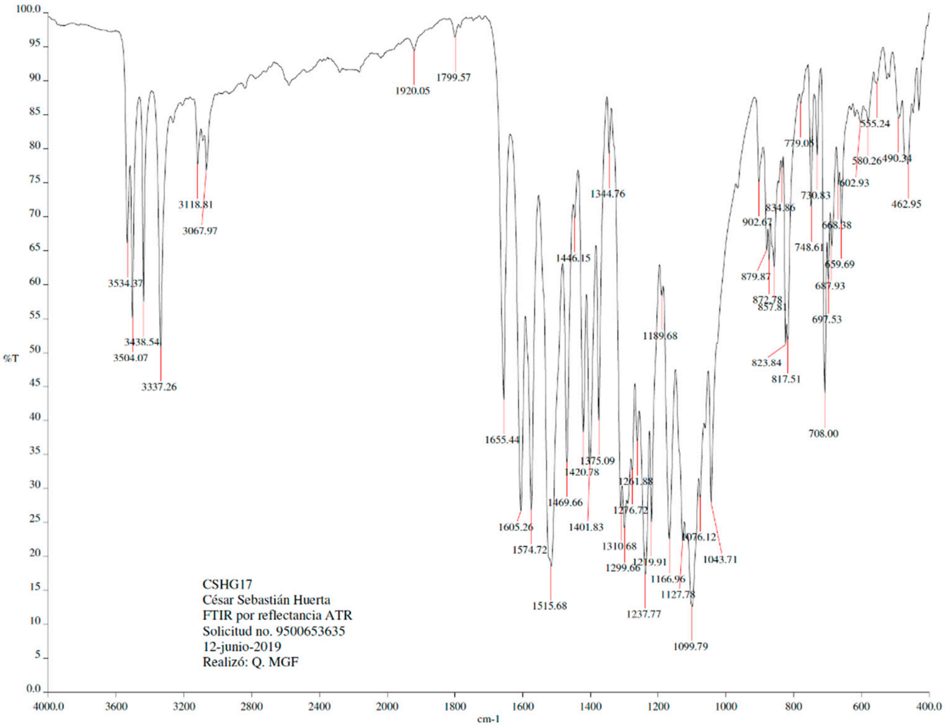

Figure S70. IR (ATR-FTIR, cm^{-1}) 3-Amino-*N*-(4-iodo-2-(trifluoromethyl)phenyl)-6-(thiophen-2-yl)-4-(trifluoromethyl)thieno[2,3-*b*]pyridine-2-carboxamide (18)

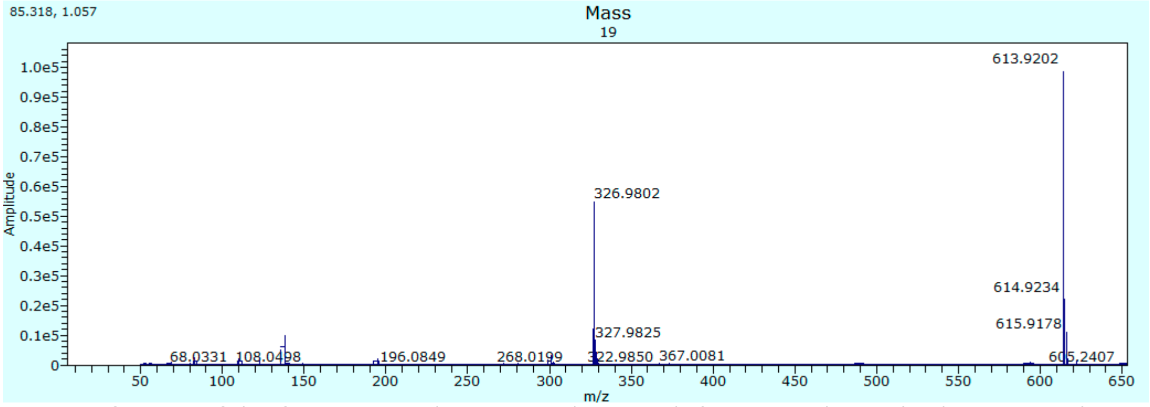

Figure S71. HRMS (APCI, [M+H]+, *m/z*) 3-Amino-*N*-(4-iodo-2-(trifluoromethyl)phenyl)-6-(thiophen-2-yl)-4-(trifluoromethyl)thieno[2,3-*b*]pyridine-2-carboxamide (18)

2. Western Blot

2.1. Blot images

Repetition 1 FOXM1

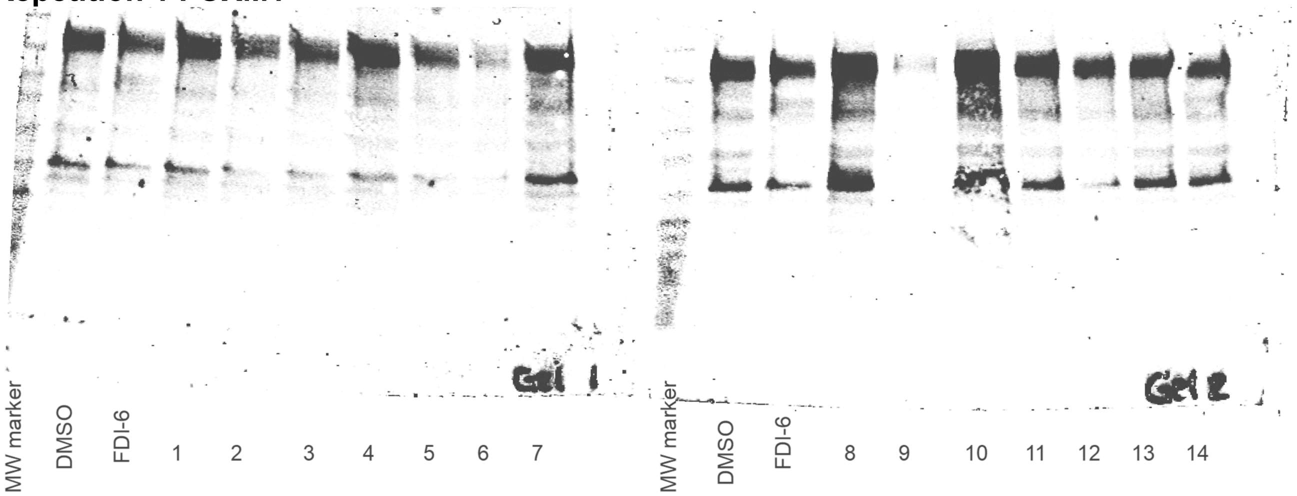

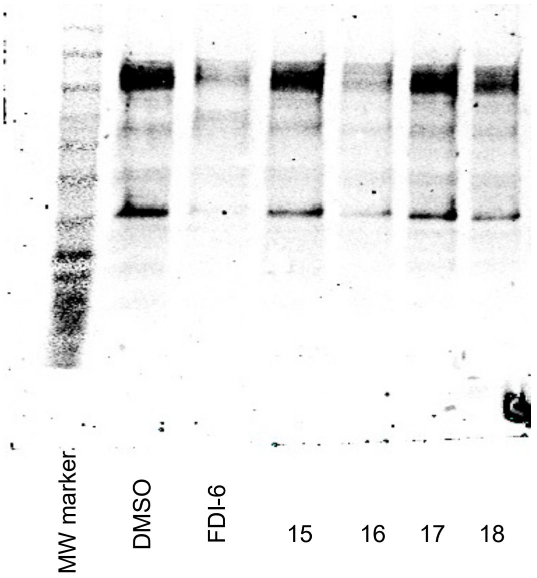

Repetition 1 β-actin

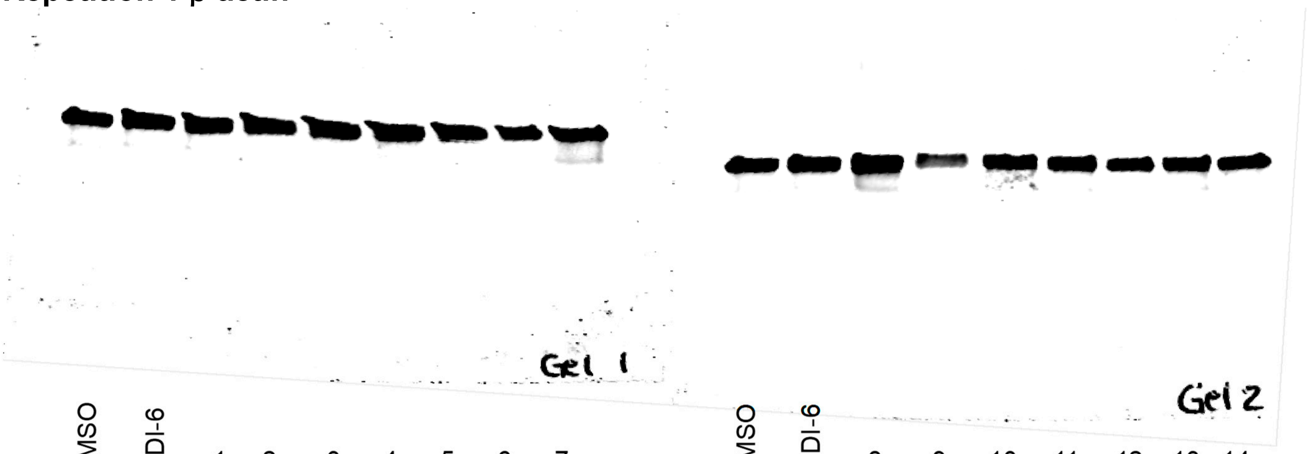

DMSO FDI-6 1 2 3 4 5 6 7 DMSO FDI-6 8 9 10 11 12 13 14

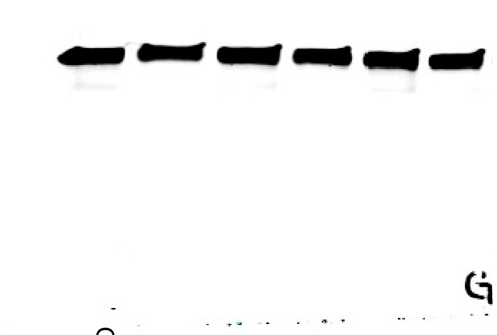

DMSO FDI-6 15 16 17 18

Repetition 2 FOXM1

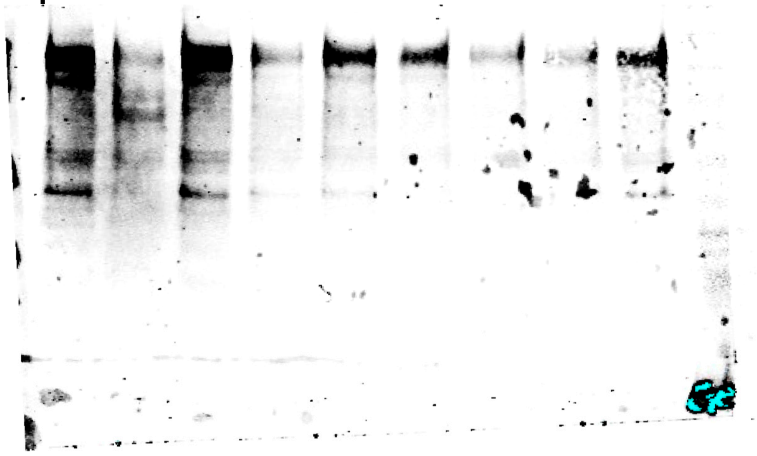

DMSO FDI-6 1 2 3 4 5 6 7 MW marker

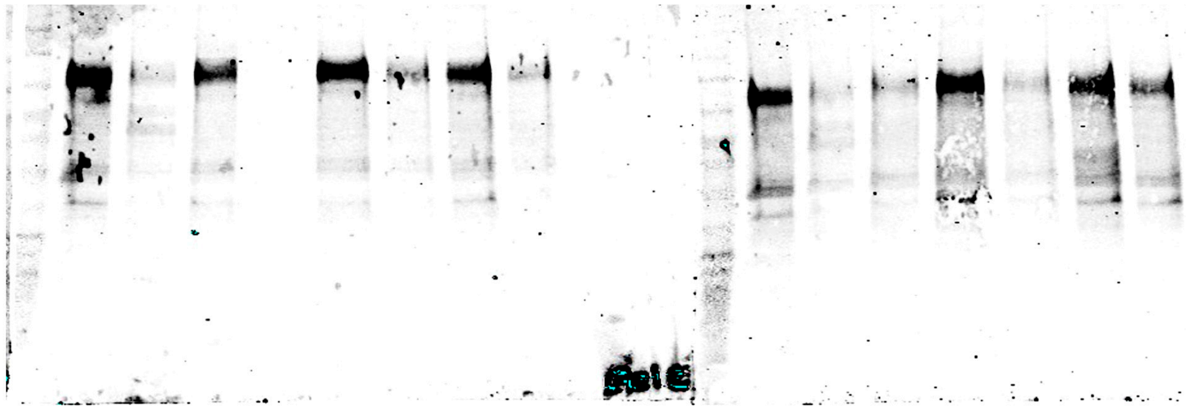

MW marker DMSO FDI-6 8 9 10 11 12 13 MW marker DMSO FDI-6 14 15 16 17 18

Repetition 2 β-actin

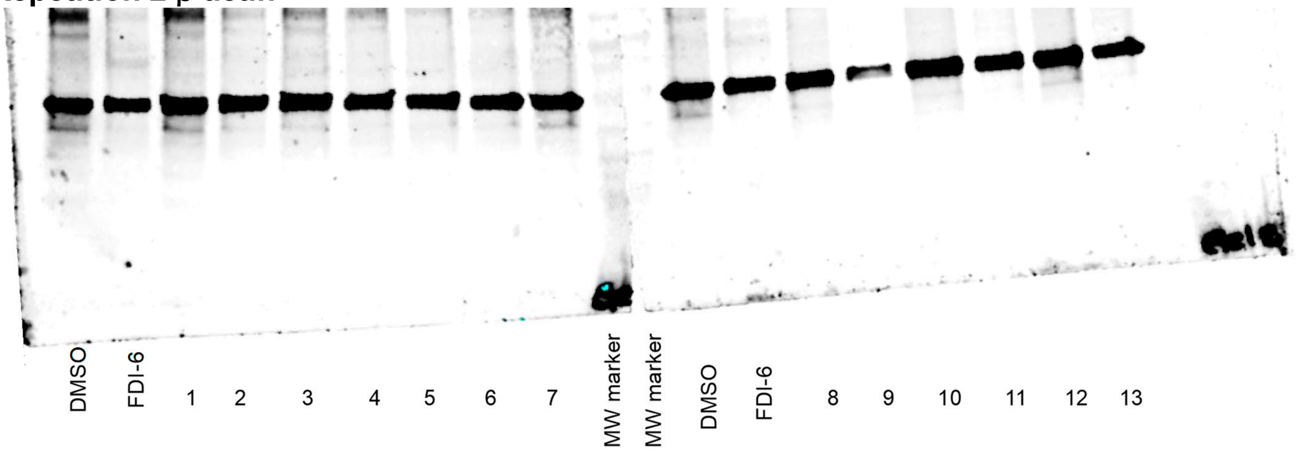

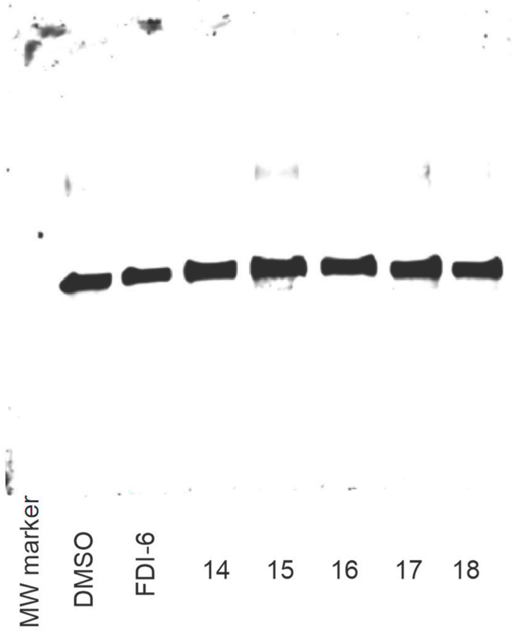

Repetition 3 FOXM1

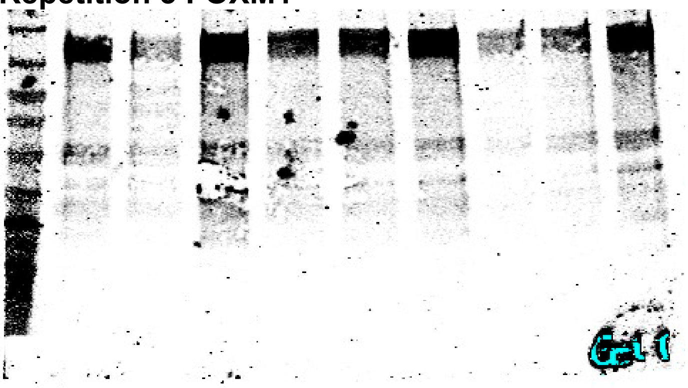

MW marker DMSO FDI-6 1 2 3 4 5 6 7

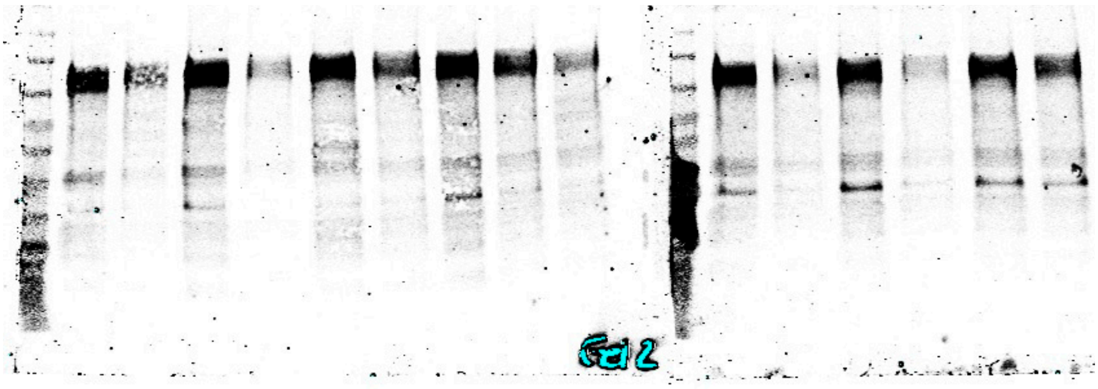

MW marker DMSO FDI-6 8 9 10 11 12 13 14 MW marker DMSO FDI-6 15 16 17 18

Repetition 3 β-actin

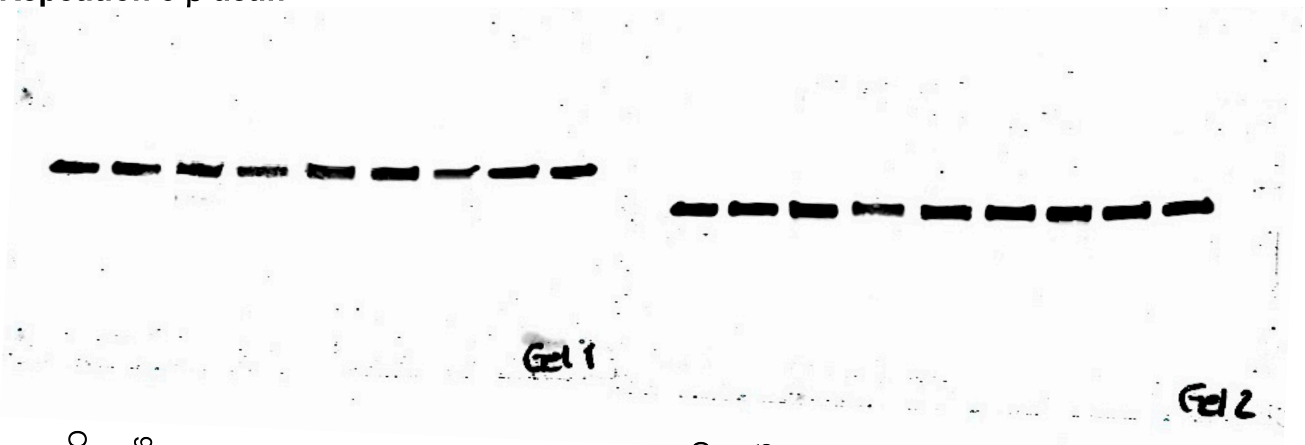

DMSO FDI-6 1 2 3 4 5 6 7

DMSO FDI-6 8 9 10 11 12 13 14

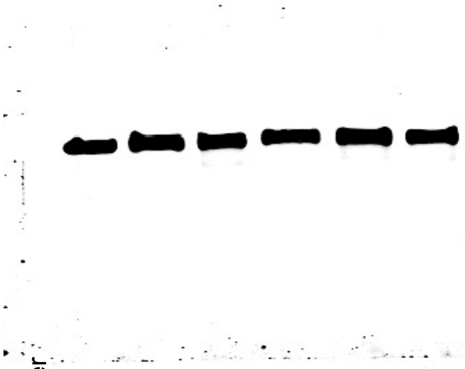

MW marker DMSO FDI-6 15 16 17 18

3. Cell proliferation inhibition (MTT) assay

3.1. Cell viability curves

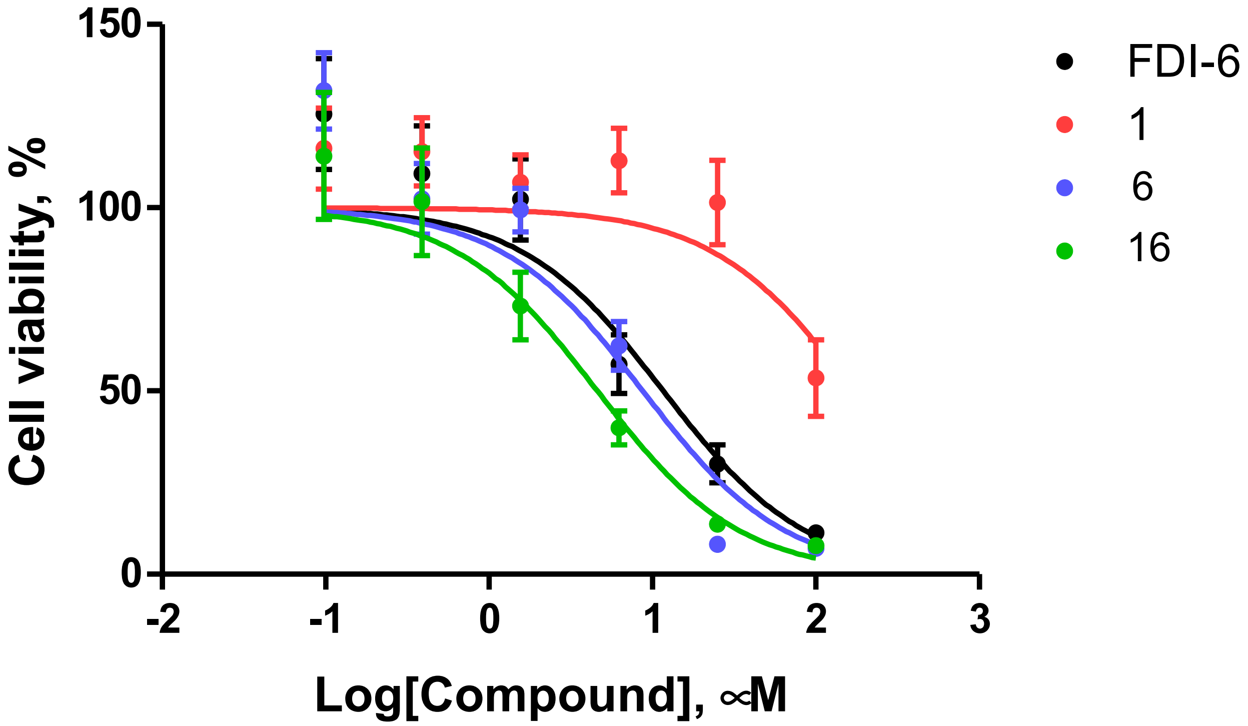

Figure S72. Cell viability curves calculated in the MTT assay for compounds **FDI-6**, **1**, **6** and **16**.

3.2. Cell viability results for each concentration

Repetition 1

Table S2. Cell viability (%) obtained at different concentrations of compounds **FDI-6**, **1**, **6**, **16** during first repetition.

	% of cell viability					
**Compounds**	100 μM	25 μM	6.25 μM	1.56 μM	0.39 μM	0.098 μM
FDI-6	10.88981	20.6482	36.48792	167.7313	94.61402	118.6565
FDI-6	7.919859	26.30524	67.74308	90.93695	90.6541	95.74543
FDI-6	8.909841	68.16735	87.82557	142.6989	198.9864	245.0913
1	84.00707	109.6052	129.8291	90.6541	140.7189	112.858
1	110.4537	145.1031	119.9293	107.2009	146.2345	186.2581
1	133.5062	162.3571	148.9216	97.01827	126.8592	143.9717
6	8.909841	7.778433	15.27401	77.0772	58.40896	156.2758
6	8.202711	12.02121	53.60047	122.0507	122.475	136.9004
6	7.212728	11.03123	75.80436	131.8091	142.5575	170.5598
16	9.899823	10.88981	16.264	27.57808	26.72952	40.30642
16	11.03123	11.45551	29.41662	46.81202	67.03595	125.1621
16	9.475545	9.051267	16.12257	22.34532	24.18385	54.73188

Repetition 2

Table S3. Cell viability (%) obtained at different concentrations of compounds **FDI-6**, **1**, **6**, **16** during second repetition.

	% of cell viability					
**Compounds**	100 μM	25 μM	6.25 μM	1.56 μM	0.39 μM	0.098 μM
FDI-6	6.772277	20.31683	54.53465	100.5149	114.0594	119.4059
FDI-6	16.53861	58.38416	101.7267	114.2733	115.8416	117.4812
FDI-6	6.70099	43.62772	84.04752	123.0416	104.0792	96.16634
1	29.86931	126.8198	142.3604	100.4436	126.7485	123.3267
1	49.04554	110.9941	133.0931	113.8455	135.3743	137.2277
1	37.14059	107.3584	117.8376	108.4277	142.5743	124.895
6	4.134653	4.491089	72.14257	93.81386	91.60396	103.9366
6	4.063366	6.059406	72.28515	91.88911	100.5149	102.6535
6	4.063366	5.275248	76.84752	104.6495	101.3703	92.31683
16	4.633663	6.986139	41.34653	77.5604	83.0495	92.10297
16	4.491089	10.5505	41.77426	74.2099	102.1545	79.7703
16	4.134653	14.68515	36.14257	68.79208	90.60594	116.0554

Repetition 3

Table S4. Cell viability (%) obtained at different concentrations of compounds **FDI-6**, **1**, **6**, **16** during third repetition.

	% of cell viability					
**Compounds**	100 μM	25 μM	6.25 μM	1.56 μM	0.39 μM	0.098 μM
FDI-6	7.553041	15.36068	33.01273	80.28289	84.61103	139.0948
FDI-6	12.72984	25.20509	65.26167	93.43706	110.4102	124.2433
FDI-6	9.165488	35.72843	78.7553	105.7426	120.2546	142.1499
1	37.25601	90.80622	113.8048	131.2871	86.81754	99.54738
1	42.94201	125.0919	125.8557	141.5559	134.3423	132.5601
1	26.22348	106.1669	118.4724	146.9873	125.0919	130.099
6	5.261669	5.346535	77.82178	84.01697	52.19236	97.25601
6	5.176803	5.601132	82.57426	110.2405	84.10184	85.12023
6	6.195191	7.722772	80.53748	104.0453	88.42999	127.3833
16	5.176803	13.74823	63.05516	92.50354	109.5615	94.54031
16	4.837341	22.74399	48.03395	73.91796	98.18953	83.25318
16	4.922207	29.6181	65.09194	88.68458	111.4286	96.91655

4. Docking

4.1. Complete energy results

Table S5. Energies and RMSD obtained from the molecular docking calculation. **S** corresponds to the final docking score. **rmsd_refine** is the root mean square deviation between the pose before refinement and the pose after refinement. **E_conf** is the energy of the conformer obtained at the end of the refinement. **E_place** is the score from the placement stage. **E_score1** is the score from London dG stage 1. **E_refine** is the score from the refinement stage, calculated to be the sum of the van der Waals electrostatics and solvation energies under the GBVI model. All the energies are presented in kcal/mol.

Compound	S	rmsd_refine	E_conf	E_place	E_score1	E_refine
**FDI-6**	-5.338305	1.4773268	15.986014	-54.181206	-8.1389589	-28.678331
**FDI-6**	-5.1264253	1.7904752	15.051484	-35.39579	-7.8655863	-25.053133
**FDI-6**	-5.0144401	1.9375528	13.468188	-23.09787	-8.1365309	-23.879059
**FDI-6**	-4.9456201	1.862273	15.604932	-42.268978	-7.8110275	-24.512882
**FDI-6**	-4.8531375	1.721066	12.64887	-68.037888	-7.9474559	-25.31728
**1**	-5.6401668	1.5524713	16.261621	-71.629776	-8.4427633	-30.043001
**1**	-5.4492126	2.0793235	17.167767	-61.984421	-8.3012581	-28.207247
**1**	-5.1827374	1.9676967	18.990822	-65.499367	-7.7281141	-25.667036
**1**	-5.1686006	1.7830374	15.88992	-57.518822	-8.2673788	-26.819695
**1**	-5.1165023	2.4796474	16.575996	-58.095146	-8.5071297	-25.853733
**2**	-5.4899082	1.7592049	21.566185	-57.890968	-8.3963432	-26.42877
**2**	-5.3872046	1.869249	23.766672	-44.657082	-8.4432869	-29.233871
**2**	-5.2979212	2.0906069	21.227957	-64.199402	-9.1749802	-27.297512
**2**	-5.2569742	1.5443414	22.852812	-45.536835	-8.2044468	-28.146954
**2**	-5.2259846	1.4202759	18.812019	-33.107525	-8.0280008	-25.587824
**3**	-5.9180169	1.9166759	33.345818	-63.216045	-9.1858463	-30.859615
**3**	-5.6129642	1.5264744	32.948364	-21.212303	-9.7483625	-27.584579
**3**	-5.5340776	1.1248676	32.231762	-35.67205	-8.5899515	-29.307955
**3**	-5.5323825	1.6735625	30.833076	-20.270655	-8.4682693	-29.380529
**3**	-5.4525037	1.2816342	30.118441	-41.476482	-8.2064276	-25.155724
**4**	-5.4390607	1.4054714	31.762026	-40.819653	-8.7666054	-25.083143
**4**	-5.3563433	2.0869746	26.824017	-23.414131	-8.0852318	-29.324467
**4**	-5.3474154	1.4627774	29.75893	-57.65107	-9.0094824	-25.754499
**4**	-5.3234344	1.5491819	31.897501	-63.929676	-8.2776403	-27.496273
**4**	-5.317296	1.86622	28.928391	-39.825756	-8.6837711	-26.494179
**5**	-5.5565462	1.6122432	15.661255	-69.274788	-8.2181501	-29.775429
**5**	-5.3517618	2.3519318	15.438794	-18.485668	-8.3850422	-27.490812
**5**	-5.2614355	1.8375982	14.099689	-37.870232	-8.1018248	-26.724401
**5**	-5.2313776	1.2452521	17.781315	-17.762663	-8.6190996	-25.473431
**5**	-5.1865044	2.3625171	14.120085	-28.749651	-8.6924171	-23.974689
**6**	-7.2806792	4.1656809	26.755617	-28.867189	-7.9340611	-44.91996
**6**	-5.6367393	1.4237808	21.976625	-78.823204	-8.3023853	-29.27865
**6**	-5.5057325	2.0781171	24.45314	-54.739441	-7.8214002	-29.662445

**6**	-5.4004521	1.9047606	20.918114	-33.038445	-8.7913685	-28.297958
**6**	-5.3352289	2.4080846	20.640421	-28.148298	-8.2777824	-26.19659
**7**	-6.1223764	1.8541651	34.839344	-65.561066	-10.000466	-33.109962
**7**	-5.5682316	1.2983443	34.31126	-67.029503	-8.157177	-29.742929
**7**	-5.5161242	1.602182	30.789888	-28.082367	-8.0810022	-29.514164
**7**	-5.4952044	1.5528752	33.950855	-39.40247	-8.7854662	-25.073668
**7**	-5.4905944	1.8140582	29.556953	-49.814346	-9.4725342	-28.560659
**8**	-5.5822921	2.838567	31.178745	-34.063137	-8.1277618	-27.292181
**8**	-5.3927989	1.9500358	28.387878	-48.16161	-8.1798677	-27.35808
**8**	-5.3865566	2.1593928	30.631142	-28.792112	-8.3226824	-28.833075
**8**	-5.3605027	2.1547613	30.490473	-16.130714	-8.3304892	-28.563957
**8**	-5.3316803	1.3900253	30.687817	-65.423866	-8.393589	-27.809975
**9**	-5.6117167	1.1444713	17.487101	-71.070732	-8.0715332	-30.368109
**9**	-5.3821702	1.7715225	17.953995	-28.10075	-8.1204739	-26.915285
**9**	-5.3699656	0.85840809	16.01136	-71.781998	-8.6568327	-28.441198
**9**	-5.3000054	0.9992246	18.35759	-25.55896	-8.3087234	-25.605536
**9**	-5.1997032	1.1550051	18.081516	-59.9533	-8.4549456	-26.686287
**10**	-5.8423109	2.0992353	16.323935	-48.279106	-8.5883608	-31.157759
**10**	-5.7419567	2.2620227	15.062777	-50.601212	-8.1739378	-29.656292
**10**	-5.6038198	1.7609154	17.346361	-78.00029	-8.0608749	-29.533369
**10**	-5.5908046	1.9944097	18.548309	-56.505825	-7.8871999	-29.306259
**10**	-5.52631	1.9276125	18.591242	-65.526924	-8.0392971	-29.354624
**11**	-5.9348149	2.0744538	23.605753	-21.52593	-8.2371664	-40.158108
**11**	-5.5808821	1.1508025	26.280411	-55.589626	-8.2182417	-30.539635
**11**	-5.4907632	1.6021212	24.755617	-64.74556	-8.5392733	-28.949594
**11**	-5.398108	1.9107034	22.924856	-67.551384	-8.1293821	-27.070906
**11**	-5.3779955	1.4997498	23.321672	-62.975185	-8.6371946	-26.795973
**12**	-6.1844773	2.4288316	37.993782	-55.595215	-9.3844099	-33.852421
**12**	-5.5789404	1.3979168	36.134808	-72.986465	-9.3607359	-28.523031
**12**	-5.5558009	1.4617552	38.79121	-31.541012	-8.1773767	-26.175003
**12**	-5.5245342	1.539789	38.628479	-54.12048	-8.548295	-30.145285
**12**	-5.5086131	3.5019641	34.483746	-29.3312	-8.1678896	-31.651628
**13**	-5.7708998	1.816542	34.121777	-14.365557	-8.6024485	-27.123755
**13**	-5.428916	1.227232	30.16321	-19.114666	-9.6633196	-28.873335
**13**	-5.311501	1.6246878	29.418001	-50.753136	-8.2141151	-27.346237
**13**	-5.3093481	1.8084203	33.238441	-57.815647	-7.8808236	-25.771421
**13**	-5.2685499	1.2828549	29.258812	-31.650736	-8.9178534	-24.715807
**14**	-5.6385722	1.8541728	17.185652	-52.194252	-8.1697369	-30.934959
**14**	-5.4539962	1.6615111	18.732416	-21.375572	-8.0905771	-26.04801
**14**	-5.4069648	1.5242864	18.99151	-33.283913	-8.2104864	-28.272657
**14**	-5.3554974	1.8045828	16.208561	-60.348892	-8.2591305	-27.271912

**14**	-5.2349787	1.7281722	16.876963	-28.15233	-8.6276598	-23.831312
**15**	-5.5661087	1.2627839	19.373938	-48.184063	-7.8292184	-29.823318
**15**	-5.4128032	1.3402346	15.384918	-68.584457	-7.9898005	-28.188866
**15**	-5.4035006	0.91580033	19.027111	-35.244614	-8.9236469	-25.509529
**15**	-5.324903	0.70152098	18.775999	-31.465891	-7.8591814	-28.182518
**15**	-5.3080263	1.1217449	15.324822	-53.465458	-8.6888409	-25.325354
**16**	-5.831728	0.83068651	28.68158	-42.803955	-7.996912	-36.087429
**16**	-5.7144165	1.8981289	25.565731	-72.53685	-8.7451544	-28.164516
**16**	-5.6375232	1.7709608	27.834364	-44.666153	-9.3822699	-30.743147
**16**	-5.4634309	1.3873246	25.827208	-30.360121	-9.8122549	-25.712955
**16**	-5.4512801	1.5541686	24.718439	-64.518372	-8.3356628	-27.367441
**17**	-5.7236748	2.0985909	36.322975	-42.392044	-8.8288469	-27.970745
**17**	-5.6258636	1.4173553	38.188519	-42.192471	-9.7375422	-28.814939
**17**	-5.620862	1.1158218	39.459923	-26.936701	-8.3025618	-26.068855
**17**	-5.5670137	1.5614127	36.213585	-32.835323	-7.998013	-27.341677
**17**	-5.5206585	1.7426802	34.716862	-42.95248	-9.5609159	-28.84234
**18**	-5.7931991	1.94192	35.006351	-56.105999	-8.5065718	-30.702761
**18**	-5.7241273	1.7199305	35.150059	-24.39172	-7.9546518	-27.556942
**18**	-5.6704545	1.7326242	32.351482	-16.87336	-8.0047407	-30.290895
**18**	-5.5318828	1.1511974	36.470264	-35.824757	-8.1994667	-28.077065
**18**	-5.4603996	1.850363	31.74235	-16.049095	-8.2164488	-25.739573

4.2. Best docking pose of each compound

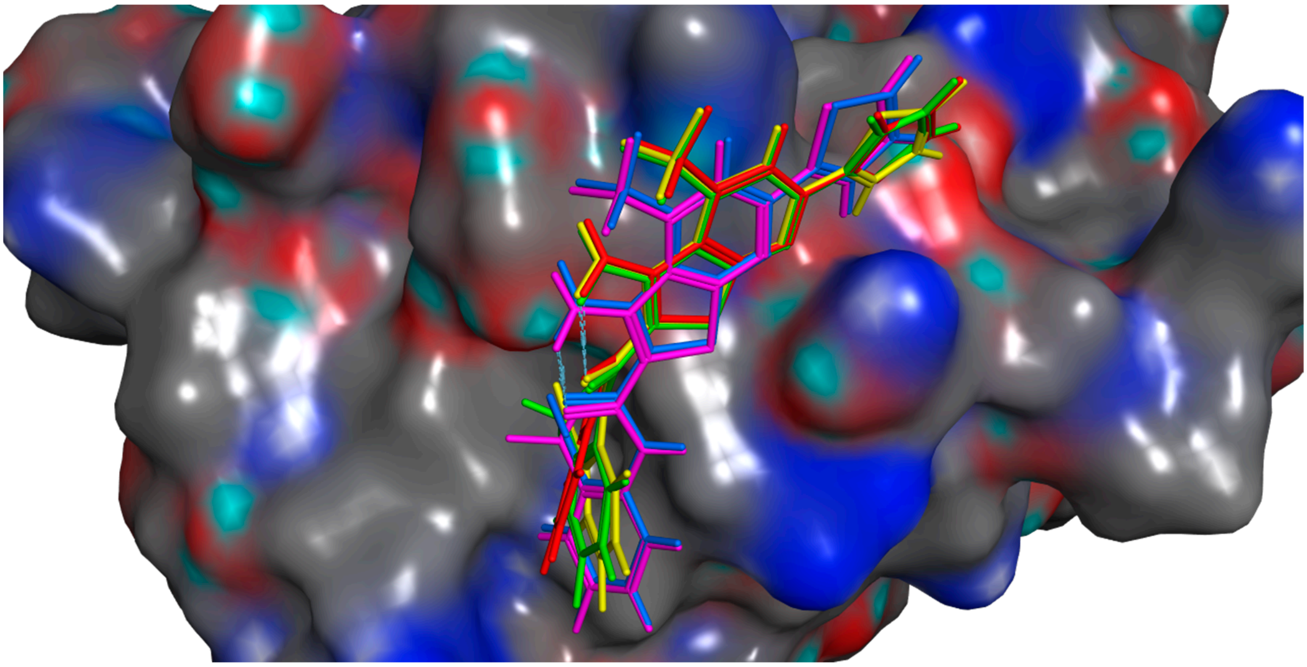

Figure S73. Docking poses from the compounds **1** (green), **2** (blue), **3** (yellow) and **4** (pink) compared to **FDI-6** (red) in the FOXM1-DBD.

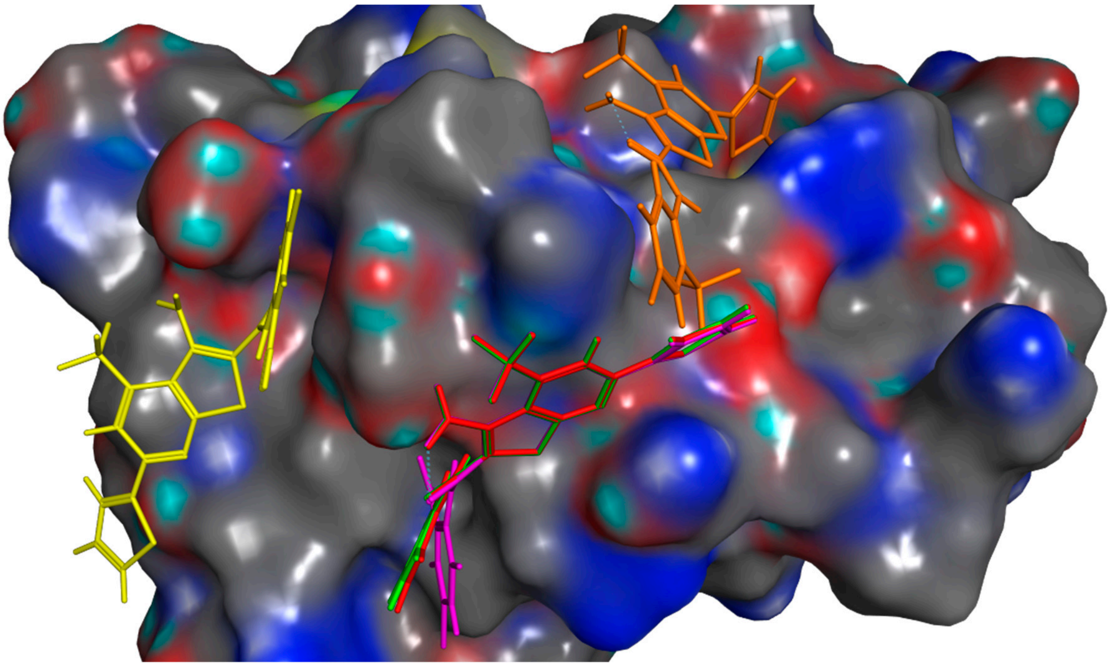

Figure S74. Docking poses from the compounds **5** (green), **6** (yellow), **7** (pink) and **8** (orange) compared to **FDI-6** (red) in the FOXM1-DBD.

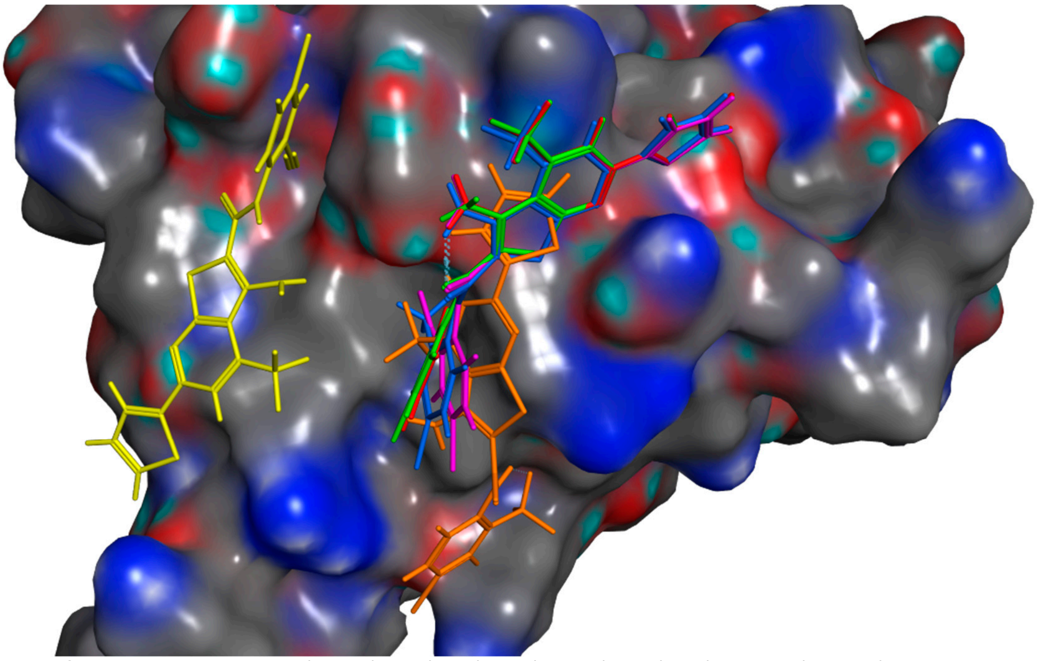

Figure S75. Docking poses from the compounds **9** (green), **10** (blue), **11** (yellow), **12** (pink) and **13** (orange) compared to **FDI-6** (red) in the FOXM1-DBD.

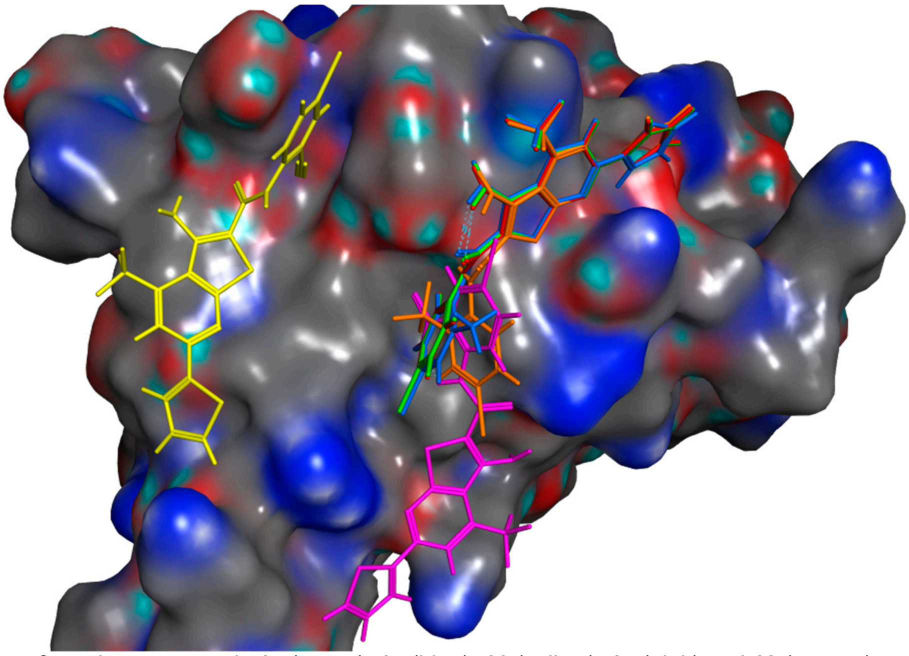

Figure S76. Docking poses from the compounds **14** (green), **15** (blue), **16** (yellow), **17** (pink) and **18** (orange) compared to **FDI-6** (red) in the FOXM1-DBD.

5. MEP maps

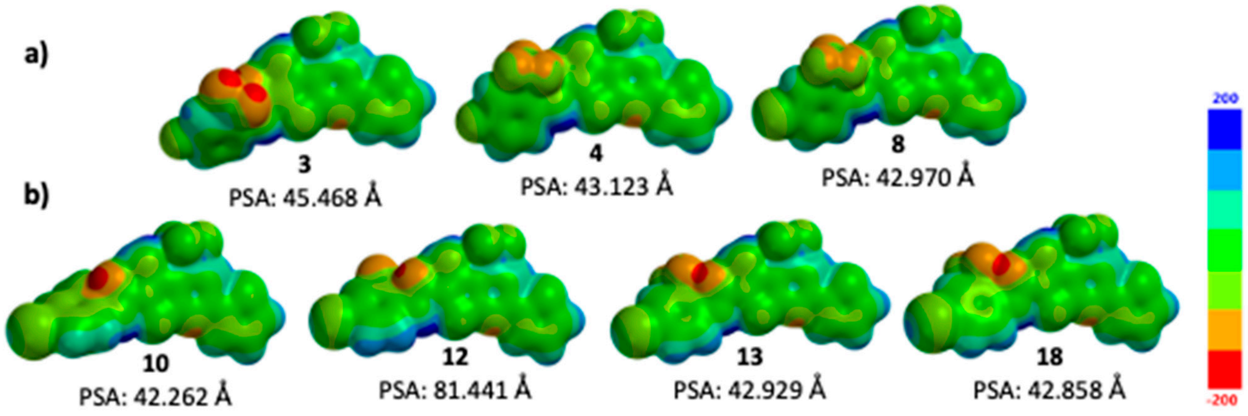

Figure S77. Polar surface area and **MEP** maps superimposed onto the total electron density at a value of 0.002 e^{-}/au^{3}

Table S6. MEP values of selected thieno[2,3-*b*]pyridines.

MEP values (kJ/mol)[a]						
	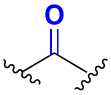	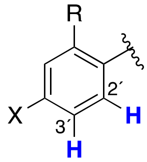	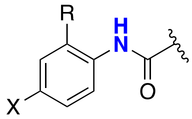	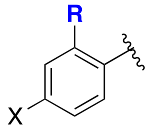	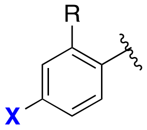	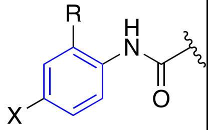
**FDI-6**	-112.0	44.0	195.0	---	-75.8	-50.0 to 17.4
**5**	-117.4	83.4	219.0	---	-78.0 to 11.3	-65.0 to 4.1
**9**	-111.4	81.4	209.0	---	-78.4 to 46.0	-65.0 to 0.4
**14**	-109.0	77.4	215.3	---	-74.1 to 91.0	-69.7 to -14.7
**2**	-128.7	108.2	211.4	-174.0	12.6 to 46.2	-5.0 to 37.5
**6**	-154.1	110.0	222.5	-181.0	-40.0 to 49.0	-7.0 to 50.0
**11**	-165.0	98.8	220.0	-175.0	-46.9 to 78.5	-18.1 to 51.3
**16**	-160.0	108.8	215.0	-189.0	-46.2 to 123.4	-23.2 to 54.7
**1**	-167.6	75.3	197.4	2.3	-76.0	-104 to 59
**7**	-151.0	119.3	236.0	-163.0	-42.4 to 46.2	-7.0 to 30.4
**15**	-157.1	76.4	206.0	-9.4	-8.6 to 95	-72.0 to 3.0
**17**	-146.3	112.0	232.4	-158.8	-42.8 to 123.0	-36.4 to 58.1

[a] The average MEP values correspond to that part of the molecule (top row) depicted in blue that showed a different MEP value from FDI-6 and parent molecules. For a more detailed list of values, please see Table S5.

6. Additional References

1. Wang, N.-Y.; Zuo, W.-Q.; Xu, Y.; Gao, C.; Zeng, X.-X.; Zhang, L.-D.; You, X.-Y.; Peng, C.-T.; Shen, Y.; Yang, S.-Y.; et al. Discovery and Structure–Activity Relationships Study of Novel Thieno[2,3-b]Pyridine Analogues as Hepatitis C Virus Inhibitors. *Bioorg. Med. Chem. Lett.* **2014**, *24*, 1581–1588, doi:10.1016/j.bmcl.2014.01.075.
2. Tabatabaei Dakhili, S.A.; Pérez, D.J.; Gopal, K.; Tabatabaei Dakhili, S.Y.; Ussher, J.R.; Velázquez-Martínez, C.A. A Structure-Activity Relationship Study of Forkhead Domain Inhibitors (FDI): The Importance of Halogen Binding Interactions. *Bioorg. Chem.* **2019**, *93*, 103269, doi:10.1016/j.bioorg.2019.103269.
